# Dual-immunotherapy triumphs: redefining deficient mismatch repair or high microsatellite instability metastatic colorectal cancer first-line treatment

**DOI:** 10.1038/s41392-025-02322-8

**Published:** 2025-07-15

**Authors:** Zhijun Yuan, Saimeng Shi, Shanshan Weng

**Affiliations:** 1 Department of Medical Oncology, Key Laboratory of Cancer Prevention and Intervention, Ministry of Education, The Second Affiliated Hospital, Zhejiang University School of Medicine, Hangzhou, Zhejiang China; 2 Department of Pathology and Pathophysiology, School of Medicine, Zhejiang University, Hangzhou, China; 3 Cancer Institute, The Second Affiliated Hospital, Zhejiang University School of Medicine, Hangzhou, Zhejiang China

**Keywords:** Gastrointestinal cancer, Tumour immunology, Cancer

In a recent paper published in *Lancet* by Thierry Andre et al., CheckMate 8HW (NCT04008030) revealed that dual-agent immunotherapy improved the prognosis of deficient mismatch repair or high microsatellite instability (dMMR/MSI-H) metastatic colorectal cancer (mCRC) significantly.^1^ The results emphasize the significant therapeutic advantages of combining nivolumab (programmed death-1 inhibitor) and ipilimumab (cytotoxic T-lymphocyte-associated antigen-4 inhibitor) in treating dMMR/MSI-H mCRC, irrespective of comparison with chemotherapy (first-line) or nivolumab monotherapy (all treatment lines).

dMMR/MSI-H CRC comprises 10–15% of non-metastatic CRC and 5% of mCRC. The advent of immune checkpoint inhibitors (ICIs) has revolutionized the treatment of dMMR/MSI-H tumors, which are characterized by the accumulation of somatic mutations and the generation of tumor-specific antigens that create an immunogenic “hot” tumor microenvironment. Pembrolizumab, as demonstrated in KEYNOTE-177 (NCT02563002), is the current standard first-line treatment for dMMR/MSI-H mCRC, but the objective response rate (ORR) was only 45.1%, and the progressive disease (PD) rate was higher than that with chemotherapy (29.4% vs. 12.3%). Moreover, its progression-free survival (PFS) showed a early-phase crossover. In contrast, the combination of nivolumab and ipilimumab achieved a higher ORR of 62% and a lower PD rate of 16% in first-line treatment in a phase II clinical trial (CheckMate-142).

CheckMate 8HW is a randomized, open-label, international, phase III trial evaluating the efficacy of nivolumab plus ipilimumab in dMMR/MSI-H mCRC. Patients enrolled for the first time would be stratified according to tumor location and prior systemic therapy (0, 1, or ≥2) and then randomly assigned to receive nivolumab + ipilimumab, nivolumab, or chemotherapy with or without targeted therapy. The immunotherapy regimen consisted of a 12-week induction phase (dual-agent every 3 weeks, mono-agent every 2 weeks) followed by a nivolumab maintenance phase (480 mg every 4 weeks for up to 2 years). The primary endpoint was set as independent dual PFS in dMMR/MSI-H mCRC patients with centrally confirmed, comparing dual-agent immunotherapy versus chemotherapy (first-line) or nivolumab monotherapy (all lines), as assessed by blinded independent central review (BICR).

A total of 839 dMMR/MSI-H mCRC (across all lines) who had not previously received immunotherapy were enrolled and randomized to receive nivolumab + ipilimumab (*n* = 354), nivolumab (*n* = 353), or chemotherapy (*n* = 132). One independent primary endpoint were first presented at the 2024 American Society of Clinical Oncology Gastrointestinal Cancers Symposium (ASCO-GI) and subsequently published in *The New England Journal of Medicine*.^2^ Recent data indicate that nivolumab + ipilimumab continues to demonstrate a significant PFS benefit (hazard ratio [HR]: 0.21; 95% confidence interval [CI]: 0.14 to 0.31) compared to first-line chemotherapy in centrally confirmed dMMR/MSI-H mCRC.^1^ Moreover, PFS benefits were consistently observed across all prespecified subgroups.

Finally, the other primary endpoint were officially announced at the ASCO-GI conference in January 2025. Of the 707 randomized patients, 296 centrally confirmed dMMR/MSI-H mCRC were assigned to the dual-agent group, and 286 to the mono-agent group. Compared to nivolumab (median PFS: 39.3 month [22.1 - not estimable]), nivolumab + ipilimumab (not reached [53.8 - not estimable]) significantly improved PFS (HR: 0.62 [0.48–0.81], *p* = 0.0003). And the ORR of dual-agent (71% vs. 58%, *p* = 0.0011) was also significantly higher than that of mono-agent.

Overall, the data for nivolumab across all lines demonstrated superior PFS and ORR, as well as a lower PD rate compared to pembrolizumab in the first-line treatment (Table 1). This may be attributed to CheckMate 8HW’s emphasis on the cantrally retesting the dMMR/MSI-H status. Indeed, the early-phase crossover of PFS in centrally confirmed dMMR/MSI-H mCRC is not obvious. Central confirmation revealed that 12–13% of locally assessed patients were actually mismatch repair-proficient and microsatellite stable status, and its response to immunotherapy was extremely poor (PFS: dual-agent 1.9 months [1.5–5.8] versus chemotherapy 11.5 months [2.0 - not estimable]; HR: 1.6 [0.7–3.7]).^3^ Therefore, in clinical practice, it is crucial to re-evaluate the dMMR/MSI-H results with reliable methods before selecting immunotherapy for patients.

**Table 1:** Comparison of two major phase III studies of dMMR/MSI-H mCRC immunotherapy

	Nivolumab plus Ipilimumab (CheckMate 8HW, First Line)^1,3^	Pembrolizumab (KEYNOTE 177, First Line)	Nivolumab plus Ipilimumab (CheckMate 8HW, All Line)^2^	Nivolumab (CheckMate 8HW, All Line)
**Efficacy**				
PFS	54.1 (54.1 - NE)^a^	16.5 (5.4–38.1)^a^	NR (53.8 - NE)	39.3 (22.1 - NE)
12 m PFS	79% (72–84)	55.3% (47.0–62.9)	76% (71–80)	63% (57–68)
24 m PFS	74% (67–80)^a^	48.3% (39.9–56.2)	71% (65–76)	56% (49–61)
36 m PFS	69% (61–76)^a^	42.3% (34.0–50.4)	68% (62–73)	51% (45–57)
OS	-	NR (49.2 - NE)	-	-
ORR	-	45.1% (37.1–53.3)	71% (65–76)	58% (52–64)
CR rate	-	13.1%	30%	28%
PD rate	-	29.4%	10%	19%
**Special subgroup (HR)**^b^	versus chemotherapy	versus chemotherapy		
Overall	HR: 0.21 (0.14–0.31)^a^	HR: 0.60 (0.45–0.80)	HR: 0.63 (0.49–0.82)	
Wide type *BRAF/RAS*	-	HR: 0.44 (0.29–0.67)	HR: 0.64 (0.39–1.06)	
*KRAS* / *NRAS* mutation	HR: 0.24 (0.09–0.63)	HR: 1.19 (0.68–2.07)	HR: 0.76 (0.44–1.31)	
*BRAF* V600E	-	HR: 0.50 (0.31–0.80)	HR: 0.62 (0.39–0.97)	
Liver metastases	HR: 0.11 (0.05–0.25)	-	HR: 0.68 (0.44 -1.03)	
Lung metastases	-	-	-	
Peritoneal metastases	HR: 0.19 (0.10–0.34)	-	HR: 0.55 (0.37–0.82)	
Lynch syndrome	-	-	HR: 0.90 (0.47–1.72)	

^a^The latest data is recorded

^b^The HR value for PFS was presented

All parentheses indicate the 95% confidence interval. *PFS* progression free survival, *OS* overall survival, *ORR* objective response rate, *CR* complete response, *PD* progressive disease, *HR* hazard ratio, *NE* not estimable, *NR* not reached

CheckMate 8HW randomized the participants according to different treatment lines and allowed cross-treatment. With the extension of the follow-up time, the first-line advantage of immunotherapy is likely to be further demonstrated. According to the newly released PFS2 data,^4^ first-line dual-agent can reduce the risk of death or progression after first subsequent therapy by 72% (HR: 0.28 [0.18–0.44]), and dual-agent across all lines also has advantages over nivolumab (HR: 0.57 [0.42–0.78]). Additionally, there are many questions that remain to be addressed, such as whether patients who progress after mono-agent immunotherapy can benefit again from dual-agent immunotherapy, and whether patients who achieve long-term benefits and discontinue immunotherapy can undergo rechallenge with immunotherapy in the event of recurrence or progression.

Subgroup analysis in randomized controlled trial is a crucial methodology for identifying subpopulations that may benefit from therapeutic interventions. In CheckMate 8HW, regardless of whether *RAS*/*BRAF* mutations or liver metastases, first-line dual-agent immunotherapy demonstrated a significant improvement in PFS across all predefined subgroups. When comparing mono-agent and dual-agent across all treatment lines (Table 1), further benefits could be observed in subgroups with *BRAF* mutations, liver metastases, and peritoneal metastases, while benefits were limited in subgroups with *RAS* mutations and Lynch syndrome. In summary, the combination of nivolumab and ipilimumab can serve as a first-line treatment regimen that benefits various subgroups through different anti-immune evasion mechanisms. In addition, the latest data shows that the first-line dual-agent immunotherapy has also revealed significant advantages in PFS (HR: 0.03 [< 0.01–0.28]) and PFS2 (HR: 0.63 [0.09–4.52]) in the Asian population.^5^

The adverse effects (AEs) of dual-agent immunotherapy have long been a barrier to its widespread adoption. Through continuous dose optimization, the CheckMate 8HW study ultimately set the regimen as nivolumab 240 mg plus ipilimumab 1 mg/kg. At this dose intensity, the completion rate within the first 12 weeks reached 82%, with no new safety concerns emerging. Additionally, compared to dual-agent, the discontinuation rate due to AEs with nivolumab mono-agent was reduced from 14% to 6%, while the overall efficacy data across all treatment lines remained comparable to those of pembrolizumab in the first-line setting. Although the overall toxicity is manageable, immune-related adverse events (such as myocarditis or severe colitis) can still be life-threatening, especially in elderly patients or those with underlying diseases or organ dysfunction. Therefore, treatment strategies must be personalized based on the patient’s individual condition and the specific types of AEs encountered.

Finally, dual-agent immunotherapy, built on its significant ORR advantage, also achieves a complete response (CR) rate of 30% across all treatment lines. When patients are in good physical condition and the therapy is used in the first-line or even neoadjuvant setting, there is potential for further improvement in CR rates. Nivolumab plus ipilimumab is expected to become one of the preferred immunotherapy regimens for locally advanced dMMR/MSI-H CRC, offering significant potential for improving cure rates, organ preservation, and even avoiding surgery.

Overall, the CheckMate 8HW trial underscores the superiority of nivolumab combined with ipilimumab in improving PFS in dMMR/MSI-H mCRC, alongside a favorable safety profile. These findings solidify the role of dual-agent immunotherapy as a new standard of care, providing a more effective therapeutic option for patients with this challenging disease.

## Supplementary information

REF1

REF2

REF3

REF4

REF5
