# Supplementary material for: Dual-immunotherapy triumphs: redefining deficient mismatch repair or high microsatellite instability metastatic colorectal cancer first-line treatment
Source: Signal Transduct Target Ther. 2025 Jul 15;10:234. doi: 10.1038/s41392-025-02322-8 (PMC12264035; doi:10.1038/s41392-025-02322-8)
Supplement: Supplementary file 1 — REF1 [file 41392_2025_2322_MOESM1_ESM.pdf]

Check for updates

Nivolumab (NIVO) plus ipilimumab (IPI) vs chemotherapy (chemo) or NIVO monotherapy for microsatellite instability-high/mismatch repair-deficient (MSI-H/dMMR) metastatic colorectal cancer (mCRC): Expanded analyses from CheckMate 8HW.

Heinz-Josef Lenz, Sara Lonardi, Elena Elez, Lars Henrik Jensen, Eric Van Cutsem, Yann Touchefeu, Rocio Garcia-Carbonero, David Tougeron, Guillermo Mendez, Michael Schenker, Christelle De La Fouchardiere, Maria Luisa Limon, Takayuki Yoshino, Jin Li, Francine Aubin, Elvis Cela, Li Li, Rachel Tam, Lixian Jin, Thierry Andre; University of Southern California Norris Comprehensive Cancer Center, Los Angeles, CA; Istituto Oncologico Veneto IOV-IRCCS, Padua, Italy; Vall d'Hebron University Hospital and Institute of Oncology (VHIO), Barcelona, Spain; University Hospital of Southern Denmark, Vejle Hospital, Vejle, Denmark; University Hospitals Gasthuisberg and University of Leuven (KU Leuven), Leuven, Belgium; Centre Hospitalier Universitaire de Nantes, Nantes, France; Hospital Universitario 12 de Octubre, Imas12, UCM, Madrid, Spain; Centre Hospitalier Universitaire de Poitiers, Poitiers, France; Fundacion Favaloro, Buenos Aires, Argentina; Centrul de Oncologie Sf. Nectarie, Craiova, Romania; Centre Léon Bérard, Lyon, France; Hospital Universitario Virgen del Rocio, Seville, Spain; National Cancer Center Hospital East, Chiba, Japan; Shanghai East Hospital, Shanghai, China; Centre Hospitalier de l'Université de Montréal, Montreal, QC, Canada; Bristol Myers Squibb, Princeton, NJ; Sorbonne Université, and Hôpital Saint Antoine, Assistance Publique Hôpitaux de Paris, Paris, France

Downloaded from ascopubs.org by 117.147.12.78 on June 26, 2025 from 117.147.012.078
Copyright © 2025 American Society of Clinical Oncology. All rights reserved.

Background: In the phase 3 CheckMate 8HW study (NCT04008030), both dual primary endpoints of progression-free survival (PFS) for first-line (1L) NIVO + IPI vs chemo (HR 0.21; $P < 0.0001$) and NIVO + IPI vs NIVO across all lines (HR 0.62; $P = 0.0003$) in patients (pts) with centrally confirmed MSI-H/dMMR mCRC were met. We report expanded analyses of NIVO + IPI vs NIVO (all lines) and longer follow-up results for NIVO + IPI vs chemo (1L). **Methods:** The study design was described previously. Pts with MSI-H/dMMR per local testing were enrolled. After randomization, IHC and PCR based tests were used for central confirmation. PFS2 (time from randomization to progression after subsequent systemic therapy, start of second subsequent systemic therapy, or death) was a key exploratory endpoint. **Results:** In all randomized pts (all lines), 296 of 354 (84%) in the NIVO + IPI arm, 286 of 353 (81%) in the NIVO arm, and 113 of 132 (86%) in the chemo arm had centrally confirmed MSI-H/dMMR. In all randomized 1L pts, 171 of 202 (85%) in the NIVO + IPI arm and 84 of 101 (83%) in the chemo arm had centrally confirmed MSI-H/dMMR. Median follow-up was 47.0 mo (range 16.7–60.5). 1L NIVO + IPI continued to show PFS benefit vs chemo (Table). Subsequent systemic therapy was received by 27 (16%) and 61 (73%) pts after 1L NIVO + IPI and chemo, respectively; 10 (6%) and 21 (25%) received subsequent non-study immunotherapy. In the 1L chemo arm, 39 (46%) pts crossed over to NIVO + IPI on study. PFS2 continued to favor 1L NIVO + IPI vs chemo (Table). Across all lines, NIVO + IPI demonstrated superior PFS vs NIVO (Table). Subsequent systemic therapy was received by 54 (18%) pts in the NIVO + IPI arm and 83 (29%) in the NIVO arm; 20 (7%) and 31 (11%) received subsequent non-study immunotherapy. PFS2 favored NIVO + IPI vs NIVO across all lines of therapy (Table). In all treated pts, grade 3/4 treatment-related adverse events occurred in 78 (22%) and 50 (14%) pts in the NIVO + IPI and NIVO arms, respectively. Additional analyses will be presented. **Conclusions:** NIVO + IPI demonstrated sustained clinical benefit vs chemo (1L) and NIVO (all lines) despite use of subsequent therapy, as shown by improved PFS2 in pts with centrally confirmed MSI-H/dMMR mCRC. No new safety signals were observed. These results support NIVO + IPI as a standard of care treatment for MSI-H/dMMR mCRC. Clinical trial information: NCT04008030. Research Sponsor: Bristol Myers Squibb.

**Centrally confirmed MSI-H/dMMR (1L)**	**NIVO + IPI (n = 171)**	**Chemo (n = 84)**
**Median PFS (95% CI), mo**	54.1 (54.1–NE)	5.9 (4.4–7.8)
**HR (95% CI)**	0.21 (0.14–0.31)	
**Median PFS2 (95% CI), mo**	NR (NE–NE)	30.3 (15.2–NE)
**HR (95% CI)**	0.28 (0.18–0.44)	

**Centrally confirmed MSI-H/dMMR (all lines)**	**NIVO + IPI (n = 296)**	**NIVO (n = 286)**
**Median PFS (95% CI), mo**	NR (53.8–NE)	39.3 (22.1–NE)
**HR (95% CI)**	0.62 (0.48-0.81); $P = 0.0003$	
**Median PFS2 (95% CI), mo**	NR (NE–NE)	NR (NE–NE)
**HR (95% CI)**	0.57 (0.42–0.78)	

NE, not evaluable; NR, not reached.
