# Supplementary material for: Dual-immunotherapy triumphs: redefining deficient mismatch repair or high microsatellite instability metastatic colorectal cancer first-line treatment
Source: Signal Transduct Target Ther. 2025 Jul 15;10:234. doi: 10.1038/s41392-025-02322-8 (PMC12264035; doi:10.1038/s41392-025-02322-8)
Supplement: Supplementary file 2 — REF2 [file 41392_2025_2322_MOESM2_ESM.pdf]

Nivolumab plus ipilimumab versus nivolumab in microsatellite instability-high metastatic colorectal cancer (CheckMate 8HW): a randomised, open-label, phase 3 trial

Thierry André, Elena Elez, Heinz-Josef Lenz, Lars Henrik Jensen, Yann Touchefeu, Eric Van Cutsem, Rocio Garcia-Carbonero, David Tougeron, Guillermo Ariel Mendez, Michael Schenker, Christelle de la Fouchardiere, Maria Luisa Limon, Takayuki Yoshino, Jin Li, Jose Luis Manzano Mozo, Laetitia Dahan, Giampaolo Tortora, Myriam Chalabi, Eray Goekkurt, Maria Ignez Braghiroli, Rohit Joshi, Timucin Cil, Francine Aubin, Elvis Cela, Tian Chen, Ming Lei, Lixian Jin, Steven I Blum, Sara Lonardi

Summary

Background CheckMate 8HW prespecified dual primary endpoints, assessed in patients with centrally confirmed microsatellite instability-high or mismatch repair-deficient status: progression-free survival with nivolumab plus ipilimumab compared with chemotherapy as first-line therapy and progression-free survival with nivolumab plus ipilimumab compared with nivolumab alone, regardless of previous systemic treatment for metastatic disease. In our previous report, nivolumab plus ipilimumab showed superior progression-free survival versus chemotherapy in first-line microsatellite instability-high or mismatch repair-deficient metastatic colorectal cancer in the CheckMate 8HW trial. Here, we report results from the prespecified interim analysis for the other primary endpoint of progression-free survival for nivolumab plus ipilimumab versus nivolumab across all treatment lines.

Methods CheckMate 8HW is a randomised, open-label, international, phase 3 trial at 128 hospitals and cancer centres across 23 countries. Immunotherapy-naive adults with unresectable or metastatic colorectal cancer across different lines of therapy and microsatellite instability-high or mismatch repair-deficient status per local testing were randomly assigned (2:2:1) to nivolumab plus ipilimumab (nivolumab 240 mg, ipilimumab 1 mg/kg, every 3 weeks for four doses; then nivolumab 480 mg every 4 weeks; all intravenously), nivolumab (240 mg every 2 weeks for six doses, then 480 mg every 4 weeks; all intravenously), or chemotherapy with or without targeted therapies. The dual independent primary endpoints were progression-free survival by blinded independent central review with nivolumab plus ipilimumab versus chemotherapy (first line) and progression-free survival by blinded independent central review with nivolumab plus ipilimumab versus nivolumab (all lines) in patients with centrally confirmed microsatellite instability-high or mismatch repair-deficient metastatic colorectal cancer. This study is registered with ClinicalTrials.gov (NCT04008030).

Findings Between Aug 16, 2019, and April 10, 2023, 707 patients were randomly assigned to nivolumab plus ipilimumab (n=354) or nivolumab alone (n=353). 296 (84%) of 354 patients in the nivolumab plus ipilimumab group and 286 (81%) of 353 patients in the nivolumab group were centrally confirmed to have microsatellite instability-high or mismatch repair-deficient status. At the data cutoff on Aug 28, 2024, median follow-up (from randomisation to data cutoff) was 47·0 months (IQR 38·4 to 53·2). Nivolumab plus ipilimumab treatment showed significant and clinically meaningful improvement in progression-free survival versus nivolumab (hazard ratio 0·62, 95% CI 0·48–0·81; p=0·0003). Median progression-free survival was not reached with nivolumab plus ipilimumab (95% CI 53·8 to not estimable) and was 39·3 months with nivolumab (22·1 to not estimable). Treatment-related adverse events of any grade occurred in 285 (81%) of 352 patients receiving nivolumab plus ipilimumab and in 249 (71%) of 351 patients receiving nivolumab; grade 3 or 4 treatment-related adverse events occurred in 78 (22%) and 50 (14%) patients, respectively. There were three treatment-related deaths: one event of myocarditis and pneumonitis each in the nivolumab plus ipilimumab group and one pneumonitis event in the nivolumab group.

Interpretation Nivolumab plus ipilimumab showed superior progression-free survival versus nivolumab across all treatment lines, with a manageable safety profile, in patients with microsatellite instability-high or mismatch repair-deficient metastatic colorectal cancer. These results, together with the first-line results of superior progression-free survival with nivolumab plus ipilimumab versus chemotherapy, suggest nivolumab plus ipilimumab as a potential new standard of care for patients with microsatellite instability-high or mismatch repair-deficient metastatic colorectal cancer.

Funding Bristol Myers Squibb and Ono Pharmaceutical.

Copyright © 2025 Elsevier Ltd. All rights reserved, including those for text and data mining, AI training, and similar technologies.

Lancet 2025; 405: 383–95

Published **Online** January 25, 2025 https://doi.org/10.1016/S0140-6736(24)02848-4

See **Comment** page 354

Sorbonne Université, Hôpital Saint Antoine, Assistance Publique Hôpitaux de Paris, Paris, France (Prof T Andre MD); **Unité Mixte de Recherche Scientifique 938, SIRIC CURAMUS, Paris, France** (Prof T Andre); **Vall d'Hebron University Hospital and Institute of Oncology, Universitat Autònoma de Barcelona, Barcelona, Spain** (E Elez MD); **University of Southern California Norris Comprehensive Cancer Center, Los Angeles, CA, USA** (H-J Lenz MD); **University Hospital of Southern Denmark, Vejle Hospital, Vejle, Denmark** (L H Jensen MD); **Institut des Maladies de l'Appareil Digestif, Centre Hospitalier Universitaire de Nantes, Nantes, France** (Prof Y Touchefeu MD); **University Hospitals Gasthuisberg and University of Leuven, Leuven, Belgium** (E Van Cutsem MD); **Hospital Universitario 12 de Octubre Imas12, Facultad de Medicina, UCM, Madrid, Spain** (R Garcia-Carbonero MD); **Centre Hospitalier Universitaire de Poitiers Site de la Miletrie, Poitiers, France** (D Tougeron MD); **Hospital Universitario Fundacion Favaloro, Buenos Aires, Argentina** (G A Mendez MD); **Centrul de Oncologie Sf Nectarie, Craiova, Romania** (M Schenker MD); **University of Medicine and Pharmacy, Craiova, Romania** (M Schenker); **Centre Léon Bérard, Lyon, France** (C de la Fouchardiere MD); **Hospital Universitario Virgen del Rocío, Seville, Spain** (M L Limon MD); **National Cancer Center Hospital East,**

Chiba, Japan (T Yoshino MD); **Shanghai East Hospital, Shanghai, China** (J Li MD); **Institut Català d'Oncologia, Hospital Universitario Germans Trias i Pujol, Badalona, Spain** (J L Manzano Mozo MD); **La Timone, Aix Marseille Université, Marseille, France** (L Dahan MD); **Fondazione Policlinico Universitario A Gemelli IRCCS, Rome, Italy** (G Tortora MD); **Netherlands Cancer Institute, Amsterdam, Netherlands** (M Chalabi MD); **Hematology-Oncology Practice Eppendorf and University Cancer Center Hamburg, Hamburg, Germany** (E Goekkurt MD); **Instituto do Cancer de São Paulo, Universidade de São Paulo, São Paulo, Brazil** (M I Braghiroli PhD); **Cancer Research SA, Adelaide, SA, Australia** (R Joshi MD); **University of Health Sciences, Adana Faculty of Medicine, Adana City Education and Research Hospital, Adana, Turkey** (T Cil MD); **Centre Hospitalier de l'Université de Montréal, Montreal, QC, Canada** (F Aubin MD); **Bristol Myers Squibb, Princeton, NJ, USA** (E Cela PhD, T Chen PhD, M Lei PhD, L Jin MD, S I Blum PhD); **Istituto Oncologico Veneto IOV-IRCCS, Padua, Italy** (S Lonardi MD)

Correspondence to: Prof Thierry Andre, Sorbonne Université, Hôpital Saint Antoine, Assistance Publique Hôpitaux de Paris, 75012 Paris, France **thierry.andre@aphp.fr**

Research in context

Evidence before this study

On Nov 19, 2024, we searched PubMed for articles published between Jan 1, 2019, and Nov 19, 2024, with no language restrictions, reporting primary results from phase 3 trials of immune checkpoint inhibitors for the treatment of microsatellite instability-high or mismatch repair-deficient metastatic colorectal cancer using the search string "((MSI-H[Title/Abstract]) OR (dMMR[Title/Abstract])) AND (colorectal cancer[Title/Abstract]) AND ((metastatic[Title/Abstract]) OR (advanced[Title/Abstract])) AND ((phase 3[Title/Abstract]) OR (phase III[Title/Abstract]))", filtering for clinical trial articles only. We also searched the American Society of Clinical Oncology and the European Society for Medical Oncology websites for abstracts published between Jan 1, 2021, and Nov 19, 2024, with no language restrictions, using the search terms "MSI-H", "dMMR", and "metastatic colorectal cancer", identifying primary results of phase 3 clinical trial abstracts published since 2021. These searches identified KEYNOTE-177 as the only phase 3 clinical trial investigating an immune checkpoint inhibitor in microsatellite instability-high or mismatch repair-deficient metastatic colorectal cancer with published primary results before CheckMate 8HW. In the KEYNOTE-177 study, significant improvement in progression-free survival with pembrolizumab monotherapy versus chemotherapy was observed in previously untreated patients with microsatellite instability-high or mismatch repair-deficient metastatic colorectal cancer. Based on these results, clinical practice guidelines for metastatic colorectal cancer have since recommended pembrolizumab as the standard of care for microsatellite instability-high or mismatch repair-deficient metastatic colorectal cancer in the first-line setting. At the first prespecified interim analysis from CheckMate 8HW, nivolumab plus ipilimumab showed superior progression-free survival compared with chemotherapy in the first-line setting, with manageable safety.

Added value of this study

To our knowledge, CheckMate 8HW is the first phase 3, randomised trial to investigate the use of dual-agent immunotherapy versus single-agent immunotherapy in patients with microsatellite instability-high or mismatch repair-deficient metastatic colorectal cancer. Nivolumab plus ipilimumab showed a clinically meaningful and significant progression-free survival benefit and a significantly higher response rate compared with nivolumab monotherapy in this patient population. Progression-free survival benefit was also observed across evaluated subgroups. A higher incidence of treatment-related adverse events was observed with nivolumab plus ipilimumab versus nivolumab; the safety profile of nivolumab plus ipilimumab was manageable and no new safety signals were reported.

Implications of all the available evidence

The results from the CheckMate 8HW trial establish the efficacy and manageable safety of dual-agent immunotherapy versus single-agent immunotherapy for the treatment of microsatellite instability-high or mismatch repair-deficient metastatic colorectal cancer. Coupled with the data from nivolumab plus ipilimumab versus chemotherapy in the first-line setting, these results support nivolumab plus ipilimumab as a potential new standard of care in patients with microsatellite instability-high or mismatch repair-deficient metastatic colorectal cancer.

Introduction

Tumours with high microsatellite instability, mismatch repair deficiency, or both are found in approximately 4–7% of patients with metastatic colorectal cancer and are associated with poor outcomes with chemotherapy with or without targeted therapies.[1–4] The treatment of microsatellite instability-high or mismatch repair-deficient metastatic colorectal cancer has advanced substantially with the introduction of immune checkpoint inhibitors.[5–7] Pembrolizumab, a PD-1 inhibitor, showed improved progression-free survival versus chemotherapy in patients with microsatellite instability-high or mismatch repair-deficient metastatic colorectal cancer in the first-line setting.[8] However, there remains an unmet need in this population, as 45 (29%) of 153 patients treated with pembrolizumab had progressive disease as best overall response, and 48% and 42% were progression-free and alive at 2 years and 3 years of follow-up, respectively.[8,9]

In the phase 2, non-randomised CheckMate 142 study, nivolumab, a PD-1 inhibitor, plus ipilimumab, a cytotoxic T-lymphocyte antigen 4 inhibitor, showed promising efficacy, including long-term survival benefit, and manageable safety in previously treated and untreated immunotherapy-naive patients with microsatellite instability-high or mismatch repair-deficient metastatic colorectal cancer.[10–13] Indirect comparisons of non-randomised cohorts within CheckMate 142 suggested better outcomes with nivolumab plus ipilimumab than with nivolumab monotherapy and underscored the importance of determining the potential benefit of dual-agent immunotherapy versus single-agent immunotherapy in the treatment of microsatellite instability-high or mismatch repair-deficient metastatic colorectal cancer in a randomised setting.[11,14] The ongoing, phase 3, international, randomised CheckMate 8HW trial was designed to evaluate nivolumab plus ipilimumab compared with nivolumab monotherapy or chemotherapy with or without targeted therapy in patients with microsatellite instability-high or mismatch repair-deficient metastatic colorectal cancer. At the previous prespecified interim analysis (median follow-up 31·5 months), nivolumab plus ipilimumab showed superior progression-free survival compared with chemotherapy in the first-line setting (median progression-free survival not reached

[95% CI 38·4 to not estimable] *vs* 5·9 months [4·4–7·8]; hazard ratio [HR] 0·21 [95% CI 0·13–0·35]; p<0·0001 with the use of a two-sided stratified log-rank test), meeting one of the dual primary endpoints of this study.[15,16] Progression-free survival at 24 months was 72% with nivolumab plus ipilimumab versus 14% with chemotherapy. In this study, we report results from the prespecified interim analysis of the other dual primary endpoint of progression-free survival for nivolumab plus ipilimumab compared with nivolumab monotherapy across all lines of therapy. Additionally, we present longer follow-up results of progression-free survival for nivolumab plus ipilimumab compared with chemotherapy in the first-line setting.

Methods

Study design and participants

This randomised, open-label, international, phase 3 trial was done at 128 hospitals and cancer centres in 23 countries (appendix p 2). Patients were enrolled in the trial if they were aged at least 18 years and had received a diagnosis of unresectable or metastatic colorectal cancer and high microsatellite instability or mismatch repair deficiency (or both) status per local testing. Sex data were self-reported by the trial participants, with male or female options provided for participant's sex at birth. Patients were enrolled across different lines of therapy and study enrolment occurred in two sequential parts: part 1 was open to patients across all treatment lines and part 2 was open to patients with no previous treatment for metastatic disease after completion of part 1. Patients who had received neoadjuvant or adjuvant therapy (or both) and had disease recurrence within 6 months after completion of therapy were considered to have received one previous treatment. Patients who had received triplet therapy combining fluorouracil, oxaliplatin, and irinotecan were considered to have received two previous treatments. Presence of measurable disease per Response Evaluation Criteria in Solid Tumours (RECIST) version 1.1 and an Eastern Cooperative Oncology Group (ECOG) performance status of 0 or 1 were required for eligibility. ECOG performance status is assessed on a five-point scale, with 0 indicating no performance restrictions and higher scores indicating greater disability. Patients who had received previous immunotherapies (anti-PD-1, anti-PD-L1 or anti-PD-L2, anti-cytotoxic T-lymphocyte antigen-4, or any other antibody or drug targeting T-cell co-stimulation or checkpoint pathways) were excluded. Additional eligibility criteria are provided in the appendix (p 8).

The trial was done in accordance with the Good Clinical Practice guidelines of the International Council for Harmonisation of Technical Requirements for Pharmaceuticals for Human Use and the principles of the Declaration of Helsinki. The protocol (appendix pp 25–410) was approved by the institutional review board or independent ethics committee at each site. All patients provided written informed consent. An independent data monitoring committee evaluated the trial interim analysis results. This study is registered with ClinicalTrials.gov, NCT04008030.

Randomisation and masking

Patients with zero or one previous treatment for metastatic disease were randomly assigned (2:2:1) to one of three treatment groups: nivolumab plus ipilimumab, nivolumab alone, or investigator's choice of chemotherapy with or without targeted therapies. Patients with two or more previous treatments for unresectable or metastatic disease were randomly assigned (1:1) to either nivolumab plus ipilimumab or nivolumab alone. In part 1 enrolment (across all treatment lines), patients were stratified at randomisation by tumour location (right *vs* left) and the number of previous treatments for unresectable or metastatic disease (0 *vs* 1 *vs* ≥2). In part 2 enrolment (the first-line setting), randomisation was stratified by tumour sidedness (right *vs* left) only.

See Online for appendix

The treatment allocation list was developed by the study sponsor (Bristol Myers Squibb). Patients were centrally randomly assigned by use of an interactive response technology system with a permuted blocks method (block size of 5). CheckMate 8HW was an open-label trial, and the treatments administered to the patients remained unmasked.

Procedures

For patients receiving nivolumab plus ipilimumab, nivolumab 240 mg in combination with ipilimumab 1 mg/kg of bodyweight was administered intravenously every 3 weeks for the first 12 weeks (up to four total doses of ipilimumab), followed by nivolumab 480 mg monotherapy every 4 weeks. Those randomly assigned to receive nivolumab monotherapy received nivolumab 240 mg intravenously every 2 weeks for the first 12 weeks, followed by nivolumab 480 mg monotherapy every 4 weeks. Investigator's choice of chemotherapy with or without targeted therapies was administered per the dosing and administration schedule specified in the protocol. Optional crossover to nivolumab (240 mg every 2 weeks for the first 12 weeks, followed by 480 mg every 4 weeks) plus ipilimumab (1 mg/kg of bodyweight every 6 weeks) was permitted for patients with disease progression in the chemotherapy group (as determined by blinded independent central review). Treatments were discontinued at disease progression, withdrawal of consent, or unacceptable toxicity. Patients in the nivolumab or nivolumab plus ipilimumab groups received study treatment for a maximum of 2 years (including patients who crossed over to nivolumab plus ipilimumab). The 2-year treatment duration was based on data suggesting that 2 years of PD-1 checkpoint inhibition might be sufficient for long-term benefit,[8,13,14] alongside data showing a shorter treatment duration in patients with advanced non-small-cell lung cancer was associated with an increased risk of progression.[17]

Additional details on the trial design and schedule of assessments are provided in the appendix (p 8) and protocol (pp 25–410), respectively.

Outcomes

This study had independent dual primary endpoints of progression-free survival by blinded independent central review per RECIST version 1.1 in patients with centrally confirmed microsatellite instability-high or mismatch repair-deficient unresectable or metastatic colorectal cancer. One primary endpoint was progression-free survival for nivolumab plus ipilimumab versus chemotherapy in the first-line setting and the other was progression-free survival for nivolumab plus ipilimumab versus nivolumab across all lines of therapy. The primary efficacy population for this trial was patients with microsatellite instability-high or mismatch repair-deficient status confirmed centrally either by the immunohistochemistry assay mismatch repair immunohistochemistry panel pharmDx (Dako Omnis; codes GE079, GE087, GE085, GE086; Agilent, Santa Clara, CA, USA) or PCR-based Idylla MSI Test (Biocartis; Mechelen, Belgium). Key secondary endpoints included overall survival, progression-free survival as determined by investigator assessment, progression-free survival as determined by blinded independent central review in all patients who underwent random assignment, and objective response (a confirmed best overall complete or partial response according to RECIST version 1.1) as determined by investigator and blinded independent central review. The trial is ongoing to assess secondary endpoints according to the hierarchical testing plan (appendix p 9), including progression-free survival for nivolumab plus ipilimumab versus nivolumab in the first-line setting, which did not meet the prespecified statistical criteria for significance at this interim analysis, as determined by the data monitoring committee, and therefore remains masked until its final analysis. Key exploratory endpoints included safety and health-related quality of life, measured with the European Organisation for Research and Treatment of Cancer (EORTC) Core Quality of Life questionnaire (QLQ-C30). The prespecified within-group minimally important mean changes from baseline in EORTC QLQ-C30 score for Global Health Status were 10 for improvement and –10 for deterioration. Health-related quality of life analyses were done in patients with centrally confirmed microsatellite instability-high or mismatch repair-deficient status who received at least one administration of study treatment and had patient-reported outcome data. Further details on central microsatellite instability-high or mismatch repair-deficient confirmation, PD-L1 testing procedures, safety, and health-related quality of life analyses are provided in the appendix (pp 9–10).

Adverse events, including those related to study treatment, were assessed in all patients who received at least one dose of study treatment. These events were graded according to National Cancer Institute Common Terminology Criteria for Adverse Events version 5.0.

Statistical analysis

To control the overall type I error at 0·05, the α was initially split between the dual primary endpoints, with 0·044 for progression-free survival in nivolumab plus ipilimumab versus chemotherapy (first line) and 0·006 for nivolumab plus ipilimumab versus nivolumab (across all lines). Progression-free survival with first-line nivolumab plus ipilimumab versus chemotherapy met the prespecified statistical criteria at the Oct 12, 2023 data cutoff.[16] Therefore, an α of 0·024 was passed to the nivolumab plus ipilimumab versus nivolumab (across all lines) analysis, for an overall α of 0·03. With a group sequential design for progression-free survival endpoints, the α distribution between interim analysis and final analysis was determined based on the actual number of progression-free survival events per blinded independent central review observed at the interim analysis and the target number of events at final analysis, using Lan-DeMets α spending function with O'Brien-Fleming boundaries. Under these assumptions, approximately 564 patients (randomly assigned in a 1:1 ratio to the nivolumab plus ipilimumab and nivolumab groups) were expected to provide approximately 96·8% power for an assumed HR of 0·635, with an overall type I error of 0·03 (two-sided), after approximately 319 events were observed. The interim analysis was planned 60 months after random assignment of the first patient in the study, and approximately 240 events were projected to have occurred at that time (information fraction 75%).

If progression-free survival per blinded independent central review in nivolumab plus ipilimumab versus nivolumab (all lines) met the prespecified statistical significance, an α of 0·006 would be passed to the secondary endpoint, objective response rate per blinded independent central review, for the same groups. Based on an assumed 18% difference in objective response rate between nivolumab plus ipilimumab and nivolumab in all lines, 564 randomly assigned patients with centrally confirmed microsatellite instability-high or mismatch repair-deficient status were expected to provide approximately 93% power with an overall type I error of 0·006 (two-sided) to show a statistically significant difference between these groups.

Progression-free survival per blinded independent central review was compared between nivolumab plus ipilimumab and nivolumab via a two-sided stratified log-rank test. Median progression-free survival with 95% CIs and rates at fixed timepoints were estimated using Kaplan–Meier methods. The HRs and associated 95% CIs were estimated using a stratified Cox proportional hazards model using the randomised group as a single covariate. Objective response rates were

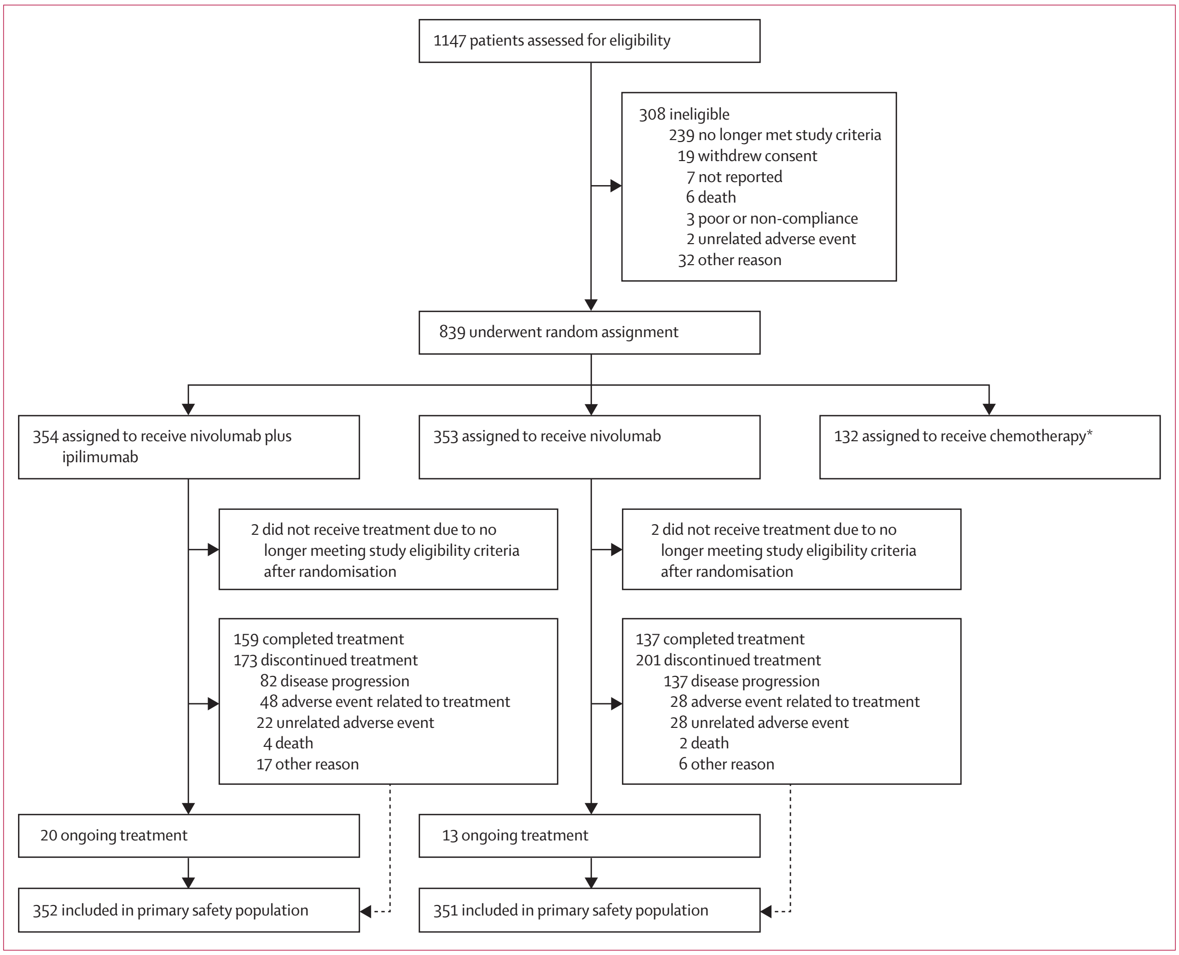

Figure 1: **Trial profile**
*Part 1 of enrolment was open for longer than part 2; therefore there is a different sample size for this analysis than previously reported.[16]

compared using a two-sided stratified Cochran–Mantel–Haenszel test. Difference in objective response rates with a 95% CI was calculated. Additional details on the statistical methods and testing procedures are in the appendix (p 9). Statistical analyses were done using SAS version 9.04.

Role of the funding source

Bristol Myers Squibb (the sponsor of the study), in collaboration with Ono Pharmaceutical, funded the trial, provided the trial agents, and collaborated with the academic authors on the trial design and on the collection, analysis, and interpretation of the data. Medical writing support, including development of the first draft of the manuscript under the guidance of the authors, was funded by the sponsor.

Results

Between Aug 16, 2019, and April 10, 2023, 839 patients with unresectable or metastatic microsatellite instability-high or mismatch repair-deficient colorectal cancer by local testing were randomly assigned to receive nivolumab plus ipilimumab (354 patients), nivolumab (353 patients), or chemotherapy (132 patients) across all lines of therapy (figure 1). 202 (57%) of 354 patients in the nivolumab plus ipilimumab group, 201 (57%) of 353 patients in the nivolumab group, and 101 (77%) of 132 patients in the chemotherapy group were previously untreated. Baseline patient demographics and disease characteristics were similar between the two treatment groups (table 1). 296 (84%) of 354 patients in the nivolumab plus ipilimumab group and 286 (81%) of 353 patients in the nivolumab group were centrally confirmed to have microsatellite instability-high or mismatch repair-deficient status (table 1; appendix p 11) and constituted the primary efficacy population. 125 (18%) of 707 randomly assigned patients did not have central confirmation of their microsatellite instability-high or mismatch repair-deficient status due to microsatellite stable status, mismatch repair proficiency of their tumours, or other reasons (table 1). Patient demographics and baseline characteristics of patients

	Nivolumab plus ipilimumab group (n=354)	Nivolumab group (n=353)
**Age, years**		
Median (IQR)	62 (52–70)	63 (51–70)
<65	199 (56%)	193 (55%)
≥65	155 (44%)	160 (45%)
**Sex**		
Male	162 (46%)	190 (54%)
Female	192 (54%)	163 (46%)
**Race**		
White	311 (88%)	305 (86%)
Asian	27 (8%)	36 (10%)
Black or African American	4 (1%)	7 (2%)
Other	12 (3%)	5 (1%)
**Geographical region**		
USA, Canada, or Europe	251 (71%)	246 (70%)
Asia	26 (7%)	33 (9%)
Rest of world	77 (22%)	74 (21%)
**Eastern Cooperative Oncology Group performance status**		
0	192 (54%)	183 (52%)
1	162 (46%)	170 (48%)
**Disease stage at initial diagnosis***		
Stage I	2 (1%)	4 (1%)
Stage II	65 (18%)	61 (17%)
Stage III	133 (38%)	129 (37%)
Stage IV	152 (43%)	158 (45%)
Not reported	2 (1%)	1 (<1%)
**Disease stage at study entry**		
Stage IVA	129 (36%)	132 (37%)
Stage IVB	110 (31%)	98 (28%)
Stage IVC	114 (32%)	122 (35%)
Not reported	1 (<1%)	1 (<1%)
**Number of previous lines of therapy†**		
0	202 (57%)	201 (57%)
1	67 (19%)	67 (19%)
≥2	85 (24%)	85 (24%)
**Tumour sidedness‡**		
Right	241 (68%)	240 (68%)
Left	113 (32%)	113 (32%)
**Sites of metastases by blinded independent central review§**		
Liver	140 (40%)	149 (42%)
Lung	85 (24%)	99 (28%)
Peritoneum	143 (40%)	126 (36%)
**Centrally confirmed microsatellite instability-high or mismatch repair-deficient status**		
Yes	296 (84%)	286 (81%)
No	58 (16%)	67 (19%)
MSS and pMMR	41 (12%)	40 (11%)
MSS or pMMR¶	8 (2%)	10 (3%)
Other‖	9 (3%)	17 (5%)
(Continued from previous column)		
**PD-L1 expression****		
<1%	255 (72%)	264 (75%)
≥1%	74 (21%)	63 (18%)
***BRAF, KRAS,* and *NRAS* mutation status**		
BRAF, *KRAS*, and *NRAS* all wild type	83 (23%)	103 (29%)
BRAF mutant	106 (30%)	85 (24%)
KRAS or *NRAS* mutant	83 (23%)	89 (25%)
BRAF and *KRAS* or *NRAS* mutant	9 (3%)	2 (1%)
Unknown	73 (21%)	74 (21%)
**Clinical history of Lynch syndrome**		
Yes	48 (14%)	49 (14%)
No	217 (61%)	207 (59%)
Unknown	86 (24%)	91 (26%)
Not reported	3 (1%)	6 (2%)
**Previous systemic therapies**		
Any previous systemic therapy	218 (62%)	213 (60%)
**Previous systemic therapy setting**		
Neoadjuvant	18/218 (8%)	16/213 (8%)
Adjuvant	116/218 (53%)	94/213 (44%)
Metastatic	124/218 (57%)	137/213 (64%)

(Table 1 continues in next column)

Data are n (%) or n/N (%), unless otherwise indicated. CRF=case report form. IRT=interactive response technology. MSS=microsatellite stable. pMMR=mismatch repair proficient. *Disease stage not reported in two patients in the nivolumab plus ipilimumab group and one patient in the nivolumab group. †Numbers here are using IRT criteria—numbers from the CRF were 193 (55%) untreated at metastatic stage, 82 (23%) one line, and 78 (22%) two or more lines in the nivolumab plus ipilimumab group, and 184 (52%) untreated at metastatic stage, 86 (24%) one line, and 83 (24%) two or more lines in the nivolumab group. ‡Numbers here are using IRT criteria—numbers of patients from the CRF were 244 (69%) patients with right tumour sidedness and 110 (31%) with left tumour sidedness in the nivolumab plus ipilimumab group, and 244 (69%) patients with right tumour sidedness and 109 (31%) with left tumour sidedness in the nivolumab group. §Metastatic sites not reported in three patients in the nivolumab plus ipilimumab group and two patients in the nivolumab group; patients could have more than one site of metastasis. ¶In the nivolumab plus ipilimumab group, six patients had pMMR tumours and were not tested for microsatellite instability; two patients had MSS tumours and could not be evaluated for mismatch repair status. In the nivolumab group, seven patients had pMMR tumours and were not tested for microsatellite instability; three patients had MSS tumours and could not be evaluated for mismatch-repair status. ‖26 patients were not evaluable or not tested for both microsatellite instability and mismatch repair status (nine patients in the nivolumab plus ipilimumab group and 17 patients in the nivolumab group). **Tumour cell PD-L1 expression indeterminate, not evaluable, or not available for 25 patients in the nivolumab plus ipilimumab group and 26 patients in the nivolumab group.

***Table 1:* Patient demographics and disease characteristics at baseline in all randomly assigned patients**

within the primary efficacy population were similar to those from the all randomised population (table 1; appendix p 11).

At the data cutoff on Aug 28, 2024, median follow-up (from randomisation to data cutoff) was 47·0 months (IQR 38·4–53·2). 703 patients received treatment: 352 in the nivolumab plus ipilimumab group and 351 in the nivolumab group. Among treated patients, 296 (159 [45%]

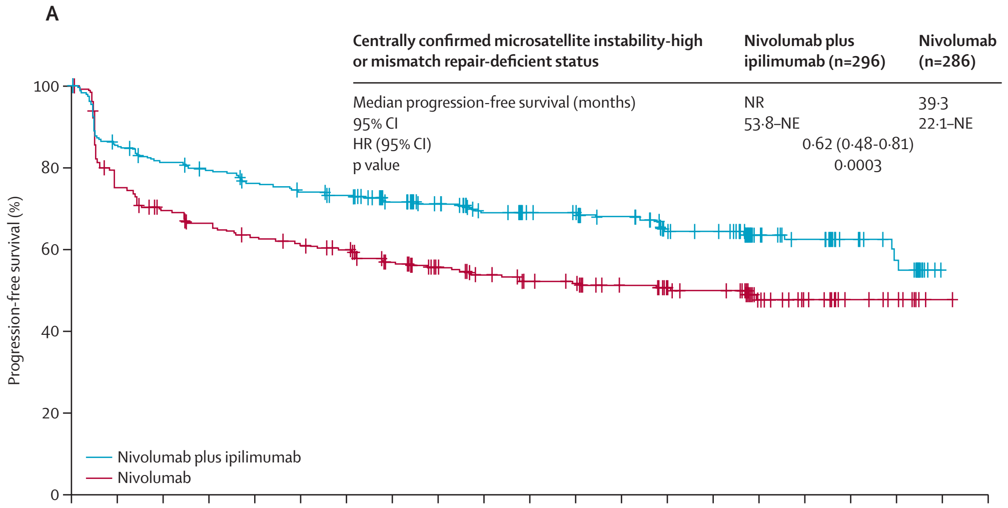

Number at risk (number censored)																					
Nivolumab plus ipilimumab	296	248	234	225	214	207	200	180	164	146	136	134	121	102	100	61	54	29	23	0	0
	(0)	(5)	(7)	(10)	(12)	(13)	(18)	(34)	(49)	(62)	(72)	(74)	(85)	(98)	(100)	(138)	(144)	(169)	(173)	(195)	(195)
Nivolumab	286	210	191	179	169	164	158	141	124	109	98	95	81	72	69	39	31	15	12	1	0
	(0)	(5)	(9)	(11)	(12)	(13)	(16)	(25)	(39)	(50)	(58)	(60)	(73)	(81)	(83)	(111)	(119)	(135)	(138)	(149)	(150)

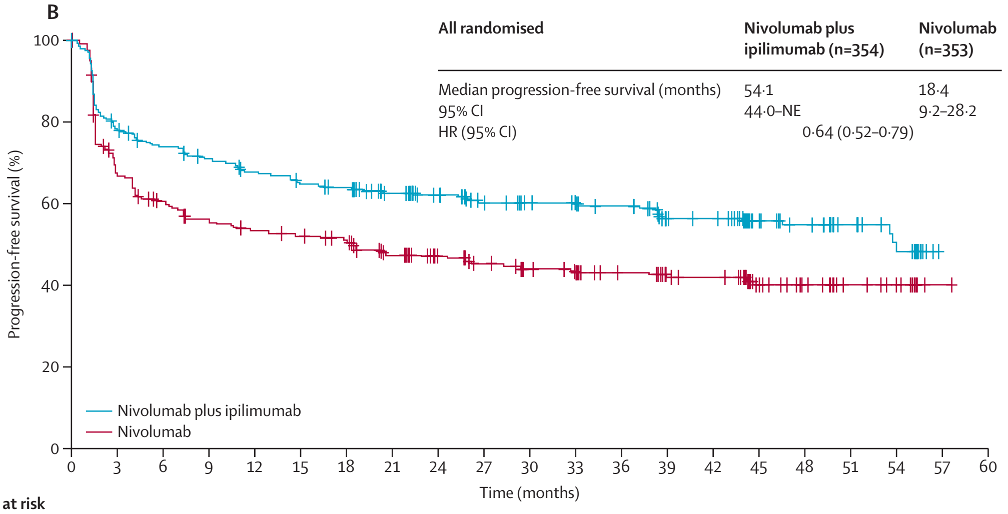

Number at risk (number censored)																					
Nivolumab plus ipilimumab	354	271	253	240	227	216	208	187	170	152	141	139	126	106	104	63	56	29	23	0	0
	(0)	(7)	(10)	(13)	(15)	(16)	(21)	(38)	(54)	(67)	(78)	(80)	(91)	(105)	(107)	(147)	(153)	(180)	(184)	(206)	(206)
Nivolumab	353	230	202	187	177	172	166	146	128	112	101	98	84	75	72	39	31	15	12	1	0
	(0)	(7)	(11)	(13)	(14)	(15)	(18)	(28)	(43)	(54)	(62)	(64)	(77)	(85)	(87)	(118)	(126)	(142)	(145)	(156)	(157)

***Figure 2:* Progression-free survival by blinded independent central review with nivolumab plus ipilimumab versus nivolumab**

(A) Patients with centrally confirmed microsatellite instability-high or mismatch repair-deficient status. The boundary for statistical significance was p<0·0095. (B) All patients who underwent randomisation. For both patient populations, stratified Cox proportional hazard model by tumour sidedness (left *vs* right) and previous lines of therapy (0 *vs* 1 *vs* ≥2) per interactive response technology was used. Vertical dashes indicate censored data. HR=hazard ratio. NE=not estimable. NR=not reached.

of 352 patients in the nivolumab plus ipilimumab group and 137 [39%] of 351 patients in the nivolumab group) completed 2 years of treatment. 173 (49%) of 352 patients in the nivolumab plus ipilimumab group and 201 (57%) of 351 patients in the nivolumab group discontinued treatment. Treatment discontinuation due to disease progression was reported in 82 (23%) of 352 patients in the nivolumab plus ipilimumab group and 137 (39%) of 351 patients in the nivolumab group (figure 1). A trial profile of treated patients with centrally confirmed microsatellite instability-high or mismatch repair-deficient status is shown in the appendix (p 17).

	Number of events/number of patients		Unstratified HR for progression-free survival (95% CI)
	Nivolumab plus ipilimumab	Nivolumab	
**Age (years)**			
<65	51/164	70/157	0·60 (0·42–0·87)
≥65	50/132	66/129	0·66 (0·46–0·96)
**Sex**			
Male	47/135	76/149	0·60 (0·42–0·87)
Female	54/161	60/137	0·67 (0·47–0·98)
**Region**			
USA, Canada, and Europe	76/209	105/206	0·63 (0·47–0·84)
Asia	4/23	10/29	0·40 (0·13–1·29)
Rest of world	21/64	21/51	0·73 (0·40–1·33)
**Eastern Cooperative Oncology Group performance status**			
0	51/164	60/149	0·69 (0·47–1·00)
1	50/132	76/137	0·60 (0·42–0·85)
**Tumour sidedness**			
Left	25/80	32/72	0·62 (0·37–1·04)
Right	76/216	104/214	0·64 (0·47–0·86)
**Liver metastases***			
Yes	37/104	51/106	0·68 (0·44–1·03)
No	64/190	85/178	0·60 (0·43–0·83)
**Peritoneal metastases***			
Yes	44/126	53/100	0·55 (0·37–0·82)
No	57/168	83/184	0·67 (0·48–0·94)
**Tumour cell PD-L1 expression**			
≥1%	21/72	21/61	0·77 (0·42–1·42)
<1%	74/214	111/213	0·57 (0·43–0·77)
***BRAF/KRAS/NRAS* mutation status**			
BRAF, *KRAS*, and *NRAS* all wild-type	26/75	37/81	0·64 (0·39–1·06)
BRAF mutant	34/98	41/81	0·62 (0·39–0·97)
KRAS or *NRAS* mutant	22/57	31/68	0·76 (0·44–1·31)
Unknown	16/59	26/55	0·48 (0·26–0·91)
**Lynch syndrome**			
Yes	19/43	18/40	0·90 (0·47–1·72)
No	54/172	78/162	0·56 (0·40–0·80)
Unknown	28/78	36/78	0·71 (0·43–1·16)
**Overall**	**101/296**	**136/286**	**0·63 (0·49–0·82)**

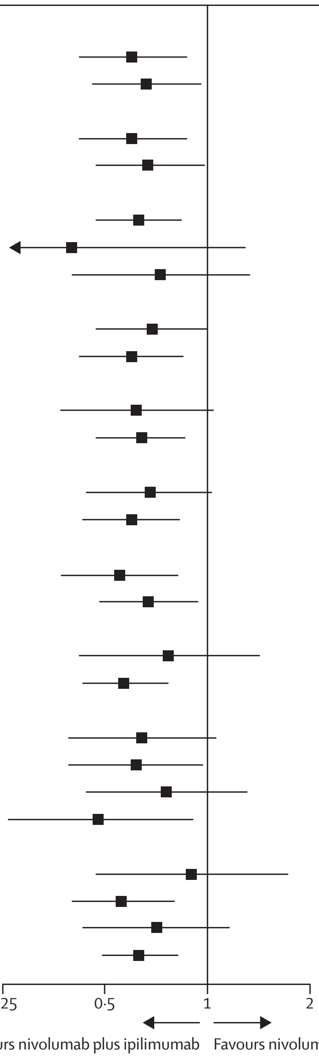

Figure 3: **Progression-free survival by blinded independent central review in key subgroups of patients with centrally confirmed microsatellite instability-high or mismatch repair-deficient status**
Unstratified HRs are reported for patient subgroup analyses. Based on Kaplan–Meier estimates; rates not computed for subgroups with less than ten patients per treatment group. HR=hazard ratio. *Metastatic sites were determined by blinded independent central review and were not reported in three patients in the nivolumab plus ipilimumab group and two patients in the nivolumab group; patients could have more than one site of metastasis.

Nivolumab plus ipilimumab treatment showed significant and clinically meaningful improvement in progression-free survival versus nivolumab in patients with centrally confirmed microsatellite instability-high or mismatch repair-deficient metastatic colorectal cancer (HR 0·62, 95% CI 0·48 to 0·81; p=0·0003 by two-sided stratified log-rank test; figure 2). Median progression-free survival was not reached with nivolumab plus ipilimumab (95% CI 53·8 to not estimable) and was 39·3 months with nivolumab (22·1 to not estimable; figure 2). The estimated proportions of patients in the nivolumab plus ipilimumab group who were alive and progression-free at 12 months, 24 months, and 36 months were 76% (95% CI 71 to 80), 71% (95% CI 65 to 76), and 68% (95% CI 62 to 73), respectively; corresponding rates with nivolumab were 63% (95% CI 57 to 68), 56% (95% CI 49 to 61), and 51% (95% CI 45 to 57). In prespecified subgroup analyses, progression-free survival generally favoured nivolumab plus ipilimumab versus nivolumab (figure 3).

	Nivolumab plus ipilimumab group (n=296)	Nivolumab group (n=286)	p value
Objective response rate (95% CI)	209 (71%) [65–76]	165 (58%) [52–64]	0·0011
Best overall response			··
Complete response	90 (30%)	80 (28%)	··
Partial response	119 (40%)	85 (30%)	··
Stable disease	40 (14%)	53 (19%)	··
Progressive disease	30 (10%)	54 (19%)	··
Unevaluable	17 (6%)	14 (5%)	··
Median time to response, months (IQR)	2·8 (1·4–4·2)	2·8 (1·5–4·2)	··
Median duration of response, months (95% CI)	NR (NE)	NR (NE)	··

Data are n (%), unless otherwise indicated. NE=not estimable. NR=not reached.

***Table 2:* Best overall response by blinded review in patients with centrally confirmed microsatellite instability-high or mismatch repair-deficient status**

	Nivolumab plus ipilimumab group (n=352)		Nivolumab group (n=351)	
	Any grade	Grade 3 or 4	Any grade	Grade 3 or 4
Any treatment-related adverse event	285 (81%)	78 (22%)	249 (71%)	50 (14%)
Treatment-related serious adverse event	65 (18%)	55 (16%)	29 (8%)	24 (7%)
Treatment-related adverse event leading to discontinuation of any drug in the regimen	48 (14%)	33 (9%)	21 (6%)	14 (4%)
Treatment-related deaths*	2 (1%)	··	1 (<1%)	··
Treatment-related adverse events reported in ≥5% of patients in either group				
Pruritus	91 (26%)	0	63 (18%)	0
Diarrhoea	71 (20%)	3 (1%)	59 (17%)	2 (1%)
Hypothyroidism	61 (17%)	2 (1%)	31 (9%)	0
Asthenia	58 (16%)	2 (1%)	44 (13%)	2 (1%)
Fatigue	42 (12%)	1 (<1%)	35 (10%)	1 (<1%)
Hyperthyroidism	40 (11%)	0	16 (5%)	0
Arthralgia	38 (11%)	1 (<1%)	23 (7%)	0
Adrenal insufficiency	34 (10%)	8 (2%)	12 (3%)	3 (1%)
Rash	34 (10%)	3 (1%)	29 (8%)	1 (<1%)
Increased alanine aminotransferase	31 (9%)	6 (2%)	21 (6%)	3 (1%)
Increased aspartate aminotransferase	27 (8%)	3 (1%)	17 (5%)	3 (1%)
Hypophysitis	20 (6%)	10 (3%)	3 (1%)	3 (1%)
Increased lipase	18 (5%)	6 (2%)	14 (4%)	7 (2%)
Nausea	18 (5%)	0	18 (5%)	0

Data are n (%). All events between the first dose of treatment and 30 days after the last dose of treatment were reported. *Treatment-related adverse events leading to death were reported regardless of timeframe.

***Table 3:* Treatment-related adverse events in all treated patients who received at least one dose of the assigned treatment**

Improvements in progression-free survival with nivolumab plus ipilimumab versus nivolumab were consistent among all randomly assigned patients (HR 0·64, 95% CI 0·52 to 0·79); median progression-free survival in all randomly assigned patients was 54·1 months (95% CI 44·0 to not estimable) with nivolumab plus ipilimumab and 18·4 months (9·2 to 28·2) with nivolumab (figure 2). The findings from the analysis of progression-free survival according to investigator assessment in patients with centrally confirmed microsatellite instability-high or mismatch repair-deficient status were consistent with the findings from the blinded independent central review (median not reached [95% CI 54·1 months to not estimable] and 38·1 months [95% CI 27·2 months to not estimable], respectively; HR 0·62, 95% CI 0·48 to 0·80; appendix p 18); concordance between progression-free survival by blinded independent central review and by investigator assessment was 88% in the nivolumab plus ipilimumab group and 89% in the nivolumab group when comparing total numbers of events (disease progression or death) and censored cases.

The objective response rate by blinded independent central review was significantly higher with nivolumab plus ipilimumab versus nivolumab in patients with centrally confirmed microsatellite instability-high or mismatch repair-deficient status (209 [71%] of 296 patients; 95% CI 65–76 and 165 [58%] of 286 patients; 52–64, respectively; p=0·0011; table 2). Complete responses were reported in 90 (30%) of 296 patients in the nivolumab plus ipilimumab group and 80 (28%) of 286 patients in the nivolumab group; progressive disease as best response was reported in 30 (10%) patients and 54 (19%) patients, respectively (table 2). Median duration of response was not reached for either of the treatment groups; median time to response was similar across both groups (2·8 months [IQR 1·4–4·2] for nivolumab plus ipilimumab and 2·8 months [1·5–4·2] for nivolumab; table 2; appendix p 20). Among response-evaluable patients (patients with target lesion assessment at baseline and at least one on-treatment tumour assessment) with centrally confirmed microsatellite instability-high or mismatch repair-deficient status, 217 (77%) of 281 in the nivolumab plus ipilimumab group and 169 (62%) of 273 in the nivolumab group had at least a 30% reduction in the sum of diameters of target lesions from baseline (appendix p 21). In prespecified subgroup analyses, objective response rate was higher with nivolumab plus ipilimumab versus nivolumab (appendix p 22). Objective responses by blinded independent central review in all randomly assigned patients were consistent with those from patients with centrally confirmed microsatellite instability-high or mismatch repair-deficient status (appendix p 14).

At this updated analysis (Aug 28, 2024 data cutoff and minimum follow-up of 16·7 months), nivolumab plus ipilimumab continued to show progression-free survival benefit versus chemotherapy in patients with centrally confirmed microsatellite instability-high or mismatch repair-deficient status in the first-line setting with longer follow-up. Median progression-free survival was 54·1 months (95% CI 54·1 to not estimable) in the nivolumab plus ipilimumab group and 5·9 months (4·4 to 7·8) in the chemotherapy group (HR 0·21, 95% CI 0·14 to 0·31; appendix p 23). The 24-month progression-free survival rate with longer follow-up was 74% (95% CI 67 to 80) with nivolumab plus ipilimumab and 11% (4 to 21) with chemotherapy; 36-month progression-free survival rates were 69% (61 to 76) and 11% (4 to 21), respectively.

Among all treated patients, the overall median duration of treatment was 20·5 months (IQR 3·8–23·6) in the nivolumab plus ipilimumab group (20·5 months [3·8–23·6] for nivolumab and 2·1 months [2·1–2·1] for ipilimumab) and 16·4 months (3·7–23·5) in the nivolumab group (appendix p 13). The median number of ipilimumab doses received by those in the nivolumab plus ipilimumab group was four (IQR 4–4); 288 (82%) of 352 patients received all four doses of ipilimumab.

Adverse events of any grade and any cause occurred in 349 (99%) of 352 patients in the nivolumab plus ipilimumab group and in 336 (96%) of 351 patients in the nivolumab group; adverse events of grade 3 or 4 occurred in 168 (48%) patients and 151 (43%) patients, respectively (appendix p 15). Treatment-related adverse events of any grade occurred in 285 (81%) of 352 patients receiving nivolumab plus ipilimumab and in 249 (71%) of 351 patients receiving nivolumab; grade 3 or 4 treatment-related adverse events occurred in 78 (22%) and 50 (14%) patients, respectively (table 3). The most common treatment-related adverse event was pruritus, which occurred in 91 (26%) of 352 patients receiving nivolumab plus ipilimumab and 63 (18%) of 351 patients receiving nivolumab alone. Treatment-related adverse events which led to treatment discontinuation of any drug in the regimen occurred in 48 (14%) of 352 patients and 21 (6%) of 351 patients (table 3). The most common grade 3 or 4 immune-mediated adverse events in the nivolumab plus ipilimumab and nivolumab groups were diarrhoea or colitis (12 [3%] of 352 patients and eight [2%] of 351 patients, respectively), hypophysitis (11 [3%] of 352 patients and four [1%] of 351 patients, respectively), and adrenal insufficiency (ten [3%] of 352 patients and three [<1%] of 351 patients, respectively). A summary of immune-mediated adverse events is in the appendix (p 16).

Among the 703 patients who received at least one dose of treatment, 252 deaths were reported (103 [29%] of 352 patients in the nivolumab plus ipilimumab group and 149 [42%] of 351 patients in the nivolumab group). The most common cause of death in both groups was disease progression. There were three treatment-related deaths: one event of myocarditis and pneumonitis each in the nivolumab plus ipilimumab group and one pneumonitis event in the nivolumab group (table 3).

Improvements from baseline in health-related quality of life were observed in both the nivolumab plus ipilimumab and nivolumab groups as measured by the EORTC QLQ-C30 Global Health Status subscale (appendix p 24). Mean change from baseline scores were positive in both treatment groups, with the nivolumab plus ipilimumab group reaching the prespecified threshold for meaningful change starting at week 21 and remaining at or near the within-group minimally important change from baseline of 10 at most timepoints starting from week 21.

Discussion

To our knowledge, CheckMate 8HW is the first randomised, phase 3 trial in microsatellite instability-high or mismatch repair-deficient metastatic colorectal cancer to report superior progression-free survival and objective response rate with the dual-immune checkpoint inhibitors, nivolumab plus ipilimumab compared with single-agent immunotherapy (nivolumab). Nivolumab plus ipilimumab showed significant and clinically meaningful improvement in progression-free survival versus nivolumab monotherapy across all lines of therapy in patients with centrally confirmed microsatellite instability-high or mismatch repair-deficient metastatic colorectal cancer. In our study, progression-free survival rates were higher with nivolumab plus ipilimumab versus nivolumab. In prespecified subgroup analyses, progression-free survival favoured nivolumab plus ipilimumab versus nivolumab. In patients with centrally confirmed microsatellite instability-high or mismatch repair-deficient status, progression-free survival by blinded independent central review was robust and supported by investigator assessment, with a high degree of concordance between these assessments. Furthermore, progression-free survival by blinded independent review in patients with centrally confirmed microsatellite instability-high or mismatch repair-deficient status was also consistent with data for all randomly assigned patients, where microsatellite instability-high or mismatch repair-deficient status was determined by local tests. The progression-free survival outcomes with nivolumab plus ipilimumab in all randomly assigned patients in this study are also consistent with data from patients receiving nivolumab plus ipilimumab in the phase 2 CheckMate 142 trial, in which local confirmation of microsatellite instability-high or mismatch repair-deficient status was done.[11,14] In the first 3 months after random assignment, there was a more pronounced decline in progression-free survival in the all randomised group compared with patients who had centrally confirmed microsatellite instability-high or mismatch repair-deficient status. This observation could be attributed to the presence of misdiagnosed patients by local testing among the all randomised population. 99 (14%) of 707 patients had microsatellite stable or mismatch repair-proficient status according to central testing. Given that this patient group

has been observed to be more resistant to immune checkpoint inhibitors,[18,19] broad adoption of validated immunohistochemistry and PCR-based (or next-generation sequencing) testing is paramount. However, analysis of the all randomised group presents an opportunity to estimate the efficacy and safety of nivolumab and ipilimumab in a real-world context in which clinicians use various tests, including those that are locally developed, to identify patients with microsatellite instability-high or mismatch repair-deficient colorectal cancer.

Previously, we reported improved progression-free survival with first-line nivolumab plus ipilimumab versus chemotherapy in patients with centrally confirmed microsatellite instability-high or mismatch repair-deficient metastatic colorectal cancer, one of the dual primary endpoints from CheckMate 8HW.[16] In the longer follow-up analyses reported here, first-line nivolumab plus ipilimumab continued to show progression-free survival benefit over chemotherapy with higher 2-year and 3-year progression-free survival rates with nivolumab plus ipilimumab.[15,16] The 2-year and 3-year landmark progression-free survival rates with nivolumab plus ipilimumab observed in CheckMate 8HW were similar in the first-line and all-lines settings.

In the CheckMate 8HW study, there was a significant and clinically meaningful improvement in objective response rate with nivolumab plus ipilimumab versus nivolumab across all lines of therapy in patients with centrally confirmed microsatellite instability-high or mismatch repair-deficient status. Furthermore, addition of ipilimumab led to reduced rates of progressive disease as best response. Objective response rates among those with locally tested high microsatellite instability or mismatch repair deficiency were similar to those from CheckMate 142.[10,13] To our knowledge, the objective response rate observed in CheckMate 8HW for nivolumab plus ipilimumab across all lines of therapy in centrally confirmed microsatellite instability-high or mismatch repair-deficient metastatic colorectal cancer is the highest reported in a randomised setting. Responses to treatment were durable in both the nivolumab plus ipilimumab and nivolumab groups, and median duration of response was not reached in both groups. The high complete response rate in this study suggests cure can be achieved in a proportion of patients, even in a metastatic setting. Future exploration of this hypothesis could include potential landmark analyses of progression-free survival and overall survival by best overall response, as well as biomarker analyses assessing correlations between radiographic response and circulating tumour DNA.

In the nivolumab group in our study, for all randomly assigned patients with local high microsatellite instability or mismatch repair deficiency assessment and mixed number of previous lines of therapy, the efficacy results were similar to those from previously untreated patients receiving pembrolizumab in KEYNOTE-177.[8,9,20] Rates of primary progressive disease were also similar to KEYNOTE-177, in which high microsatellite instability or mismatch repair deficiency was locally determined, although it is important to note that the proportion of patients with misdiagnosed microsatellite instability-high or mismatch repair-deficient status in KEYNOTE-177 is unknown. The outcomes observed across all lines in our study might be driven by previously untreated patients, due to a large proportion of patients receiving study treatment in the first-line setting; however, at the time of database lock, efficacy outcomes by line of therapy remain masked.

Safety of nivolumab plus ipilimumab was consistent with the profile observed in previously reported results comparing nivolumab plus ipilimumab with chemotherapy[16] and with the known profiles of each individual component. The incidence of drug-related adverse events was higher in the nivolumab plus ipilimumab group compared with the nivolumab group. The most frequently reported drug-related adverse events were pruritus, diarrhoea, and hypothyroidism with nivolumab plus ipilimumab and pruritus, diarrhoea, and asthenia with nivolumab. Particularly noteworthy was the higher incidence of immune-mediated endocrine adverse events in the nivolumab plus ipilimumab group compared with the nivolumab group, most of which were grade 1 and 2. However, a proportion of patients had resolution of these events with or without the need for ongoing hormone replacement therapy. Additionally, there were higher rates of drug-related adverse events leading to discontinuation with nivolumab plus ipilimumab, although patients were able to continue with nivolumab after early discontinuation of ipilimumab. Despite these differences in safety between the treatment groups, patients had improvements from baseline in health-related quality of life with nivolumab plus ipilimumab and nivolumab. The mean changes from baseline in EORTC QLQ-C30 Global Health Status scores remained close to the prespecified threshold for meaningful change with nivolumab plus ipilimumab and nivolumab at most timepoints from week 21 onward, showing the efficacy with nivolumab plus ipilimumab compared with nivolumab was not at the expense of diminished health-related quality of life.

It is important to note the limitations of this trial. First, as the trial was open label, it is conceivable that there were biases in reporting of treatment assessments; however, the high degree of concordance between blinded independent central review and the investigator assessment of progression-free survival suggests that the open-label design did not affect efficacy assessments. Second, due to the relatively shorter duration of minimum follow-up for the current interim analysis, some secondary endpoints, such as duration of response, remain immature; these will be addressed with additional follow-up. Third, patient numbers in some of the prespecified subgroups were low, limiting data interpretation. Additionally, although subgroup analyses for assessment of progression-free

survival and objective response rates by line of treatment would have been of interest, treatment lines remain masked at this time because these data are not yet mature, in line with the hierarchical testing plan. Finally, although progression-free survival is an established endpoint to assess clinical benefit with immunotherapy in microsatellite instability-high or mismatch repair-deficient metastatic colorectal cancer, overall survival data remain masked at this time and will be valuable to further contextualise these study results.

In conclusion, to our knowledge this is the first randomised phase 3 trial to investigate the use of dual-agent immunotherapy versus single-agent immunotherapy in patients with microsatellite instability-high or mismatch repair-deficient metastatic colorectal cancer, contributing to the body of evidence across multiple indications that dual-agent immunotherapy shows clinical benefit compared with single-agent immunotherapies.[21,22] The significant progression-free survival and objective response benefits reported here with nivolumab plus ipilimumab over nivolumab support use across lines of therapy in this setting. The safety of nivolumab plus ipilimumab was consistent with the established profiles of each individual drug, and no new safety concerns were identified. Taken together, these results strongly support nivolumab plus ipilimumab as a potential new standard of care in microsatellite instability-high or mismatch repair-deficient metastatic colorectal cancer.

Contributors

TA, H-JL, LHJ, TY, JL, EC, TCh, ML, LJ, SIB, and SL contributed to the concept and design of the study in collaboration with Bristol Myers Squibb. TA, EE, H-JL, LHJ, YT, EVC, RG-C, DT, GAM, MS, CdlF, MLL, TY, JL, JLMM, LD, GT, MC, EG, MIB, RJ, TCi, FA, EC, ML, and SL were involved in data collection. EC, TCh, ML, SIB, and LJ were responsible for data analysis. All authors were involved in critical data interpretation and reviewed and edited the manuscript. All authors had full access to all the data in the study and had final responsibility for the decision to submit for publication. All authors reviewed the final version of the manuscript to be submitted and agree with its content and submission. All authors vouch for the accuracy and completeness of the data and for the fidelity of the trial to the protocol.

Declaration of interests

TA reports consulting or advisory roles for Amgen, Aptitude Health, Astellas Pharma, Bristol Myers Squibb, GamaMabs Pharma, Gritstone Bio, Gilead Sciences, GlaxoSmithKline, MSD Oncology, Nordic Bioscience, Seagen, Servier, Pfizer, Pierre Fabre, Takeda-Lundbeck, Tesaro, and Transgene; receiving honoraria from Amgen, Bristol Myers Squibb, MSD Oncology, Merck Serono, GlaxoSmithKline, Pierre Fabre, Roche-Genentech, Sanofi, Seagen, Servier, and Ventana Medical Systems; a data monitoring committee role for Inspirna; being President of the ARCAD foundation (Aide à la recherché en cancérologie digestive), a member of the ACCENT Collaborative Group and Gercor; and travel or accommodation expenses from Bristol Myers Squibb, MSD Oncology, Takeda, and Servier. EE reports consulting or advisory roles for Amgen, Bayer, Boehringer Ingelheim, Bristol Myers Squibb, Cureteq, Jannsen, Merck Serono, Merck Sharp & Dohme, Novartis, Pfizer, Pierre Fabre, Repare Therapeutics, RIN Institute, Roche-Genentech, Sanofi, Seagen, Servier, and Takeda; receiving honoraria from Amgen, Bayer, Bristol Myers Squibb, Boehringer Ingelheim, Cureteq, Janssen, Lilly, Medscape, Merck Sharp & Dohme, Merck Serono, Novartis, Pfizer, Pierre Fabre, Repare Therapeutics, RIN Institute, Roche-Genentech, Sanofi, Seagen, Servier, and Takeda; research funding to their institution from AbbVie, Amgen, Array BioPharma, AstraZeneca, Bayer, BeiGene, Bioncotech, BioNTech, Boehringer Ingelheim, Boehringer Ingelheim (Spain), Bristol Myers Squibb, Celgene, Daiichi Sankyo, Debiopharm Group, Gercor, HalioDx, Hutchison MediPharma, Iovance Biotherapeutics, Janssen-Cilag, Janssen R&D, MedImmune, Menarini, Merck, Merck Sharp & Dohme, Merus NV, Mirati, Novartis, Nouscom, PharmaMar, Pfizer, PledPharma, RedX Pharma, Pierre Fabre, Roche-Genentech, Sanofi, Scandion Oncology, Seagen, Servier, Sotio, Taiho, and WntResearch; and travel or accommodation expenses from Amgen, Bayer, Boehringer Ingelheim, Bristol Myers Squibb, Cureteq, Jannsen, Lilly, Medscape, Merck Sharp & Dohme, Merck Serono, Novartis, Pfizer, Pierre Fabre, Repare Therapeutics, RIN Institute, Roche-Genentech, Sanofi, Servier, Seagen, and Takeda. H-JL reports consulting or advisory roles for Bayer, Bristol Myers Squibb, Fulgent Genetics, GlaxoSmithKline, Merck Serono, Roche-Genentech, and 3T BioSciences; receiving honoraria from Bayer, Boehringer Ingelheim, Fulgent Genetics, G1 Therapeutics, Isofol Medical, Jazz Pharmaceuticals, Merck Serono, Oncocyte, and Roche-Genentech; and travel or accommodation expenses from Bayer, Bristol Myers Squibb, and Merck Serono. LHJ reports receiving research grants to their institution from 2cureX, Bristol Myers Squibb, Roche-Genentech, Incyte, Merck Sharp & Dohme, and Pfizer. YT reports consulting or advisory roles for Bristol Myers Squibb, Merck Serono, Pierre Fabre, and Servier; and travel or accommodation expenses from Merck Serono, MSD Oncology, Pierre Fabre, and Servier. EVC reports consulting or advisory roles for AbbVie, Agenus, Amgen, ALX Oncology, Arcus Biosciences, Astellas Pharma, AstraZeneca, Bayer, BeiGene, Boehringer Ingelheim, Bristol Myers Squibb, Daiichi Sankyo, Debiopharm Group, ElmediX, Eisai, GlaxoSmithKline, Hookipa Biotech, Incyte, Ipsen, Lilly, Merck KGaA, Merck Sharp & Dohme, Mirati, Nordic Group, Novartis, Pfizer-BioNTech, Pierre Fabre, Roche-Genentech, Seagen, Servier, Simcere, Taiho, Takeda, and Terumo. RG-C reports receiving honoraria from Advanced Accelerator Applications-Novartis, Astellas Pharma, AstraZeneca, Bayer, Boehringer Ingelheim, Bristol Myers Squibb, Esteve, Gilead Sciences, Hutchmed, Ipsen, Lilly, Merck, Merck Sharp & Dohme, Midatech Pharma, Mirati, Pfizer, Pharmamar, Roche-Genentech, Sanofi, Servier, and Takeda; receiving funding to their institution from Bristol Myers Squibb, Merck Sharp & Dohme, and Pfizer; and travel or accommodation expenses from Advanced Accelerator Applications-Novartis, Esteve, Ipsen, Merck Serono, and Merck Sharp & Dohme. DT reports consulting or advisory roles for AstraZeneca, Bristol Myers Squibb, Merck Sharp & Dohme, Novartis, Pierre Fabre, Sanofi, Servier, and Takeda; receiving honoraria from Amgen, AstraZeneca, Bristol Myers Squibb, Merck Serono, Merck Sharp & Dohme, Pierre Fabre, Roche-Genentech, Sanofi, Servier-Pfizer, and Takeda; research funding to their institution from BTG, Merck Sharp & Dohme, Pierre Fabre, Roche-Genentech, and Takeda; and travel or accommodation expenses from Amgen, Bristol Myers Squibb, MSD Oncology, Pierre Fabre, Roche-Genentech, and Servier. GAM reports consulting or advisory roles for Amgen, Bristol Myers Squibb, Merck KGaA, Merck Sharp & Dohme, Pfizer, and Roche-Genentech; receiving speakers fees from Amgen, Bayer, Bristol Myers Squibb, Grupo Biotoscana, Merck KGaA, Merck Sharp & Dohme, Pfizer, Roche-Genentech, and Servier; research funding from Amgen, AstraZeneca, Bristol Myers Squibb, Merck Sharp & Dohme, and Roche-Genentech; patents, royalties, and intellectual properties with Bristol Myers Squibb and Merck Sharp & Dohme; and travel or accommodation expenses from Amgen, Grupo Biotoscana, Merck KGaA, Pfizer, and Servier. MS reports travel or accommodation expenses from Bristol Myers Squibb and Pfizer; and research funding from AbbVie, Amgen, Astellas Pharma, AstraZeneca, BeiGene, Bioven, Bristol Myers Squibb, Clovis Oncology, Daiichi Sankyo Europe, Eisai, Five Prime Therapeutics, Gilead Sciences, GlaxoSmithKline, Lilly, Merck Sharp & Dohme, Mylan, Novartis, Pfizer-EMD Serono, PharmaMar, Regeneron, Roche, Tesaro, Samsung Healthcare, and Sanofi-Regeneron. CdlF reports consulting or advisory roles for Amgen, Astellas Pharma, Bristol Myers Squibb, Daiichi Sankyo, Eisai, Ipsen, Lilly, MSD Oncology, Pierre Fabre, Roche-Genentech, Servier, and Takeda; research funding to their institution from Merck Sharp & Dohme, Pierre Fabre, and Servier; and travel or accommodation expenses from Amgen, MSD Oncology, Pierre Fabre, Roche-Genentech, and Servier. TY reports consulting or advisory roles for Sumitomo Corporation; receiving honoraria from Chugai Pharma, Merck KGaA, Merck Sharp & Dohme, and Takeda; and research funding to their institution from Amgen, Bristol Myers Squibb

Japan, Chugai Pharma, Daiichi Sankyo, Eisai, Falco Biosystems, Medical & Biological Laboratories Co, Merck Sharp & Dohme, Merus, Molecular Health, Ono Pharmaceutical, Pfizer, Roche-Genentech, Sanofi, Sysmex, Taiho, and Takeda. GT reports consulting or advisory roles for AstraZeneca, Bristol Myers Squibb, Merck KGaA, and Servier. MC reports consulting or advisory roles for Bristol Myers Squibb and Numab; and research funding to their institution from Bristol Myers Squibb, Merck Sharp and Dohme, and Roche-Genentech. EG reports consultancy roles for AstraZeneca, Bristol Myers Squibb, Daiichi Sankyo, and Merck Sharp and Dohme; and travel or accommodation expenses from Daiichi Sankyo. MIB reports consulting or advisory roles for Astellas, AstraZeneca, Bristol Myers Squibb, Daiichi Sankyo, Ipsen, Merck Sharp & Dohme, and Pfizer; receiving honoraria from Astellas, AstraZeneca, Bristol Myers Squibb, Daiichi Sankyo, Ipsen, Merck Sharp & Dohme, Pfizer, and Servier; and travel or accommodation expenses from Astellas and Daiichi Sankyo. RJ reports consulting or advisory roles for AstraZeneca, Merck Sharp & Dohme, and Roche-Genentech; receiving honoraria from Bristol Myers Squibb, Gilead Sciences, Ipsen, Merck Sharp & Dohme, Pfizer, Pfizer-EMD Serono, and Roche-Genentech; receiving speakers fees from Gilead Sciences; and travel or accommodation expenses from Novartis. FA reports consulting or advisory roles for Amgen, Bristol Myers Squibb, Merck KGaA, Pfizer, and Taiho; receiving honoraria from Amgen, Bristol Myers Squibb, Merck KGaA, Pfizer, and Taiho; and research funding to their institution from Bristol Myers Squibb, GlaxoSmithKline, Merck KGaA, and Novartis. EC, TCh, ML, LJ, and SIB report employment with Bristol Myers Squibb and ownership of stock in Bristol Myers Squibb. SL reports consulting or advisory roles for Amgen, Astellas Pharma, AstraZeneca, Bayer, Bristol Myers Squibb, Daiichi Sankyo, GlaxoSmithKline, Incyte, Lilly, Merck Serono, Merck Sharp & Dohme, Servier, Rottapharm Biotech, and Takeda; receiving speakers fees from Amgen, Bristol Myers Squibb, GlaxoSmithKline, Incyte, Lilly, Merck Serono, MSD Oncology, Pierre Fabre, Roche-Genentech, and Servier; and research funding from Amgen and Merck Serono (to self), and from AstraZeneca, Bayer, Bristol Myers Squibb, Lilly, and Roche-Genentech (to their institution). All other authors declare no competing interests.

Data sharing
Bristol Myers Squibb will honour legitimate requests for clinical trial data from qualified researchers who submit an in-scope proposal approved by the independent review committee. Before data are released, the researcher(s) must sign a data sharing agreement, after which de-identified and anonymised datasets can be accessed within a secure portal. The Bristol Myers Squibb policy on data sharing can be found at https://www.bms.com/researchers-andpartners/independent-research/data-sharing-request-process.html.

Acknowledgments
We thank the patients and their families for making this trial possible; the investigators, research staff, and the clinical trial team at Bristol Myers Squibb (Princeton, NJ, USA) and Ono Pharmaceutical (Osaka, Japan) for CheckMate 8HW trial support; Janice Kaps-Trotter (Bristol Myers Squibb) for contributions as the global trial manager; Carine Cabilla for clinical operations support; the Precision Medicine team (Bristol Myers Squibb) for central mismatch repair and microsatellite instability testing; Ruslan Novosiadly for diagnostics support; Agilent Technologies for collaborative development of the mismatch repair immunohistochemistry panel pharmDx (Dako Omnis) assay (Santa Clara, CA, USA) and Biocartis for collaborative development of the Idylla MSI test (Mechelen, Belgium); and Christopher Spencer of Parexel for medical writing assistance, funded by Bristol Myers Squibb.

References

1 Venderbosch S, Nagtegaal ID, Maughan TS, et al. Mismatch repair status and BRAF mutation status in metastatic colorectal cancer patients: a pooled analysis of the CAIRO, CAIRO2, COIN, and FOCUS studies. *Clin Cancer Res* 2014; **20:** 5322–30.

2 Gutierrez C, Ogino S, Meyerhardt JA, Iorgulescu JB. The prevalence and prognosis of microsatellite instability-high/mismatch repair-deficient colorectal adenocarcinomas in the United States. *JCO Precis Oncol* 2023; **7:** e2200179.

3 Innocenti F, Ou FS, Qu X, et al. Mutational analysis of patients with colorectal cancer in CALGB/SWOG 80405 identifies new roles of microsatellite instability and tumor mutational burden for patient outcome. *J Clin Oncol* 2019; **37:** 1217–27.

4 Tougeron D, Sueur B, Zaanan A, et al. Prognosis and chemosensitivity of deficient MMR phenotype in patients with metastatic colorectal cancer: an AGEO retrospective multicenter study. *Int J Cancer* 2020; **147:** 285–96.

5 Cervantes A, Adam R, Roselló S, et al. Metastatic colorectal cancer: ESMO Clinical Practice Guideline for diagnosis, treatment and follow-up. *Ann Oncol* 2023; **34:** 10–32.

6 Morris VK, Kennedy EB, Baxter NN, et al. Treatment of metastatic colorectal cancer: ASCO guideline. *J Clin Oncol* 2023; **41:** 678–700.

7 Le DT, Uram JN, Wang H, et al. PD-1 blockade in tumors with mismatch-repair deficiency. *N Engl J Med* 2015; **372:** 2509–20.

8 André T, Shiu KK, Kim TW, et al. Pembrolizumab in microsatellite-instability-high advanced colorectal cancer. *N Engl J Med* 2020; **383:** 2207–18.

9 Diaz LA Jr, Shiu KK, Kim TW, et al. Pembrolizumab versus chemotherapy for microsatellite instability-high or mismatch repair-deficient metastatic colorectal cancer (KEYNOTE-177): final analysis of a randomised, open-label, phase 3 study. *Lancet Oncol* 2022; **23:** 659–70.

10 Lenz HJ, Van Cutsem E, Luisa Limon M, et al. First-line nivolumab plus low-dose ipilimumab for microsatellite instability-high/mismatch repair-deficient metastatic colorectal cancer: the phase 2 CheckMate 142 study. *J Clin Oncol* 2022; **40:** 161–70.

11 Overman MJ, Lonardi S, Wong KYM, et al. Durable clinical benefit with nivolumab plus ipilimumab in DNA mismatch repair-deficient/microsatellite instability-high metastatic colorectal cancer. *J Clin Oncol* 2018; **36:** 773–79.

12 Overman MJ, McDermott R, Leach JL, et al. Nivolumab in patients with metastatic DNA mismatch repair-deficient or microsatellite instability-high colorectal cancer (CheckMate 142): an open-label, multicentre, phase 2 study. *Lancet Oncol* 2017; **18:** 1182–91.

13 André T, Lonardi S, Wong KYM, et al. Nivolumab plus low-dose ipilimumab in previously treated patients with microsatellite instability-high/mismatch repair-deficient metastatic colorectal cancer: 4-year follow-up from CheckMate 142. *Ann Oncol* 2022; **33:** 1052–60.

14 Overman MJ, Lenz H-J, Andre T, et al. Nivolumab (NIVO) ± ipilimumab (IPI) in patients (pts) with microsatellite instability-high/mismatch repair-deficient (MSI-H/dMMR) metastatic colorectal cancer (mCRC): five-year follow-up from CheckMate 142. *J Clin Oncol* 2022; **40:** 3510.

15 Andre T, Elez E, Van Cutsem E, et al. Nivolumab (NIVO) plus ipilimumab (IPI) vs chemotherapy (chemo) as first-line (1L) treatment for microsatellite instability-high/mismatch repair-deficient (MSI-H/dMMR) metastatic colorectal cancer (mCRC): first results of the CheckMate 8HW study. *J Clin Oncol* 2024; **42:** LBA768

16 Andre T, Elez E, Van Cutsem E, et al. Nivolumab plus ipilimumab in microsatellite-instability-high metastatic colorectal cancer. *N Engl J Med* 2024; **391:** 2014–26.

17 Waterhouse DM, Garon EB, Chandler J, et al. Continuous versus 1-year fixed-duration nivolumab in previously treated advanced non-small-cell lung cancer: CheckMate 153. *J Clin Oncol* 2020; **38:** 3863–73.

18 Cohen R, Hain E, Buhard O, et al. Association of primary resistance to immune checkpoint inhibitors in metastatic colorectal cancer with misdiagnosis of microsatellite instability or mismatch repair deficiency status. *JAMA Oncol* 2019; **5:** 551–55.

19 André T, Lonardi S, Lenz HJ, et al. Nivolumab (NIVO) plus ipilimumab (IPI) *vs* chemotherapy (chemo) as first-line (1L) treatment for microsatellite instability-high/mismatch repair-deficient (MSI-H/dMMR) metastatic colorectal cancer (mCRC): subgroup efficacy and expanded safety analyses from CheckMate 8HW. *Ann Oncol* 2024; **35:** S451–52.

20 André T, Shiu K-K, Kim TW, et al. Pembrolizumab versus chemotherapy in microsatellite instability-high or mismatch repair-deficient metastatic colorectal cancer: 5-year follow-up from the randomized phase 3 KEYNOTE-177 study. *Ann Oncol* 2024; published online Dec 2. https://doi.org/10.1016/j.annonc.2024.11.012.

21 Larkin J, Chiarion-Sileni V, Gonzalez R, et al. Five-year survival with combined nivolumab and ipilimumab in advanced melanoma. *N Engl J Med* 2019; **381:** 1535–46.

22 Brahmer JR, Lee JS, Ciuleanu TE, et al. Five-year survival outcomes with nivolumab plus ipilimumab versus chemotherapy as first-line treatment for metastatic non-small-cell lung cancer in CheckMate 227. *J Clin Oncol* 2023; **41:** 1200–12.
