# Supplementary material for: Dual-immunotherapy triumphs: redefining deficient mismatch repair or high microsatellite instability metastatic colorectal cancer first-line treatment
Source: Signal Transduct Target Ther. 2025 Jul 15;10:234. doi: 10.1038/s41392-025-02322-8 (PMC12264035; doi:10.1038/s41392-025-02322-8)
Supplement: Supplementary file 3 — REF3 [file 41392_2025_2322_MOESM3_ESM.pdf]

ORIGINAL ARTICLE

# Nivolumab plus Ipilimumab in Microsatellite-Instability–High Metastatic Colorectal Cancer

T. Andre, E. Elez, E. Van Cutsem, L.H. Jensen, J. Bennouna, G. Mendez, M. Schenker, C. de la Fouchardiere, M.L. Limon, T. Yoshino, J. Li, H.-J. Lenz, J.L. Manzano Mozo, G. Tortora, R. Garcia-Carbonero, L. Dahan, M. Chalabi, R. Joshi, E. Goekkurt, M.I. Braghiroli, T. Cil, E. Cela, T. Chen, M. Lei, M. Dixon, S. Abdullaev, and S. Lonardi, for the CheckMate 8HW Investigators*

## ABSTRACT

### BACKGROUND
Patients with microsatellite-instability–high (MSI-H) or mismatch-repair–deficient (dMMR) metastatic colorectal cancer have poor outcomes with standard chemotherapy with or without targeted therapies. Nivolumab plus ipilimumab has shown clinical benefit in nonrandomized studies of MSI-H or dMMR metastatic colorectal cancer.

### METHODS
In this phase 3 open-label trial, we randomly assigned patients with unresectable or metastatic colorectal cancer and MSI-H or dMMR status according to local testing to receive, in a 2:2:1 ratio, nivolumab plus ipilimumab, nivolumab alone, or chemotherapy with or without targeted therapies. The dual primary end points, assessed in patients with centrally confirmed MSI-H or dMMR status, were progression-free survival with nivolumab plus ipilimumab as compared with chemotherapy as first-line therapy and progression-free survival with nivolumab plus ipilimumab as compared with nivolumab alone in patients regardless of previous systemic treatment for metastatic disease. At this prespecified interim analysis, the first primary end point (involving nivolumab plus ipilimumab vs. chemotherapy) was assessed.

### RESULTS
A total of 303 patients who had not previously received systemic treatment for metastatic disease were randomly assigned to receive nivolumab plus ipilimumab or chemotherapy; 255 patients had centrally confirmed MSI-H or dMMR tumors. At a median follow-up of 31.5 months (range, 6.1 to 48.4), progression-free survival outcomes (the primary analysis) were significantly better with nivolumab plus ipilimumab than with chemotherapy (P<0.001 for the between-group difference in progression-free survival, calculated with the use of a two-sided stratified log-rank test); 24-month progression-free survival was 72% (95% confidence interval [CI], 64 to 79) with nivolumab plus ipilimumab as compared with 14% (95% CI, 6 to 25) with chemotherapy. At 24 months, the restricted mean survival time was 10.6 months (95% CI, 8.4 to 12.9) longer with nivolumab plus ipilimumab than with chemotherapy, a finding consistent with the primary analysis of progression-free survival. Grade 3 or 4 treatment-related adverse events occurred in 23% of the patients in the nivolumab-plus-ipilimumab group and in 48% of the patients in the chemotherapy group.

### CONCLUSIONS
Progression-free survival was longer with nivolumab plus ipilimumab than with chemotherapy among patients who had not previously received systemic treatment for MSI-H or dMMR metastatic colorectal cancer. (Funded by Bristol Myers Squibb and Ono Pharmaceutical; CheckMate 8HW ClinicalTrials.gov number, NCT04008030.)

The authors' full names, academic degrees, and affiliations are listed in the Appendix. Dr. Andre can be contacted at thierry.andre@aphp.fr or at Department of Medical Oncology, Hôpital Saint Antoine, Assistance Publique–Hôpitaux de Paris, 184 Rue du Faubourg Saint-Antoine, 75012, Paris, France.

*A list of the CheckMate 8HW Investigators is provided in the Supplementary Appendix, available at NEJM.org.

N Engl J Med 2024;391:2014-26.
DOI: 10.1056/NEJMoa2402141
Copyright © 2024 Massachusetts Medical Society.

CME



The New England Journal of Medicine is produced by NEJM Group, a division of the Massachusetts Medical Society.
Downloaded from nejm.org at Shanghai Jiao Tong University on November 29, 2024. For personal use only.
No other uses without permission. Copyright © 2024 Massachusetts Medical Society. All rights reserved.

Colorectal cancer is the third most common cancer and the second leading cause of cancer-related death worldwide, with almost one million deaths occurring annually.[1] In the United States, approximately 16% of patients with metastatic colorectal cancer survive 5 years or more after diagnosis, excluding other causes of death.[2] DNA repair defects, which can be defined as microsatellite-instability–high (MSI-H) by polymerase-chain-reaction assays or next-generation sequencing or as mismatch-repair–deficient (dMMR) by immunohistochemical assays, are reported in approximately 4 to 7% of metastatic colorectal cancer cases.[3,4] Historically, patients with MSI-H or dMMR metastatic colorectal cancer have poor outcomes when treated with standard chemotherapy with or without targeted therapies.[4] Progression-free survival was longer with the programmed death 1 (PD-1) inhibitor pembrolizumab than with chemotherapy among previously untreated patients with MSI-H or dMMR metastatic colorectal cancer in the KEYNOTE-177 trial.[5] However, 29% of the patients treated with pembrolizumab had progressive disease as the best overall response, and 48% were alive and did not have progressive disease at 2 years of follow-up.[5]

Nivolumab, a PD-1 inhibitor, and ipilimumab, a cytotoxic T-lymphocyte antigen 4 inhibitor, are immune checkpoint inhibitors with complementary mechanisms of action, which might enhance antitumor activity.[6,7] The combination of nivolumab and ipilimumab has shown favorable efficacy outcomes relative to single-agent immunotherapy in several cancer types, including MSI-H or dMMR metastatic colorectal cancer.[8-13] In the phase 2 CheckMate 142 study, nivolumab plus ipilimumab led to deep and durable responses, survival rates that are promising, and manageable safety profiles in patients with MSI-H or dMMR metastatic colorectal cancer.[10,11,13] On the basis of these findings, nivolumab plus ipilimumab has been approved in many countries for use in previously treated patients with MSI-H or dMMR metastatic colorectal cancer.[14-16]

The CheckMate 8HW trial is an ongoing, phase 3, multinational, randomized trial evaluating nivolumab plus ipilimumab as compared with nivolumab alone or chemotherapy in patients with MSI-H or dMMR metastatic colorectal cancer. Here, we report the results from the prespecified interim analysis of first-line nivolumab plus ipilimumab as compared with chemotherapy.

## Methods

### Patients

Patients were eligible to enroll in the trial if they were 18 years of age or older and had received a diagnosis of unresectable or metastatic colorectal cancer and MSI-H or dMMR status according to local testing; patients who had received various previous lines of therapy could be eligible. Patients had to have measurable disease according to the Response Evaluation Criteria in Solid Tumors (RECIST), version 1.1, and an Eastern Cooperative Oncology Group performance-status score of 0 or 1 (on a scale from 0 to 5, with higher scores indicating greater disability). Patients who had previously received treatment with an anti–PD-1, anti–programmed death ligand 1 or 2 (PD-L1 or PD-L2), anti–cytotoxic T-lymphocyte antigen 4, or other antibody or drug targeting T-cell costimulation or checkpoint pathways were excluded. Additional eligibility criteria are provided in the Supplementary Appendix, available with the full text of this article at NEJM.org.

*A Quick Take is available at NEJM.org*

### Trial Design and Treatments

The CheckMate 8HW trial is a phase 3, multinational, open-label, randomized trial. Eligible patients underwent randomization with stratification according to tumor location (right or left) and the number of previously received systemic treatments for unresectable or metastatic disease (zero, one, or at least two). Patients with zero or one treatment for metastatic disease were randomly assigned, in a 2:2:1 ratio, to receive nivolumab plus ipilimumab, nivolumab alone, or the investigator's choice of chemotherapy with or without targeted therapies (mFOLFOX6 [folinic acid, fluorouracil, and oxaliplatin] or FOLFIRI [folinic acid, fluorouracil, and irinotecan] with or without bevacizumab or cetuximab). Patients who had previously received two or more treatments for unresectable or metastatic disease were randomly assigned, in a 1:1 ratio, to receive nivolumab plus ipilimumab or nivolumab alone.

The patients in the nivolumab-plus-ipilimumab group received the combination of nivolumab at a dose of 240 mg and ipilimumab at a dose of 1 mg per kilogram of body weight, administered intravenously, every 3 weeks for the first 12 weeks, followed by nivolumab at a dose of 480 mg, as monotherapy, every 4 weeks. The patients in the nivolumab group received nivolumab as mono-



The New England Journal of Medicine is produced by NEJM Group, a division of the Massachusetts Medical Society.
Downloaded from nejm.org at Shanghai Jiao Tong University on November 29, 2024. For personal use only.
No other uses without permission. Copyright © 2024 Massachusetts Medical Society. All rights reserved.

therapy at a dose of 240 mg, administered intravenously, every 2 weeks for the first 12 weeks, followed by nivolumab at a dose of 480 mg every 4 weeks. The patients in the chemotherapy group received the investigator's choice of chemotherapy with or without targeted therapies, administered according to the dosing and administration schedule specified in the protocol, available at NEJM.org. The patients in the chemotherapy group were permitted to receive nivolumab plus ipilimumab if disease progression, documented by blinded review, occurred; the regimen for the crossover group was nivolumab at a dose of 240 mg every 2 weeks for the first 12 weeks plus ipilimumab at a dose of 1 mg per kilogram every 6 weeks, followed by nivolumab at a dose of 480 mg every 4 weeks plus ipilimumab at a dose of 1 mg per kilogram every 6 weeks. Treatments in all the groups continued to be administered until disease progression or unacceptable toxic effects occurred or consent was withdrawn or for a maximum of 2 years in the nivolumab-plus-ipilimumab and nivolumab groups and in patients who crossed over to nivolumab plus ipilimumab. Additional details on the trial design are provided in the Supplementary Appendix, including in Figure S1.

#### END POINTS AND ASSESSMENTS

The dual primary end points, assessed in patients with centrally confirmed MSI-H or dMMR unresectable or metastatic colorectal cancer, were progression-free survival as determined by blinded review according to RECIST, version 1.1, with nivolumab plus ipilimumab as compared with chemotherapy in patients who had not previously received systemic treatment for metastatic disease and with nivolumab plus ipilimumab as compared with nivolumab alone in patients regardless of previous systemic treatment for metastatic disease. The primary efficacy population for this trial comprised patients with centrally confirmed MSI-H or dMMR status as measured by either the immunohistochemical assay MMR IHC [mismatch-repair immunohistochemical] panel pharmDx (Dako Omnis; codes GE079, GE087, GE085, GE086; Agilent) or the polymerase-chain-reaction–based Idylla MSI [microsatellite instability] test (Biocartis NV). Key secondary end points included overall survival, progression-free survival as determined by investigator assessment, progression-free survival as determined by blinded review in all patients who underwent randomization, and objective response (a confirmed best overall complete or partial response according to RECIST, version 1.1) as determined by blinded review. Key exploratory end points included safety and health-related quality of life, as measured by the European Organization for Research and Treatment of Cancer quality-of-life core questionnaire. For the Global Health Status subscale, the prespecified between-group minimally important difference in least-squares means was 5 and within-group minimally important changes from baseline in least-squares means were 6 for improvement and −6 for deterioration. Additional details of the trial design, central MSI-H or dMMR confirmation, safety, and health-related quality of life are provided in the Supplementary Appendix.

The trial is ongoing to assess the other primary end point of progression-free survival in patients receiving nivolumab plus ipilimumab as compared with nivolumab alone regardless of previous systemic treatment for metastatic disease, as well as certain secondary end points according to the hierarchical testing plan, including overall survival. These data are not yet mature.

#### TRIAL OVERSIGHT

The trial was conducted in accordance with the Good Clinical Practice guidelines of the International Council for Harmonisation of Technical Requirements for Pharmaceuticals for Human Use and the principles of the Declaration of Helsinki. The protocol was approved by the institutional review board or the independent ethics committee at each site. All the patients provided written informed consent. An independent data monitoring committee evaluated the results from the interim analysis.

Bristol Myers Squibb (which oversaw the trial), in collaboration with Ono Pharmaceutical, funded the trial, provided the trial agents, and collaborated with the academic authors on the trial design and on the collection, analysis, and interpretation of the data. All the authors vouch for the accuracy and completeness of the data and for the fidelity of the trial to the protocol. The authors had access to the trial data, participated in the development or review of the manuscript, and provided final approval to submit the manuscript for publication. Medical writing support, including the development of the first draft of the manuscript under the guidance of the authors, was funded by Bristol Myers Squibb.



The New England Journal of Medicine is produced by NEJM Group, a division of the Massachusetts Medical Society.
Downloaded from nejm.org at Shanghai Jiao Tong University on November 29, 2024. For personal use only.
No other uses without permission. Copyright © 2024 Massachusetts Medical Society. All rights reserved.

#### STATISTICAL ANALYSIS

We planned to randomly assign approximately 230 patients with centrally confirmed MSI-H or dMMR status to receive first-line nivolumab plus ipilimumab or chemotherapy in a 2:1 ratio. An estimated 125 instances of disease progression or death would provide almost 99% power to detect an average hazard ratio of 0.55 with an overall type I error of 0.044 (two-sided). An interim analysis was planned when approximately 106 events had occurred (information fraction, 85%). The alpha was distributed over the interim and final analyses on the basis of the actual number of events observed at the interim analysis and the target number of events at the final analysis (125), with the use of the Lan–DeMets alpha spending function with O'Brien–Fleming boundaries. Statistical analyses were performed with the use of SAS software, version 9.04 (SAS Institute).

Progression-free survival, assessed by blinded review among patients with centrally confirmed MSI-H or dMMR metastatic colorectal cancer who had not previously received systemic treatment for metastatic disease, was compared between the nivolumab-plus-ipilimumab group and the chemotherapy group with the use of a two-sided stratified log-rank test. If the proportional hazards assumption held, hazard ratios and associated 95% confidence intervals were to be estimated with the use of a stratified Cox proportional hazards model with the exact method of handling ties. If the proportional hazards assumption did not hold, progression-free survival was to be further examined with the use of analytic methods and descriptive additional analyses, which would be conducted with the restricted mean survival time (area under the curve of progression-free survival up to a specific time point) for each treatment group. The hierarchical testing strategy specified that certain secondary end points were not tested because the other primary end point (progression-free survival of nivolumab plus ipilimumab vs. nivolumab alone among patients regardless of previous systemic treatment for metastatic disease) was not tested at this interim analysis. Additional details on the statistical methods and testing procedures are summarized in the Statistical Testing Procedures section in the Supplementary Appendix and in the trial protocol.

## RESULTS

#### PATIENTS

From August 2019 through April 2023, eligible patients were enrolled at 121 sites in 23 countries. A total of 303 patients with MSI-H or dMMR status according to local testing who had not received systemic treatment for unresectable or metastatic colorectal cancer were randomly assigned to receive nivolumab plus ipilimumab (202 patients) or chemotherapy (101 patients) (Fig. 1). Baseline patient demographics and disease characteristics were well balanced between the two groups (Table 1). Black patients were underrepresented in the patient population (Table S1). Of the 303 patients who underwent randomization, 171 patients in the nivolumab-plus-ipilimumab group (85%) and 84 patients in the chemotherapy group (83%) had centrally confirmed MSI-H or dMMR status (Table 1 and Table S2) and constituted the primary efficacy population. The disposition and baseline characteristics of the patients with centrally confirmed MSI-H or dMMR status were well balanced between the groups and were similar to those of the total population of patients who underwent randomization (Table 1 and Fig. 1 and Table S2 and Fig. S2).

At the data-cutoff date, October 12, 2023, with a median follow-up (the time from randomization to the data-cutoff date) of 31.5 months (range, 6.1 to 48.4), a total of 96 of the 200 patients (48%) in the nivolumab-plus-ipilimumab group who had received treatment and 82 of the 88 patients (93%) in the chemotherapy group who had received treatment discontinued treatment. In the nivolumab-plus-ipilimumab group, 62 of 200 patients (31%) completed 2 years of treatment. Treatment discontinuation due to disease progression was reported in 38 of 200 patients (19%) in the nivolumab-plus-ipilimumab group and in 61 of 88 patients (69%) in the chemotherapy group (Fig. 1).

#### EFFICACY

Among patients with centrally confirmed MSI-H or dMMR status, nivolumab plus ipilimumab was associated with a progression-free survival benefit (the primary analysis) that was superior to that with chemotherapy (P<0.001 for the between-group difference in progression-free survival, calculated with the use of a two-sided stratified



The New England Journal of Medicine is produced by NEJM Group, a division of the Massachusetts Medical Society.
Downloaded from nejm.org at Shanghai Jiao Tong University on November 29, 2024. For personal use only.
No other uses without permission. Copyright © 2024 Massachusetts Medical Society. All rights reserved.

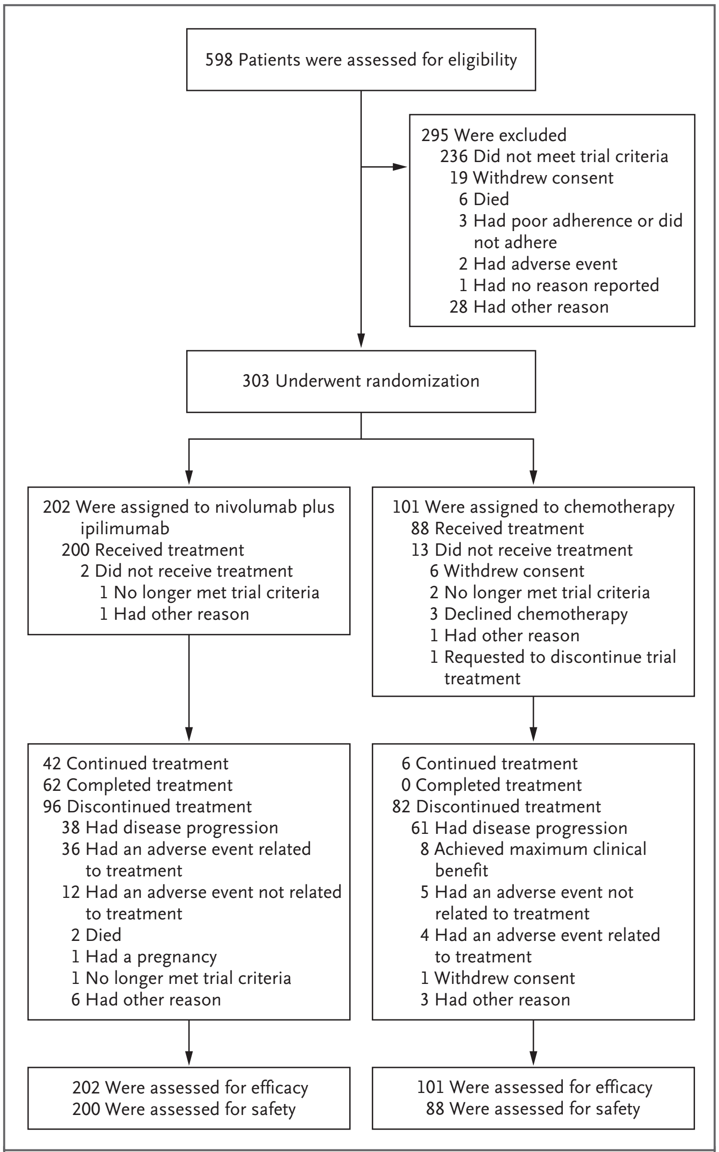


**Figure 1. Enrollment, Randomization, and Treatment.**

Not shown here are patients who were randomly assigned to receive nivolumab monotherapy and patients who had previously received at least one line of therapy for metastatic disease who were randomly assigned to receive nivolumab plus ipilimumab or chemotherapy. The median follow-up was 31.5 months.

log-rank test). According to the Kaplan–Meier curve, progression-free survival at 12 months was 79% (95% confidence interval [CI], 72 to 84) with nivolumab plus ipilimumab and 21% (95% CI, 11 to 32) with chemotherapy; 24-month progression-free survival was 72% (95% CI, 64 to 79) and 14% (95% CI, 6 to 25), respectively (Fig. 2A). Because the proportional hazards assumption was violated, additional analyses of progression-free survival were conducted to further characterize the treatment effect of first-line nivolumab plus ipilimumab as compared with chemotherapy. The estimated restricted mean survival time at 24 months was 19.2 months (95% CI, 17.9 to 20.5) with nivolumab plus ipilimumab and 8.6 months (95% CI, 6.7 to 10.4) with chemotherapy, representing a difference of 10.6 months (95% CI, 8.4 to 12.9); these results were consistent with the results of the primary analysis of progression-free survival (Fig. 2A). In prespecified subgroup analyses, progression-free survival was longer and the percentage of patients with 12-month progression-free survival was greater with nivolumab plus ipilimumab than with chemotherapy across all subgroups, including among patients with *RAS* or *BRAF* mutations; baseline liver, lung, or peritoneal metastases; and the Lynch syndrome (an inherited genetic disorder that confers a predisposition to certain cancers including colorectal cancer) (Table 2 and Table S3). Better progression-free survival outcomes with nivolumab plus ipilimumab than with chemotherapy were also observed among all patients who underwent randomization and had not previously received systemic treatment for metastatic disease (Fig. 2B).

The findings from the analysis of progression-free survival according to investigator assessment in patients with centrally confirmed MSI-H or dMMR status were consistent with the findings from the blinded review (Fig. 2A and Fig. S3); concordance between blinded review and investigator assessment with respect to events (counted as disease progression or death) and censored cases was 90% in the nivolumab-plus-ipilimumab group and 87% in the chemotherapy group.

### EXPOSURE AND SAFETY

Among all the patients who received at least one dose of treatment, the median duration of treatment was 13.5 months (range, 0 to 32.3) in the nivolumab-plus-ipilimumab group and 4.0 months (range, 0.1 to 27.5) in the chemotherapy group (Table S4). Adverse events of any grade and from any cause occurred in 99% of the patients in the nivolumab-plus-ipilimumab group and in 98% of the patients in the chemotherapy group; grade 3 or 4 adverse events occurred in 48% and in 67%, respectively (Table S5). Treatment-related adverse events of any grade occurred in 80% of the patients in the nivolumab-plus-ipilimumab



The New England Journal of Medicine is produced by NEJM Group, a division of the Massachusetts Medical Society.
Downloaded from nejm.org at Shanghai Jiao Tong University on November 29, 2024. For personal use only.
No other uses without permission. Copyright © 2024 Massachusetts Medical Society. All rights reserved.

group and in 94% of the patients in the chemotherapy group; grade 3 or 4 treatment-related adverse events occurred in 23% and in 48%, respectively (Table 3). Treatment-related adverse events leading to discontinuation of any drug in the regimen occurred in 16% of the patients in the nivolumab-plus-ipilimumab group and in 32% of the patients in the chemotherapy group (Table 3). Immune-mediated adverse events are summarized in Table S6.

Among the 288 patients who received at least one dose of treatment, 81 deaths were reported (44 of 200 patients [22%] in the nivolumab-plus-ipilimumab group and 37 of 88 patients [42%] in the chemotherapy group). Disease progression was the most common cause of death in both groups. Two treatment-related deaths were reported in the nivolumab-plus-ipilimumab group (one death from myocarditis and one from pneumonitis) (Table 3). The death from myocarditis occurred on day 54, which was 33 days after the last dose of nivolumab plus ipilimumab had been administered. The second death, from pneumonitis, occurred on day 235, while the patient was receiving subsequent therapy after disease progression had occurred during the treatment period of the trial; the patient died 102 days after the last dose of nivolumab plus ipilimumab had been administered.

### HEALTH-RELATED QUALITY OF LIFE

The changes from baseline in health-related quality of life, as measured by the European Organization for Research and Treatment of Cancer quality-of-life core questionnaire (the Global Health Status subscale), surpassed the prespecified threshold for meaningful improvements starting at week 13 in the nivolumab-plus-ipilimumab group. Changes from baseline remained stable in the chemotherapy group until week 29, when the health-related quality of life declined to a value lower than the prespecified threshold. The differences between the groups exceeded the prespecified threshold for meaningful change from week 13 onward, which indicated a health-related quality-of-life benefit with nivolumab plus ipilimumab as compared with chemotherapy (Fig. S4 and Table S7).

## DISCUSSION

In this phase 3, multinational, randomized trial, progression-free survival outcomes were significantly better with nivolumab plus ipilimumab than with chemotherapy among patients with centrally confirmed MSI-H or dMMR metastatic colorectal cancer who had not previously received systemic treatment for metastatic disease ($P<0.001$ for the between-group difference in progression-free survival, calculated with the use of a two-sided stratified log-rank test). Progression-free survival at 2 years was 72% with nivolumab plus ipilimumab and 14% with chemotherapy. Because of the violation of the proportional hazards assumption, analyses of the restricted mean survival time at 24 months were conducted to facilitate the interpretation of the progression-free survival results from the primary analysis; these additional analyses showed a difference of 10.6 months (95% CI, 8.4 to 12.9) in the restricted mean survival time in favor of nivolumab plus ipilimumab, a finding consistent with the primary analysis of progression-free survival. Furthermore, the prespecified subgroup analyses consistently favored nivolumab plus ipilimumab over chemotherapy, as indicated by numerically higher progression-free survival at 12 months in all subgroups. Nivolumab plus ipilimumab also led to better progression-free survival outcomes than chemotherapy among all patients who underwent randomization. These progression-free survival results from the CheckMate 8HW trial are consistent with previous data of first-line nivolumab plus ipilimumab from the nonrandomized CheckMate 142 study,[10] and they support the use of nivolumab plus ipilimumab in MSI-H or dMMR metastatic colorectal cancer.

The results of the KEYNOTE-177 trial showed that first-line pembrolizumab was associated with significantly better progression-free survival outcomes than chemotherapy in MSI-H or dMMR metastatic colorectal cancer.[5,17] Progression-free survival with pembrolizumab was 48% at 2 years, 42% at 3 years, and 34% at 5 years.[5,17,18] With respect to the subgroup analyses, progression-free survival outcomes were not better with pembrolizumab than with chemotherapy in patients with baseline *RAS* mutations (hazard ratio for disease progression or death, 1.19).[5] The effect of liver metastasis on the efficacy of pembrolizumab is unclear; in a cohort trial, significantly shorter progression-free survival was noted with PD-1 or PD-L1 inhibitor monotherapy regimens in patients with liver metastases than in patients with nonliver metastases (hazard ratio for disease progression or death, 2.60 [95% CI, 1.37 to 4.92];



The New England Journal of Medicine is produced by NEJM Group, a division of the Massachusetts Medical Society.
Downloaded from nejm.org at Shanghai Jiao Tong University on November 29, 2024. For personal use only.
No other uses without permission. Copyright © 2024 Massachusetts Medical Society. All rights reserved.

**Table 1. Patient Demographics and Disease Characteristics at Baseline.***

| Characteristic | Nivolumab plus Ipilimumab (N=202) | Chemotherapy (N=101) |
|---|---|---|
| Median age (range) — yr | 62 (21–86) | 65 (26–87) |
| Age distribution — no. (%) | | |
| <65 yr | 117 (58) | 46 (46) |
| ≥65 yr | 85 (42) | 55 (54) |
| Sex — no. (%) | | |
| Male | 95 (47) | 45 (45) |
| Female | 107 (53) | 56 (55) |
| Race — no. (%)† | | |
| White | 176 (87) | 85 (84) |
| Asian | 19 (9) | 13 (13) |
| Black | 2 (1) | 2 (2) |
| Other | 5 (2) | 1 (1) |
| Geographic region — no. (%) | | |
| United States, Canada, and Europe | 133 (66) | 71 (70) |
| Asia | 19 (9) | 11 (11) |
| All other regions | 50 (25) | 19 (19) |
| ECOG performance-status score — no. (%)‡ | | |
| 0 | 111 (55) | 52 (51) |
| 1 | 91 (45) | 49 (49) |
| Colorectal cancer stage at initial diagnosis — no. (%)§ | | |
| II | 43 (21) | 17 (17) |
| III | 73 (36) | 35 (35) |
| IV | 85 (42) | 49 (49) |
| Colorectal cancer stage at enrollment — no. (%)§ | | |
| IVA | 80 (40) | 43 (43) |
| IVB | 60 (30) | 27 (27) |
| IVC | 62 (31) | 31 (31) |
| Tumor location — no. (%) | | |
| Right | 138 (68) | 68 (67) |
| Left | 64 (32) | 33 (33) |
| Sites of metastases by blinded review — no. (%)¶ | | |
| Liver | 76 (38) | 42 (42) |
| Lung | 44 (22) | 25 (25) |
| Peritoneum | 84 (42) | 43 (43) |
| Centrally confirmed MSI-H or dMMR status — no. (%) | | |
| Yes | 171 (85) | 84 (83) |
| No | 31 (15) | 17 (17) |
| MSS and pMMR | 21 (10) | 12 (12) |
| MSS or pMMR‖ | 6 (3) | 0 |
| Other** | 4 (2) | 5 (5) |



The New England Journal of Medicine is produced by NEJM Group, a division of the Massachusetts Medical Society.
Downloaded from nejm.org at Shanghai Jiao Tong University on November 29, 2024. For personal use only.
No other uses without permission. Copyright © 2024 Massachusetts Medical Society. All rights reserved.

**Table 1. (Continued.)**

| Characteristic | Nivolumab plus Ipilimumab (N=202) | Chemotherapy (N=101) |
|---|---|---|
| PD-L1 expression — no. (%)†† | | |
| <1% | 145 (72) | 80 (79) |
| ≥1% | 43 (21) | 12 (12) |
| *BRAF*, *KRAS*, and *NRAS* mutation status — no. (%) | | |
| *BRAF*, *KRAS*, and *NRAS* all wild type | 47 (23) | 23 (23) |
| *BRAF* mutation | 52 (26) | 24 (24) |
| *KRAS* or *NRAS* mutation | 43 (21) | 21 (21) |
| *BRAF* and *KRAS* or *NRAS* mutation | 5 (2) | 2 (2) |
| Unknown | 55 (27) | 31 (31) |
| Clinical history of Lynch syndrome — no. (%)‡‡ | | |
| Yes | 22 (11) | 17 (17) |
| No | 135 (67) | 49 (49) |
| Unknown | 44 (22) | 30 (30) |
| Not reported | 1 (<1) | 5 (5) |
| Previous systemic therapies — no. (%) | | |
| Any previous systemic therapy | 67 (33) | 32 (32) |
| Previous systemic therapy type — no./total no. (%) | | |
| Neoadjuvant | 7/67 (10) | 5/32 (16) |
| Adjuvant | 60/67 (90) | 27/32 (84) |
| Metastatic§§ | 2/67 (3) | 2/32 (6) |

* Data are shown for the 303 patients with unresectable or metastatic colorectal cancer and microsatellite-instability–high (MSI-H) or mismatch-repair–deficient (dMMR) status according to local testing who underwent randomization and had not previously received systemic treatment for metastatic disease.
† Race was reported by the patients.
‡ Eastern Cooperative Oncology Group (ECOG) performance status is assessed on a scale of 0 to 5, with 0 indicating no performance restrictions and higher scores indicating greater disability.
§ Colorectal cancer stage was determined with the use of the American Joint Committee on Cancer TNM [extent of the tumor, spread to the lymph nodes, and metastasis] staging system. Colorectal cancer stage at initial diagnosis was not reported in one patient in the nivolumab-plus-ipilimumab group.
¶ Metastatic sites were not reported in three patients in the nivolumab-plus-ipilimumab group. Patients may have had more than one site of metastasis.
‖ Among the patients in the nivolumab-plus-ipilimumab group, four patients had mismatch-repair–proficient (pMMR) tumors and were not tested for microsatellite instability; two patients had microsatellite stable (MSS) tumors and could not be evaluated for mismatch-repair status.
** A total of nine patients could not be evaluated or were not tested for both microsatellite instability and mismatch-repair status (four patients in the nivolumab-plus-ipilimumab group and five patients in the chemotherapy group).
†† Tumor cell programmed death ligand 1 (PD-L1) expression was indeterminate, could not be evaluated, or was not available for 14 patients in the nivolumab-plus-ipilimumab group and 9 patients in the chemotherapy group.
‡‡ The Lynch syndrome is an inherited genetic disorder that confers a predisposition to certain cancers including colorectal cancer.
§§ Four patients who had received previous systemic therapy for metastatic disease were randomly assigned in error to the first-line treatment groups — two patients to the nivolumab-plus-ipilimumab group and two patients to the chemotherapy group.

P=0.003).[19] In contrast, nivolumab plus ipilimumab showed a consistent benefit across all subgroups, including in patients with baseline *RAS* or *BRAF* mutations and baseline liver, lung, or peritoneal metastases.

In the primary analysis population of the CheckMate 8HW trial, in which central confirmation of MSI-H or dMMR status was obtained, an early and sustained separation of the progression-free survival curves began at approximately



The New England Journal of Medicine is produced by NEJM Group, a division of the Massachusetts Medical Society.
Downloaded from nejm.org at Shanghai Jiao Tong University on November 29, 2024. For personal use only.
No other uses without permission. Copyright © 2024 Massachusetts Medical Society. All rights reserved.

3 months. In some clinical studies, an early progression-free survival detriment has been observed with immune checkpoint inhibitors as compared with conventional chemotherapy with or without targeted therapies.[20] In the KEYNOTE-177 trial, in which the primary population was determined by the results of local testing for MSI-H or dMMR status, an initial crossing of the progression-free survival curves for pembrolizumab as compared with chemotherapy was observed, resolving at approximately 6 months.[5] Progression-free survival curves for all patients who underwent randomization according to local testing in the CheckMate 8HW trial showed an initial crossing of curves, a finding reminiscent of the results of the KEYNOTE-177 trial, although the crossing of curves resolved earlier and with less detriment. These results highlight the effect of

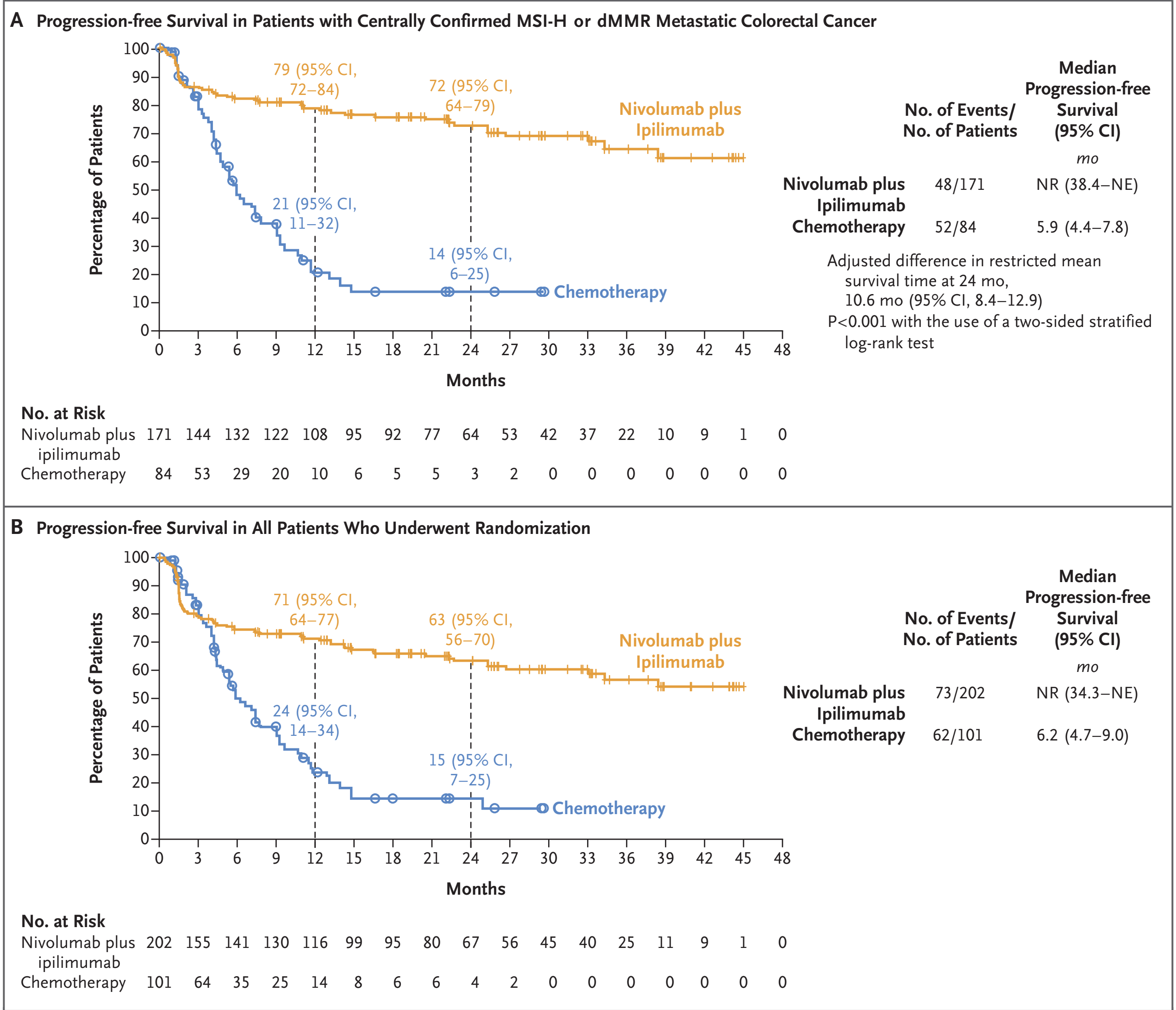


**Figure 2. Progression-free Survival by Blinded Review.**

Shown are Kaplan–Meier curves for progression-free survival with nivolumab plus ipilimumab as compared with chemotherapy, as assessed by blinded review in patients with microsatellite-instability–high (MSI-H) or mismatch-repair–deficient (dMMR) metastatic colorectal cancer and centrally confirmed MSI-H or dMMR status who had not previously received systemic treatment for metastatic disease (Panel A) and in the population of all the patients who underwent randomization (Panel B). The prespecified statistical criterion for superiority of nivolumab plus ipilimumab over chemotherapy at this interim analysis was 0.0209; the P value for the analysis met this criterion. Tick marks and circles indicate censored data. NE denotes could not be estimated, and NR not reached.



The New England Journal of Medicine is produced by NEJM Group, a division of the Massachusetts Medical Society.
Downloaded from nejm.org at Shanghai Jiao Tong University on November 29, 2024. For personal use only.
No other uses without permission. Copyright © 2024 Massachusetts Medical Society. All rights reserved.

**Table 2. Progression-free Survival by Blinded Review in Key Subgroups of Patients with Centrally Confirmed MSI-H or dMMR Metastatic Colorectal Cancer.**

| Subgroup | Disease Progression or Death | | 12-Mo Progression-free Survival (95% CI)* | |
|---|---|---|---|---|
| | Nivolumab plus Ipilimumab | Chemotherapy | Nivolumab plus Ipilimumab | Chemotherapy |
| | *no. of events/no. of patients* | | *percentage of patients* | |
| Overall | 48/171 | 52/84 | 79 (72 to 84) | 21 (11 to 32) |
| Age | | | | |
| <65 yr | 23/98 | 26/40 | 80 (70 to 86) | 24 (10 to 41) |
| ≥65 yr | 25/73 | 26/44 | 78 (66 to 86) | 17 (6 to 33) |
| Sex | | | | |
| Male | 20/79 | 25/38 | 79 (68 to 86) | 23 (9 to 41) |
| Female | 28/92 | 27/46 | 79 (68 to 86) | 19 (8 to 34) |
| Geographic region | | | | |
| United States, Canada, and Europe | 34/109 | 34/58 | 76 (67 to 83) | 26 (13 to 40) |
| Asia | 2/17 | 6/11 | 93 (61 to 99) | 0 |
| All other regions | 12/45 | 12/15 | 79 (64 to 89) | 10 (<1 to 34) |
| ECOG performance-status score | | | | |
| 0 | 24/97 | 27/45 | 79 (69 to 86) | 31 (16 to 47) |
| 1 | 24/74 | 25/39 | 78 (67 to 86) | 8 (1 to 22) |
| Tumor location | | | | |
| Right | 34/123 | 38/62 | 77 (68 to 84) | 19 (8 to 32) |
| Left | 14/48 | 14/22 | 83 (68 to 91) | 24 (8 to 45) |
| Liver metastases† | | | | |
| Yes | 12/55 | 23/32 | 85 (73 to 92) | 14 (4 to 31) |
| No | 36/114 | 29/52 | 76 (66 to 83) | 25 (12 to 41) |
| Lung metastases† | | | | |
| Yes | 19/37 | 14/16 | 56 (39 to 71) | 14 (2 to 37) |
| No | 29/132 | 38/68 | 85 (77 to 90) | 23 (12 to 36) |
| Peritoneal metastases† | | | | |
| Yes | 23/76 | 28/39 | 74 (62 to 83) | 13 (3 to 28) |
| No | 25/93 | 24/45 | 82 (73 to 89) | 28 (13 to 44) |
| PD-L1 expression | | | | |
| <1% | 34/122 | 42/69 | 79 (70 to 85) | 23 (12 to 36) |
| ≥1% | 10/43 | 9/12 | 83 (68 to 92) | 10 (<1 to 36) |
| *BRAF*, *KRAS*, and *NRAS* mutation status | | | | |
| *BRAF*, *KRAS*, and *NRAS* all wild type | 11/41 | 14/17 | 85 (70 to 93) | 0 |
| *BRAF* mutation | 16/50 | 11/22 | 73 (58 to 84) | 34 (12 to 59) |
| *KRAS* or *NRAS* mutation | 9/30 | 9/15 | 76 (56 to 88) | 29 (7 to 56) |
| Unknown | 10/46 | 16/28 | 84 (70 to 92) | 20 (5 to 41) |
| Lynch syndrome | | | | |
| Yes | 6/18 | 9/13 | 72 (46 to 87) | 23 (4 to 51) |
| No | 32/113 | 20/39 | 79 (70 to 85) | 25 (10 to 45) |
| Unknown | 10/39 | 20/27 | 82 (65 to 91) | 10 (2 to 27) |

* The percentages of patients with progression-free survival are Kaplan–Meier estimates. Percentages were not computed for subgroups with fewer than 10 patients per treatment group.

† Metastatic sites were determined by blinded review and were not reported in two patients with centrally confirmed MSI-H or dMMR status in the nivolumab-plus-ipilimumab group. Patients may have had more than one site of metastasis.



The New England Journal of Medicine is produced by NEJM Group, a division of the Massachusetts Medical Society.
Downloaded from nejm.org at Shanghai Jiao Tong University on November 29, 2024. For personal use only.
No other uses without permission. Copyright © 2024 Massachusetts Medical Society. All rights reserved.

**Table 3. Treatment-Related Adverse Events in All Treated Patients.***

| Event | Nivolumab plus Ipilimumab (N=200) | | Chemotherapy (N=88) | |
|---|---|---|---|---|
| | Any grade | Grade 3 or 4 | Any grade | Grade 3 or 4 |
| | *number of patients with event (percent)* | | | |
| Any treatment-related adverse event | 160 (80) | 46 (23) | 83 (94) | 42 (48) |
| Treatment-related serious adverse event | 38 (19) | 32 (16) | 17 (19) | 14 (16) |
| Treatment-related adverse event leading to discontinuation of any drug in the regimen | 33 (16) | 23 (12) | 28 (32) | 9 (10) |
| Treatment-related death† | 2 (1) | | 0 | |
| Treatment-related adverse event reported in ≥10% of patients in either group | | | | |
| Pruritus | 45 (22) | 0 | 4 (5) | 0 |
| Diarrhea | 42 (21) | 2 (1) | 45 (51) | 4 (5) |
| Hypothyroidism | 32 (16) | 2 (1) | 0 | 0 |
| Asthenia | 28 (14) | 2 (1) | 31 (35) | 5 (6) |
| Fatigue | 26 (13) | 1 (<1) | 12 (14) | 0 |
| Rash | 21 (10) | 2 (1) | 7 (8) | 1 (1) |
| Alanine aminotransferase increased | 20 (10) | 3 (2) | 3 (3) | 0 |
| Adrenal insufficiency | 20 (10) | 6 (3) | 0 | 0 |
| Nausea | 10 (5) | 0 | 41 (47) | 2 (2) |
| Decreased appetite | 10 (5) | 1 (<1) | 20 (23) | 1 (1) |
| Anemia | 5 (2) | 0 | 14 (16) | 3 (3) |
| Vomiting | 4 (2) | 0 | 18 (20) | 1 (1) |
| Neutropenia | 3 (2) | 0 | 19 (22) | 9 (10) |
| Alopecia | 3 (2) | 0 | 10 (11) | 0 |
| Stomatitis | 1 (<1) | 0 | 11 (12) | 0 |
| Neutrophil count decreased | 1 (<1) | 1 (<1) | 14 (16) | 6 (7) |
| Peripheral neuropathy | 0 | 0 | 12 (14) | 1 (1) |

* Data are shown for the patients who received at least one dose of the assigned treatment. All events between the first dose of treatment and 30 days after the last dose of treatment were reported. With respect to the patients who crossed over from the chemotherapy group to nivolumab plus ipilimumab (the crossover group), data collected on or after the date that the patient received the first dose of nivolumab plus ipilimumab were excluded, except for the number of deaths.

† Treatment-related adverse events leading to death were reported regardless of time frame. Treatment-related deaths in the group that received nivolumab plus ipilimumab were from myocarditis and pneumonitis (one patient each). In the chemotherapy group, one death (from acute myocarditis) occurred after the patient crossed over from chemotherapy to nivolumab plus ipilimumab and was not related to chemotherapy.

MSI-H or dMMR misdiagnoses and potentially improved efficacy with dual immunotherapy, given the resistance to immune checkpoint inhibitors observed in most cases of microsatellite-stable or mismatch repair–proficient metastatic colorectal cancer.[21] Among the 202 patients randomly assigned to the nivolumab-plus-ipilimumab group, 13% had microsatellite-stable or mismatch repair–proficient tumors according to central testing, a percentage that may have driven the initial detriment observed in the population of all patients who underwent randomization. This finding suggests that a confirmatory test that uses a validated immunohistochemical assay or



The New England Journal of Medicine is produced by NEJM Group, a division of the Massachusetts Medical Society.
Downloaded from nejm.org at Shanghai Jiao Tong University on November 29, 2024. For personal use only.
No other uses without permission. Copyright © 2024 Massachusetts Medical Society. All rights reserved.

polymerase-chain-reaction–based test may be beneficial in clinical practice to avoid misdiagnosis of MSI-H or dMMR status and to resolve the diagnosis when an initial test result is unclear.

In the CheckMate 142 trial, indirect comparisons suggested that the combination of nivolumab plus ipilimumab provided clinical benefit that was better than that with nivolumab monotherapy, with a favorable risk–benefit profile for patients with previously treated MSI-H or dMMR metastatic colorectal cancer.[11,22] The CheckMate 8HW trial is ongoing to assess the other primary end point of progression-free survival with nivolumab plus ipilimumab as compared with nivolumab among patients regardless of previous systemic treatment for metastatic disease. These data will allow for a direct assessment of the combination of nivolumab plus ipilimumab as compared with nivolumab monotherapy in a randomized trial and an understanding of the incremental benefit of dual immunotherapy in MSI-H or dMMR metastatic colorectal cancer.

The toxic effects of nivolumab plus ipilimumab were consistent with the known profiles of each individual component. The safety profile of nivolumab plus ipilimumab was different from that of chemotherapy, with fewer grade 3 or 4 treatment-related adverse events (23% vs. 48%). The median treatment duration and progression-free survival reported in the chemotherapy group in the current analyses were notably lower than those reported in the KEYNOTE-177 trial.[5] These differences in results can be attributed to differences in trial populations and increasing access to immunotherapies. Nevertheless, the median progression-free survival observed with chemotherapy in our trial was within the range reported in previous studies.[4,23,24]

It is important to note the limitations of this trial. First, the open-label trial design may have led to biases in the reporting of treatment assessments; however, this potential bias was mitigated by the use of blinded review for the primary end point. The concordance between the results from the blinded review and the investigator assessment of progression-free survival was high, which suggests that the open-label design did not influence efficacy assessments. Second, patient numbers in some of the prespecified subgroups were low, which limited interpretation of the data; of note, *RAS* mutation status was not available for 28% of the patients in this trial. Third, the underrepresentation of Black patients compromises the ability to extrapolate the results to that important subgroup. Fourth, the lower percentage of patients in the chemotherapy group than in the nivolumab-plus-ipilimumab group who completed questionnaires and the small size of the chemotherapy group should be considered when evaluating the health-related quality-of-life results.

In this trial, progression-free survival outcomes with nivolumab plus ipilimumab were superior to those with chemotherapy in the first-line treatment of MSI-H or dMMR metastatic colorectal cancer. Grade 3 and 4 treatment-related toxic effects were consistent with the established profiles of each individual drug, and no new safety concerns were identified.

Supported by Bristol Myers Squibb and Ono Pharmaceutical.

Disclosure forms provided by the authors are available with the full text of this article at NEJM.org.

A data sharing statement provided by the authors is available with the full text of this article at NEJM.org.

We thank the patients and their families for making this trial possible; the investigators, research staff, and the clinical trial team at Bristol Myers Squibb (Princeton, NJ) and Ono Pharmaceutical (Osaka, Japan) for CheckMate 8HW trial support; Janice Kaps-Trotter (Bristol Myers Squibb) for contributions as the global trial manager; Lixian Jin (Bristol Myers Squibb) for contributions as a clinical trial physician; Yingsi Yang (Bristol Myers Squibb) for contributions on statistical analyses; Carine Cabilla for clinical operations support; Vanessa King and David Yu for diagnostics support; Steven Blum (Bristol Myers Squibb) for contributions on data collection, planning, and statistical analysis of health-related quality-of-life end points; Agilent Technologies for collaborative development of the MMR IHC panel pharmDx (Dako Omnis) assay (Santa Clara, CA) and Biocartis NV for collaborative development of the Idylla MSI test (Mechelen, Belgium); and Dhivya Ramalingam of Parexel for medical writing assistance, funded by Bristol Myers Squibb.

## APPENDIX

The authors' full names and academic degrees are as follows: Thierry Andre, M.D., Elena Elez, M.D., Ph.D., Eric Van Cutsem, M.D., Ph.D., Lars Henrik Jensen, M.D., Ph.D., Jaafar Bennouna, M.D., Ph.D., Guillermo Mendez, M.D., Michael Schenker, M.D., Ph.D., Christelle de la Fouchardiere, M.D., Maria Luisa Limon, M.D., Takayuki Yoshino, M.D., Ph.D., Jin Li, M.D., Heinz-Josef Lenz, M.D., Jose Luis Manzano Mozo, M.D., Ph.D., Giampaolo Tortora, M.D., Ph.D., Rocio Garcia-Carbonero, M.D., Laetitia Dahan, M.D., Ph.D., Myriam Chalabi, M.D., Ph.D., Rohit Joshi, M.D., Eray Goekkurt, M.D., Maria Ignez Braghiroli, M.D., Timucin Cil, M.D., Elvis Cela, Ph.D., Tian Chen, Ph.D., Ming Lei, Ph.D., Matthew Dixon, Pharm.D., Ph.D., Sandzhar Abdullaev, M.D., Ph.D., and Sara Lonardi, M.D.

The authors' affiliations are as follows: Sorbonne Université, Hôpital Saint Antoine, Assistance Publique–Hôpitaux de Paris, Unité Mixte de Recherche Scientifique 938, and SIRIC CURAMUS, Paris (T.A.), Hopital Foch, Suresnes (J.B.), and Institut Paoli-Calmettes (C.F.),



The New England Journal of Medicine is produced by NEJM Group, a division of the Massachusetts Medical Society.
Downloaded from nejm.org at Shanghai Jiao Tong University on November 29, 2024. For personal use only.
No other uses without permission. Copyright © 2024 Massachusetts Medical Society. All rights reserved.

and La Timone, Aix Marseille Université (L.D.), Marseille — all in France; Vall d'Hebron University Hospital and Institute of Oncology (VHIO), Universitat Autònoma de Barcelona, Barcelona (E.E.), Hospital Universitario Virgen del Rocío, Seville (M.L.L.), Institut Català d'Oncologia, Hospital Universitario Germans Trias i Pujol, Badalona (J.L.M.M.), and Hospital Universitario 12 de Octubre, Imas12, Medicine Department–UCM, Madrid (R.G.-C.) — all in Spain; University Hospitals Gasthuisberg and University of Leuven (KU Leuven), Leuven, Belgium (E.V.C.); the University Hospital of Southern Denmark, Vejle Hospital, Vejle (L.H.J.); Hospital Universitario Fundacion Favaloro, Buenos Aires (G.M.); Centrul de Oncologie Sf Nectarie, Craiova, Romania (M.S.); the National Cancer Center Hospital East, Chiba, Japan (T.Y.); Shanghai East Hospital, Shanghai, China (J.L.); the University of Southern California Norris Comprehensive Cancer Center, Los Angeles (H.-J.L.); Fondazione Policlinico Universitario A. Gemelli IRCCS, Rome (G.T.), and Veneto Institute of Oncology IOV-IRCCS, Padua (S.L.) — both in Italy; the Netherlands Cancer Institute, Amsterdam (M.C.); Cancer Research SA, Adelaide, SA, Australia (R.J.); Hematology-Oncology Practice Eppendorf (HOPE) and University Cancer Center Hamburg (UCCH), Hamburg, Germany (E.G.); the Institute of Cancer of São Paulo, São Paulo (M.I.B.); Adana City Education and Research Hospital, Adana, Turkey (T. Cil); and Bristol Myers Squibb, Princeton, NJ (E.C., T. Chen, M.L., M.D., S.A.)

## REFERENCES


1. Bray F, Laversanne M, Sung H, et al. Global cancer statistics 2022: GLOBOCAN estimates of incidence and mortality worldwide for 36 cancers in 185 countries. CA Cancer J Clin 2024;74:229-63.
2. National Cancer Institute Surveillance, Epidemiology, and End Results Program. SEER stat database: incidence — SEER22 (https://seer.cancer.gov/statfacts/html/colorect.html).
3. Gutierrez C, Ogino S, Meyerhardt JA, Iorgulescu JB. The prevalence and prognosis of microsatellite instability-high/mismatch repair-deficient colorectal adenocarcinomas in the United States. JCO Precis Oncol 2023;7:e2200179.
4. Venderbosch S, Nagtegaal ID, Maughan TS, et al. Mismatch repair status and BRAF mutation status in metastatic colorectal cancer patients: a pooled analysis of the CAIRO, CAIRO2, COIN, and FOCUS studies. Clin Cancer Res 2014;20:5322-30.
5. André T, Shiu K-K, Kim TW, et al. Pembrolizumab in microsatellite-instability–high advanced colorectal cancer. N Engl J Med 2020;383:2207-18.
6. Das R, Verma R, Sznol M, et al. Combination therapy with anti-CTLA-4 and anti-PD-1 leads to distinct immunologic changes in vivo. J Immunol 2015;194:950-9.
7. Wei SC, Duffy CR, Allison JP. Fundamental mechanisms of immune checkpoint blockade therapy. Cancer Discov 2018;8:1069-86.
8. Hellmann MD, Paz-Ares L, Bernabe Caro R, et al. Nivolumab plus ipilimumab in advanced non–small-cell lung cancer. N Engl J Med 2019;381:2020-31.
9. Larkin J, Chiarion-Sileni V, Gonzalez R, et al. Five-year survival with combined nivolumab and ipilimumab in advanced melanoma. N Engl J Med 2019;381:1535-46.
10. Lenz H-J, Van Cutsem E, Luisa Limon M, et al. First-line nivolumab plus low-dose ipilimumab for microsatellite instability-high/mismatch repair-deficient metastatic colorectal cancer: the phase II CheckMate 142 study. J Clin Oncol 2022;40:161-70.
11. Overman MJ, Lonardi S, Wong KYM, et al. Durable clinical benefit with nivolumab plus ipilimumab in DNA mismatch repair-deficient/microsatellite instability-high metastatic colorectal cancer. J Clin Oncol 2018;36:773-9.
12. Overman MJ, McDermott R, Leach JL, et al. Nivolumab in patients with metastatic DNA mismatch repair-deficient or microsatellite instability-high colorectal cancer (CheckMate 142): an open-label, multicentre, phase 2 study. Lancet Oncol 2017;18:1182-91.
13. André T, Lonardi S, Wong KYM, et al. Nivolumab plus low-dose ipilimumab in previously treated patients with microsatellite instability-high/mismatch repair-deficient metastatic colorectal cancer: 4-year follow-up from CheckMate 142. Ann Oncol 2022;33:1052-60.
14. Opdivo (nivolumab). Summary of product characteristics. Dublin: Bristol Myers Squibb Pharma, September 2023 (https://www.ema.europa.eu/en/documents/product-information/opdivo-epar-product-information_en.pdf).
15. Opdivo (nivolumab). Highlights of prescribing information. Princeton, NJ: Bristol Myers Squibb, October 2023 (https://packageinserts.bms.com/pi/pi_opdivo.pdf).
16. Opdivo (nivolumab) prescribing information. Osaka, Japan: Ono Pharmaceutical, December 2023 (https://www.opdivo.jp/system/files/2024-07/OPD_PI.pdf). (In Japanese.)
17. Diaz LA Jr, Shiu K-K, Kim T-W, et al. Pembrolizumab versus chemotherapy for microsatellite instability-high or mismatch repair-deficient metastatic colorectal cancer (KEYNOTE-177): final analysis of a randomised, open-label, phase 3 study. Lancet Oncol 2022;23:659-70.
18. Shiu K-K, André T, Kim TW, et al. Pembrolizumab versus chemotherapy in microsatellite instability-high (MSI-H)/mismatch repair-deficient (dMMR) metastatic colorectal cancer (mCRC): 5-year follow-up of the randomized phase III KEYNOTE-177 study. Ann Oncol 2023;34:Suppl 2:S1271-S1272. abstract (https://www.annalsofoncology.org/article/S0923-7534(23)04168-6/fulltext).
19. Saberzadeh-Ardestani B, Jones JC, McWilliams RR, et al. Metastatic site and clinical outcome of patients with deficient mismatch repair metastatic colorectal cancer treated with an immune checkpoint inhibitor in the first-line setting. Eur J Cancer 2024;196:113433.
20. Borcoman E, Kanjanapan Y, Champiat S, et al. Novel patterns of response under immunotherapy. Ann Oncol 2019;30:385-96.
21. Cohen R, Hain E, Buhard O, et al. Association of primary resistance to immune checkpoint inhibitors in metastatic colorectal cancer with misdiagnosis of microsatellite instability or mismatch repair deficiency status. JAMA Oncol 2019;5:551-5.
22. Overman MJ, Lenz H-J, Andre T, et al. Nivolumab (NIVO) ± ipilimumab (IPI) in patients (pts) with microsatellite instability-high/mismatch repair-deficient (MSI-H/dMMR) metastatic colorectal cancer (mCRC): five-year follow-up from CheckMate 142. J Clin Oncol 2022;40:3510. abstract (https://ascopubs.org/doi/10.1200/JCO.2022.40.16_suppl.3510).
23. Cohen R, Buhard O, Cervera P, et al. Clinical and molecular characterisation of hereditary and sporadic metastatic colorectal cancers harbouring microsatellite instability/DNA mismatch repair deficiency. Eur J Cancer 2017;86:266-74.
24. Innocenti F, Ou F-S, Qu X, et al. Mutational analysis of patients with colorectal cancer in CALGB/SWOG 80405 identifies new roles of microsatellite instability and tumor mutational burden for patient outcome. J Clin Oncol 2019;37:1217-27.


*Copyright © 2024 Massachusetts Medical Society.*



The New England Journal of Medicine is produced by NEJM Group, a division of the Massachusetts Medical Society.
Downloaded from nejm.org at Shanghai Jiao Tong University on November 29, 2024. For personal use only.
No other uses without permission. Copyright © 2024 Massachusetts Medical Society. All rights reserved.
