# Supplementary material for: Dual-immunotherapy triumphs: redefining deficient mismatch repair or high microsatellite instability metastatic colorectal cancer first-line treatment
Source: Signal Transduct Target Ther. 2025 Jul 15;10:234. doi: 10.1038/s41392-025-02322-8 (PMC12264035; doi:10.1038/s41392-025-02322-8)
Supplement: Supplementary file 4 — REF4 [file 41392_2025_2322_MOESM4_ESM.pdf]

Genetics; Non-Financial Interests, Principal Investigator, TAS-120-202: Taiho; Non-Financial Interests, Principal Investigator, Krystal-10: Mirati; Non-Financial Interests, Principal Investigator, ADP-0033: Adaptimmune; Non-Financial Interests, Principal Investigator, ACT16902: Sanofi; Non-Financial Interests, Principal Investigator, C4201002 ; SGNB6A: Pfizer; Non-Financial Interests, Principal Investigator, RLY-4008: Relay Therapeutics; Non-Financial Interests, Principal Investigator, CC-90011: Celgene/BMS; Non-Financial Interests, Principal Investigator, Loxo-IDH ; Loxo-RAS: Loxo/Lilly; Non-Financial Interests, Principal Investigator: AstraZeneca; Non-Financial Interests, Principal Investigator, SN-201 study: Sotio; Non-Financial Interests, Principal Investigator, Tropics-03: Gilead; Non-Financial Interests, Principal Investigator, BI1403: Boehringer Ingelheim; Non-Financial Interests, Principal Investigator, CA120-1001: BMS. T. Mazard: Financial Interests, Personal, Advisory Board: Pierre Fabre, Servier; Financial Interests, Personal, Invited Speaker: Servier, Sanofi, Pierre Fabre; Financial Interests, Personal, Other, Development of clinical cases for regional meetings: MERCK SERONO; Financial Interests, Personal, Writing Engagement: Galapagos; Financial Interests, Institutional, Coordinating PI: Amgen; Non-Financial Interests, Advisory Role, development of guidelines about molecular testing in colorectal cancers: INCA; Other, travel grant: Pierre Fabre, Merck Serono, Sanofi, MSD. S. Pernot: Financial Interests, Personal, Invited Speaker: bayer, amgen, Pierre Fabre, AstraZeneca; Financial Interests, Personal, Advisory Board: MSD, BMS, Servier, Merck, Takeda. M.E. Elez Fernandez: Financial Interests, Personal, Advisory Board: Hoffman La - Roche, Servier, Amgen, Merck Serono, Sanofi, Bayer, Pierre Fabre, MSD, Takeda, Boehringer Ingelheim, Cure Teq AG, Repare Therapeutics Inc., RIN Institute Inc., Janssen; Financial Interests, Personal, Invited Speaker: Organon, Novartis, Pfizer, BMS, Lilly, Medscape; Financial Interests, Personal, Other, Educational training: Seagen International GmbH; Financial Interests, Institutional, Funding: Hoffmann-La Roche Ltd, Sanofi Aventis Recherche & Développement, Amgen Inc., Boehringer Ingelheim, Novartis Farmacéutica SA, Bristol-Myers Squibb International Corporation, BeiGene, HalioDX SAS, Janssen-Cilag SA, Merck Health KGAA, Merck Sharp & Dohme de España SA, PharmaMar SA, Servier, Taiho Pharma USA Inc, Hutchison MediPharma International, Menarini, Merus NV, Pfizer, Mirati, Array Biopharma Inc, AstraZeneca Pharmaceuticals LP, Celgene International SARL, Debiopharm International SA, Genentech Inc, MedImmune, Abbvie Deutschland Gmbh & Co KG, Bayer Pharma AG, Bioncotech Therapeutics, S.L., Biontech Rna Pharmacuticals GMBH, Biontech Small Molecules GMBH, Boehringer Ingelheim de España S.A., Daiichi Sankyo, Inc, Gercor, Hutchinson Medipharma Limited, Iovance Biotherapeutics, Inc., Janssen Research & Development, Menarini Ricerche SPA, Merck, Sharp & Dohme De España S.A., Nouscom SRL., Pledpharma AB, Redx Pharma PLC, Scandion Oncology, Seattle Genetics Inc., Sotio A.S., Wntresearch AB; Non-Financial Interests, Other, Coordinator of the SEOM +MIR Section of Residents and Young Assistants: Sociedad Española de Oncología Médica (SEOM); Non-Financial Interests, Other, Speaker of the ESMO Academy: European Society for Medical Oncology (ESMO); Non-Financial Interests, Other, Volunteer member of the ASCO Annual Meeting Scientific Program Committee: Developmental Therapeutics — Immunotherapy: American Society of Clinical Oncology (ASCO); Non-Financial Interests, Leadership Role, Member of the Scientific Program Committee and Developmental Therapeutics-Immunotherapy Track Leader, 2023-2024 term: American Society for Clinical Oncology (ASCO); Non-Financial Interests, Other, Member of the Scientific Committee 2024: European Society for Medical Oncology (ESMO); Other, Travel, Accommodations, Expenses: Roche, Merck Serono, Sanofi, Amgen, Array BioPharma, Servier, Bristol-Myers Squibb. M.J. Overman: Financial Interests, Personal, Advisory Board: Roche, BMS, Medimmune, Merck, Amgen, Takeda, Janssen, PFizer, Array, Gritstone, Nousome, Atreca, Bayer, Summit, Agenus, Regeneron, Astellas; Financial Interests, Institutional, Coordinating PI: roche, lilly, merck, bms, phanes, nouscom. L. Salvatore: Financial Interests, Personal, Advisory Board: Takeda, Pierre-Fabre, GSK, servier, Bayer, MSD, Amgen, Merck, Leopharma; Financial Interests, Personal, Invited Speaker: Takeda, Pierre Fabre, Servier, Bayer, MSD, Incyte, AstraZeneca, Amgen, Merck. R. Guimbaud: Financial Interests, Personal, Advisory Board: ADACAP, BMS; Financial Interests, Personal, Invited Speaker: MSD, AstraZeneca, Bayer, Esteve, Pierre Fabre, Merck. C. Cremolini: Financial Interests, Personal, Advisory Board: Roche, MSD, Amgen, Pierre Fabre, Nordic Pharma, Takeda; Financial Interests, Personal, Invited Speaker: Bayer, Servier, Merck Serono; Financial Interests, Institutional, Coordinating PI: Roche, Bayer, Servier, Merck; Financial Interests, Institutional, Local PI: seagen, Hutchinson. D. Tougeron: Financial Interests, Personal, Advisory Board: AstraZeneca, Sanofi, Amgen, MSD, Roche, Servier, Pierre Fabre, BMS, Bayer; Non-Financial Interests, Member of Board of Directors: Federation francophone de cancerologie digestive. F. Pietrantonio: Financial Interests, Personal, Advisory Board: Amgen, Merck-Serono, MSD, Bayer, Astellas, Takeda, Ipsen, GSK, Johnson&Johnson, Rottapharm; Financial Interests, Personal, Invited Speaker: Amgen, Merck-Serono, BMS, Lilly, Servier, Bayer, Pierre-Fabre, AstraZeneca, Astellas, Daiichi Sankyo, Takeda; Financial Interests, Institutional, Research Grant: BMS, AstraZeneca, Incyte, Agenus; Financial Interests, Institutional, Coordinating PI: Lilly, Amgen. All other authors have declared no conflicts of interest.

https://doi.org/10.1016/j.annonc.2024.08.609

541P Nivolumab (NIVO) plus ipilimumab (IPI) vs chemotherapy (chemo) as first-line (1L) treatment for microsatellite instability-high/mismatch repair-deficient (MSI-H/dMMR) metastatic colorectal cancer (mCRC): Subgroup efficacy and expanded safety analyses from CheckMate 8HW

T. André[1], S. Lonardi[2], H.J. Lenz[3], L.H. Jensen[4], E. Van Cutsem[5], Y. Touchefeu[6], R. Garcia-Carbonero[7], D. Tougeron[8], G.A. Mendez[9], M. Schenker[10], C. de la Fouchardiere[11], M.L. Limon[12], T. Yoshino[13], J. Li[14], J.L. Manzano Mozo[15], E. Cela[16], T. Chen[17], M. Lei[18], L. Jin[19], M.E. Elez Fernandez[20]

[1]Assistance Publique Hôpitaux de Paris, Sorbonne Université, and Hôpital Saint Antoine, Paris, France; [2]Medical Oncology, Veneto Institute of Oncology IOV-IRCCS, Padua, Italy; [3]Medical Oncology Department, University of Southern California - Norris Comprehensive Cancer Center, Los Angeles, CA, USA; [4]Department of Oncology, University Hospital of Southern Denmark, Vejle Hospital, Vejle, Denmark; [5]Digestive Oncology Department, University Hospitals Gasthuisberg and University of Leuven (KU Leuven), Leuven, Belgium; [6]Medical Oncology, Centre Hospitalier Universitaire de Nantes, Nantes, France; [7]Medical Oncology Department, Hospital Universitario 12 de Octubre Imas12, UCM, Madrid, Spain; [8]Medical Oncology, Centre Hospitalier Universitaire de Poitiers, Poitiers, France; [9]Oncology Department, Hospital Universitario Fundacion Favaloro, Buenos Aires, Buenos Aires, Argentina; [10]Medical Oncology Department, Centrul de Oncologie Sf Nectarie, Craiova, Romania; [11]Medical Oncology Department, Centre Léon Bérard, Lyon, France; [12]Oncology Dept., Hospital Universitario Virgen del Rocio, Seville, Spain; [13]Department of Gastroenterology and Gastrointestinal Oncology, National Cancer Center Hospital East, Chiba, Japan; [14]Medical Oncology, Shanghai East Hospital, Shanghai, China; [15]Medical Oncology, Institut Català d'Oncologia, Badalona, Spain; [16]Oncology Global Group Development, Bristol Myers Squibb, Princeton, NJ, USA; [17]Stats, Bristol Myers Squibb, Princeton, NJ, USA; [18]Translational Medicine, Bristol Myers Squibb, Princeton, USA; [19]Oncology Clinical Development, Bristol Myers Squibb, Princeton, NJ, USA; [20]Medical Oncology Dept., Vall d'Hebron University Hospital and Institute of Oncology (VHIO), Barcelona, Spain

Background: In the ongoing phase 3 CheckMate 8HW study, 1L NIVO + IPI demonstrated superior progression-free survival (PFS) vs chemo (HR 0.21; 97.91% CI 0.13—0.35; $P < 0.0001$) in patients (pts) with centrally confirmed MSI-H/dMMR mCRC. We report additional subgroup efficacy and expanded safety analyses from the prespecified interim analysis of 1L NIVO + IPI vs chemo.

Methods: The study design has been described previously. Pts were enrolled based on MSI-H/ dMMR per local testing. Central immunohistochemistry and polymerase chain reaction—based tests were used to confirm MSI-H/dMMR.

Results: In total, 202 pts were randomized to NIVO + IPI and 101 to chemo. MSI-H/dMMR was confirmed by either central test in 171 and 84 pts in the NIVO + IPI and chemo arms, respectively; 27 and 12 pts were identified as microsatellite stable/mismatch repair-proficient (MSS/pMMR) by central test. Median follow-up was 31.5 mo (range 6.1—48.4). Median duration of treatment was 13.5 mo with NIVO + IPI (80% of pts received all 4 doses of IPI) and 4.0 mo with chemo. Improved PFS with NIVO + IPI vs chemo was observed across key subgroups in pts with centrally confirmed MSI-H/dMMR (**Table**). Among all treated pts, grade 3/4 treatment-related adverse events (TRAEs) with NIVO + IPI occurred in 32/140 (23%) and 14/60 (23%) pts aged < 70 and ≥ 70 yr, respectively; 27/58 (47%) and 15/30 (50%) with chemo. Median time to onset of non-endocrine immune-mediated AEs (IMAEs) ranged from 4.9 wk for rash to 13.3 wk for diarrhea; endocrine IMAEs ranged from 6.1 wk for hyperthyroidism to 15.6 wk for diabetes.

Table: 541P

	NIVO + IPI	Chemo	HR (95% CI)
	Median PFS (95% CI), mo		
MSI-H/dMMR[a] (n = 255)	NR (38.4—NE)	5.8 (4.4—7.8)	0.21 (0.13—0.35)
Age, yr			
< 50 (n = 62)	NR (33.1—NE)	5.6 (3.4—10.9)	0.21 (0.10—0.48)
≥ 70 (n = 82)	NR (20.5—NE)	5.9 (3.7—7.8)	0.27 (0.14—0.54)
Metastasis			
Liver			
Y (n = 87)	NR (38.4—NE)	5.9 (4.3—9.2)	0.11 (0.05—0.25)
N (n = 166)	NR (34.3—NE)	5.4 (4.2—9.6)	0.28 (0.17—0.46)
Peritoneal			
Y (n = 115)	NR (34.3—NE)	4.4 (3.2—7.1)	0.19 (0.10—0.34)
N (n = 138)	NR (38.4—NE)	7.4 (5.4—11.6)	0.23 (0.13—0.42)
KRAS/NRAS mutant (n = 45)	NR (33.1—NE)	5.7 (1.4—14.8)	0.24 (0.09—0.63)
**MSS/pMMR**[a] (n = 39)	1.9 (1.5—5.8)	11.5 (2.0—NE)	1.56 (0.66—3.70)

[a]By central test; both central tests not available, n = 9. NE, not estimable; NR, not reached.

Conclusions: PFS consistently favored 1L NIVO + IPI over chemo in all subgroups of pts with MSI-H/dMMR mCRC. The safety profile of NIVO + IPI was consistent in pts aged < 70 and ≥ 70 yr. These results further substantiate NIVO + IPI as a 1L treatment option for pts with MSI-H/dMMR mCRC.

Clinical trial identification: NCT04008030.

Editorial acknowledgement: Writing and editorial assistance were provided by Dominic Singson, MD, of Parexel, funded by Bristol Myers Squibb

Legal entity responsible for the study: Bristol Myers Squibb.

Funding: Bristol Myers Squibb

Disclosure: T. André: Financial Interests, Personal, Advisory Board, Advosiry Board on February 12, 2021: Astellas pharma; Financial Interests, Personal, Advisory Board, Advisory Board on February 2021: Kaleido Biosciences; Financial Interests, Personal, Invited Speaker, and advisory board 2021: Amgen; Financial Interests, Personal, Invited Speaker, Ivited speaker in a symposuim december 2020: AstraZeneca; Financial Interests, Personal, Advisory Board, and consultant fees and consultant contract 2021 and 2022: Bristol-Myers Squibb; Financial Interests, Personal, Advisory Board, Advisory board in Janaury 2020: Clovis; Financial Interests, Personal, Advisory Board, Advisory board in january 2020: Gritstone Oncology; Financial Interests, Personal, Advisory Board, Advisory board 2020: Haliodx; Financial Interests, Personal, Advisory Board, and consultant fees/consultant contract and invited speaker: MSD Oncology; Financial Interests, Personal, Invited Speaker, and other: Pierre Fabre; Financial Interests, Personal, Invited Speaker, in an symposuim in 2020: Roche; Financial Interests, Personal, Invited Speaker, in a meeting in 2019: Ventana; Financial Interests, Personal, Invited Speaker, In a educational meeting in 2019: Sanofi; Financial Interests, Personal, Advisory Board, in a symposuim in 2020: Servier; Financial Interests, Personal, Advisory Board, Consultant with personnal fees and invited speaker: Servier; Financial Interests, Personal, Advisory Board, in 2019: GSK; Financial Interests, Personal, Invited Speaker, in 2020 and 2021: GSK; Financial Interests, Personal, Invited Speaker, Virtual symposuimLecture: 1 MSI-H CRC: Implementation of Immunotherapy in clinical practice (30 minutes) — (this will be pre-recorded)Q&A — Live Q&A — (10 minutes) (on July 2, 2021): MSD Oncology; Financial Interests, Personal, Invited Speaker, June 2022: Sanofi; Financial Interests, Personal, Advisory Board, Contract 2021, 2022, 2023: Merck & Co., Inc; Financial Interests, Personal, Advisory Board, Contrat 2019, 2020, 2021, 2022: BMS; Financial Interests, Personal, Advisory Board, Contract 2021: Gritstone Oncologie; Financial Interests, Personal, Invited Speaker, June 2022 and June 2023 during ESMO GI meeting: Seagen; Financial Interests, Personal, Writing Engagement, Contract of consulting 2021 and 2022 and 2023: MSD Oncology; Financial Interests, Personal, Advisory Board, September 2022: GSK, Seagen; Financial Interests, Personal, Writing Engagement, Contract of consulting 2022 and 2023: GSK; Financial Interests, Personal, Writing Engagement, Contract of consulting 2022: Nordic Pharma; Financial Interests, Personal, Invited Speaker, October 2022: Merck Serono; Financial Interests, Personal, Other, Educationnal in 2022: Roche; Financial Interests, Personal, Other, Contract of consulting 2020, 2021, 2022,2023: Servier; Financial Interests, Personal, Advisory Board, Gilead Sciences Global GI Advisory Board on January 18, 2023: Aptitude Health, Glilead; Financial Interests, Personal, Other, Consultant like member of the SGNTUC-029 Steering Committee (the "Steering Committee") Services related to the Company's Study titled "An Open-label Randomized Phase 3 Study of Tucatinib in Combination with Trastuzumab and mFOLFOX6 versus mFOLFOX6 given with or without either Cetuximab or Bevacizumab as First-line Treatment for Subjects with HER2+ Metastatic Colorectal Cancer" (collectively, the "Services"). Including teleconferences and board preparation befor the board (6h) Participation of the Board on September 2022 in Paris, September 10, 2022 MOUNTAINEER-03 Steering Committee Meeting during ESMO: Seagen; Financial Interests, Personal, Other, Consultant like member of the to be a member of the Colorectal Program Scientific Advisory Committee (SAC). In charges for reviewing V940 Pembro on Engagezone (as per your contract in ref).and also an inrtview about Pembrolizumab for MSI/dMMR Metastaic colo-rectal 2023: MSD Oncology; Financial Interests, Personal, Advisory Board, but also formation for the takeda team, and consultant for TakedaContract beetween 1 sept 2023 and 15 october 2024: Takeda; Financial Interests, Personal, Advisory Board, Board Meeting (October 19, 2023): Abbvie; Financial Interests, Personal, Invited Speaker, Emerging Role of Biomarkers in Gastrointestinal Cancers Webcast Engagement reference : Wenibar: MSD; Financial Interests, Personal, Invited Speaker, Preparation and resentation at the Seagen ESMO GI 2023 Scientific Exchange HER2 as an emerging target for the treatment of mCRC in the context MOUNTAINEER03: Seagen; Financial Interests, Personal, Advisory Board, Fruquitinib: Takeda; Financial Interests, Personal, Invited Speaker, Oral speaker in a Symposuim 2 and 3 February 2024, GI ancer MSI: MSD; Financial Interests, Personal, Invited Speaker, Management of CRC: Aptitude Health; Financial Interests, Personal, Invited Speaker, symposuim JFHOD GI CancerPARIS15 03-2024: Servier; Financial Interests, Institutional, Coordinating PI, PI Garnet study: GSK; Financial Interests, Institutional, Coordinating PI, Keynote 164 and 171 and 811 and C08: MSD; Financial Interests, Institutional, Coordinating PI, BMS CA209-8HW, BMS CA209-142, BMS CA209-577: BMS; Financial Interests, Institutional, Coordinating PI, SPOTLIGHT study: Astellas; Financial Interests, Personal, Steering Committee Member, and internationnal PI (trial chair Solstice study): Servier; Financial Interests, Personal, Steering Committee Member, MOUNTAINEER and MOUNTAINEER-03 study (PI for France) 2022, 2023: Seagen; Financial Interests, Institutional, Local PI, A RANDOMIZED, OPEN-LABEL, PHASE 2 STUDY OF BOTENSILIMAB (AGEN1181) AS MONOTHERAPY AND IN COMBINATION WITH BALSTILIMAB (AGEN2034) OR INVESTIGATOR'S CHOICE STANDARD OF CARE (REGORAFENIB OR TRIFLURIDINE AND TIPIRACIL) FOR THE TREATMENT OF REFRACTORY METASTATIC COLORECTAL CANCER: Agenus; Financial Interests, Institutional, Local PI, Study wuith traztuzumab derxtecan in mCRC C study: Daichi; Non-Financial Interests, Member of Board of Directors, Investigator: Gercor group; Non-Financial Interests, Member of Board of Directors, President since october 2022: ARCAD Foundation; Other, DMC Meeting by Teleconference on November 7, 2023 5member of steering comittee of A randomized Phase 2 Study of ompenaclid versus placebo in combination with FOLFIRI plus bevacizumab in patients withpreviously treated RAS mutant advanced or metastatic colorectal cancer (RGX-202-002): Inspira. S. Lonardi: Financial Interests, Personal, Advisory Board: Amgen, merck serono, lilly, Servier, AstraZeneca, MSD, Incyte, Daiichi-Sankyo, Bristol-Myers Squibb, Astellas, GSK, Takeda, Bayer, Rottapharm; Financial Interests, Personal, Invited Speaker: Pierre-Fabre, GSK, Roche, Servier, Amgen, Bristol-Myers Squibb, Incyte, Lilly, Merck Serono, MSD, AstraZeneca; Financial Interests, Institutional, Coordinating PI: Amgen, Merck Serono, Bayer, Roche, Lilly, AstraZeneca, Bristol -Myers Squibb; Non-Financial Interests, Member of Board of Directors, Italian No-Profit Oncology Research Foundation supporting academic Clinical trials: GONO Foundation. H.J. Lenz: Financial Interests, Personal, Advisory Board, Advisory Role and Lectures: Bayer; Financial Interests, Personal, Advisory Board, Advisory ROle and Lectures: Merck; Financial Interests, Personal, Advisory Board, Advisory Role and lecture: ROche; Financial Interests, Personal, Advisory Board: Jazz Pharmaceuticals, oncocyte, Orion, Astellas, BMS; Financial Interests, Personal, Advisory Board, Scientific Advisory Board: 3T Biosscience, Adagene; Financial Interests, Personal, Advisory Board, Advisory Board: Fulgent, G1 Therapeutics, BioNtech, Merck KG; Financial Interests, Personal, Advisory Board, advisory board: Cardiff; Financial Interests, Personal, Stocks/Shares, Advisory Board: Fulgent; Financial Interests, Personal, Ownership Interest, stockoptions: Breakbio. L.H. Jensen: Financial Interests, Institutional, Research Funding: MDX, 2cureX, Incyte, Bristol Myers Squibb, Roche, Pfizer. E. Van Cutsem: Financial Interests, Personal, Advisory Board: Abbvie, Agenus, ALX, Arcus Biosciences, Astellas, AstraZeneca, Bayer, BeiGene, Biontech, Boehringer Ingelheim, Bristol-Myers Squibb, Daiichi, Debiopharma, Elmedix, Eisai, GSK, Hoopika Biotech, Incyte, Ipsen, Lilly, Merck Sharp & Dohme, Merck KGaA, MIrati, Novartis, Nordic, Pierre Fabre, Seattle Genetics, Servier, Simcere, Takeda, Taiho, Terumo; Financial Interests, Personal, Invited Speaker: Amgen, Pfizer. R. Garcia-Carbonero: Financial Interests, Personal, Advisory Board: AAA, Advanz Pharma, Amgen, Astellas, Bayer, BMS, Boerhringer, Esteve, Hutchmed, Ipsen, Midatech Pharma, MSD, Novartis, PharmaMar, Servier, Takeda; Financial Interests, Institutional, Research Grant: BMS, MSD, Pfizer; Non-Financial Interests, Leadership Role, Global PI of investigator-initiated clinical trials (AXINET, NICENEC, PEMBROLA): BMS, MSD, Pfizer; Non-Financial Interests, Leadership Role, Chair elect: European Neuroendocrine Tumor Society (ENETS); Non-Financial Interests, Leadership Role, Past presidente, Member of the Executive Committee: Grupo Español de Tumores Neuroendocrinos (GETNE); Other, Honoraria received by spouse for advisory board or invited speaker roles: AbbVie, AstraZeneca, BAYER, BOEHRINGER, BMS, GENOMICA, LILLY, MSD, MERCK, NOVARTIS, PFIZER, PHARMA MAR, ROCHE, SANOFI, SERVIER, TAKEDA. D. Tougeron: Financial Interests, Personal, Advisory Board: AstraZeneca, Sanofi, AMGEN, MSD, Roche, Servier, Pierre Fabre, BMS, Bayer; Non-Financial Interests, Member of Board of Directors: Federation francophone de cancerologie digestive. G.A. Mendez: Financial Interests, Institutional, Advisory Board: Roche, MSD, Bristol Myers Squibb, Amgen, Merck, Pfizer, Bayer, Roche, Pfizer; Financial Interests, Institutional, Speaker's Bureau: Amgen, Merck, MSD, Bristol Myers Squibb, SERVIER, Group Biotoscana; Financial Interests, Institutional, Expert Testimony: MSD, Bristol Myers Squibb; Financial Interests, Institutional, Other, Travel, Accommodations, Expenses: Merck, Grupo Biotoscana, Amgen, SERVIER, Pfizer. M. Schenker: Financial Interests, Institutional, Research Funding: Bristol Myers Squibb, Roche, Amgen, MSD, Pfizer/EMD Serono, Lilly, Astellas Pharma, AstraZeneca, GSK, Regeneron, Novartis, Abbvie, Gilead Sciences, Sanofi/Regeneron, Mylan, BIOVEN, Clovis Oncology, Tesaro, BeiGene, Five Prime Therapeutics. C. de la Fouchardiere: Financial Interests, Personal, Advisory Board: Bristol Myers Squibb, Amgen, SERVIER, Pierre Fabre, Eisai, MSD Oncology, Ipsen, Roche/Genentech, Lilly, Daiichi Sankyo, Astellas Pharma, Takeda; Financial Interests, Personal, Other, Travel, Accommodations, Expenses: Roche, Pierre Fabre, SERVIER, MSD Oncology, Amgen; Financial Interests, Institutional, Research Funding: Pierre Fabr, SERVIER, MSD. T. Yoshino: Financial Interests, Personal, Invited Speaker: Chugai Pharmaceutical Co., Ltd., Merck Biopharma Co., Ltd., Bayer Yakuhin, Ltd., Ono Pharmaceutical Co., Ltd., MSD K.K., Takeda Pharmaceutical Co., Ltd.; Financial Interests, Personal, Other, Consultancy: Sumitomo Corp.; Financial Interests, Institutional, Research Grant: Ono Pharmaceutical Co., Ltd, Sanofi K.K., MSD K.K., Taiho Pharmaceutical Co., Ltd., Molecular Health GmbH, Amgen K.K., Pfizer Japan Inc., Genomedia Inc., Sysmex Corp., Daiichi Sankyo Co., Ltd., Chugai Pharmaceutical Co., Ltd., Nippon Boehringer Ingelheim Co., Ltd., Eisai Co., Ltd., Roche Diagnostics K.K., FALCO Biosystems Ltd., Merus N.V., Bristol-Myers Squibb K.K., Medical & Biological Laboratories Co., LTD., Takeda Pharmaceutical Co., Ltd.. E. Cela: Financial Interests, Personal, Stocks or ownership: Bristol Myers Squibb; Financial Interests, Personal, Full or part-time Employment: Bristol Myers Squibb; Financial Interests, Personal, Other, Travel, Accommodations, Expenses: Bristol Myers Squibb. T. Chen: Financial Interests, Personal, Full or part-time Employment: Bristol-Myers Squib; Financial Interests, Institutional, Stocks or ownership: Bristol-Myers Squib; Financial Interests, Institutional, Royalties: Bristol-Myers Squib. M. Lei: Financial Interests, Personal, Full or part-time Employment: Bristol Myers Squibb; Financial Interests, Personal, Stocks or ownership: Bristol Myers Squibb; Financial Interests, Personal, Other, Inventor of pending BMS patents: Bristol Myers Squibb. L. Jin: Financial Interests, Personal, Full or part-time Employment: Bristol Myers Squibb; Financial Interests, Personal, Stocks or ownership: Bristol Myers Squibb; Financial Interests, Personal, Other, Travel, Accommodations, Expenses: Bristol Myers Squibb. M.E. Elez Fernandez: Financial Interests, Personal, Advisory Board: Hoffman La - Roche, Servier, Amgen, Merck Serono, Sanofi, Bayer, Pierre Fabre, MSD, Takeda, Boehringer Ingelheim, Cure Teq AG, Repare Therapeutics Inc., RIN Institute Inc., Janssen; Financial Interests, Personal, Invited Speaker: Organon, Novartis, Pfizer, BMS, Lilly, Medscape; Financial Interests, Personal, Other, Educational training: Seagen International GmbH; Financial Interests, Institutional, Funding: Hoffmann-La Roche Ltd, Sanofi Aventis Recherche & Développement, Amgen Inc., Boehringer Ingelheim, Novartis Farmacéutica SA, Bristol-Myers Squibb International Corporation, BeiGene, HalioDX SAS, Janssen-Cilag SA, Merck Health KGAA, Merck Sharp & Dohme de España SA, PharmaMar SA, Servier, Taiho Pharma USA Inc, Hutchison MediPharma International, Menarini, Merus NV, Pfizer, Mirati, Array Biopharma Inc, AstraZeneca Pharmaceuticals LP, Celgene International SARL, Debiopharm International SA, Genentech Inc, MedImmune, Abbvie Deutschland Gmbh & Co KG, Bayer Pharma AG, Bioncotech Therapeutics, S.L., Biontech Rna Pharmacuticals GMBH, Biontech Small Molecules GMBH, Boehringer Ingelheim de España S.A., Daiichi Sankyo, Inc, Gercor, Hutchinson Medipharma Limited, Iovance Biotherapeutics, Inc., Janssen Research & Development, Menarini Ricerche SPA, Merck, Sharp & Dohme De España S.A., Nouscom SRL., Pledpharma AB, Redx Pharma PLC, Scandion Oncology, Seattle Genetics Inc., Sotio A.S., Wntresearch AB; Non-Financial Interests, Other, Coordinator of the SEOM +MIR Section of Residents and Young Assistants: Sociedad Española de Oncología Médica (SEOM); Non-Financial Interests, Other, Speaker of the ESMO Academy: European Society for Medical Oncology (ESMO); Non-Financial Interests, Other, Volunteer member of the ASCO Annual Meeting Scientific Program Committee: Developmental Therapeutics — Immunotherapy: American Society of Clinical Oncology (ASCO); Non-Financial Interests, Leadership Role, Member of the Scientific Program Committee and Developmental Therapeutics-Immunotherapy Track Leader, 2023-2024 term: American Society for Clinical Oncology (ASCO); Non-Financial Interests, Other, Member of the Scientific Committee 2024: European Society for Medical Oncology (ESMO); Other, Travel, Accommodations, Expenses: Roche, Merck Serono, Sanofi, Amgen, Array BioPharma, Servier, Bristol-Myers Squibb. All other authors have declared no conflicts of interest.

https://doi.org/10.1016/j.annonc.2024.08.610

542P Long-term outcomes of neoadjuvant toripalimab with or without celecoxib in patients with dMMR/MSI-H locally advanced colorectal cancer: 3-month treatment cohort of the randomized phase II PICC trial

H. Hu[1], J. Zhang[2], X. Xie[2], L. Shi[2], Y. Cai[2], W. Li[2], Y. Xie[2], Z. Wu[2], G. Qin[2], J. Li[1], Y. Deng[3]

[1]Department of Oncology, The Sixth Affiliated Hospital, Sun Yat-sen University, Guangzhou, China; [2]Department of Medical Oncology, The Sixth Affiliated Hospital, Sun Yat-sen University, Guangzhou, China; [3]Department of Medical Oncology, The Sixth Affiliated Hospital, Sun Yat-sen University, Guangzhou, Guangdong, China

Background: Neoadjuvant PD-1 blockade has shown a high pathologic complete response (pCR) rate in patients with dMMR/MSI-H, locally advanced colorectal cancer (CRC). However, there is a lack of long-term survival data from prospective studies. Here, we report the 3-year oncologic outcomes from the 3-month treatment cohort of the PICC trial (NCT03926338).

Methods: The PICC study was a multi-cohort, randomized, phase 2 trial. Patients with clinical T3-4 and/or N+, and dMMR/MSI-H CRC were enrolled in the 3-month cohort and randomly assigned (1:1) to receive toripalimab 3 mg/kg, with or without celecoxib 200 mg twice daily from day 1 to 14 of each 14-day cycle, for 6 cycles before surgery. The primary endpoint of the pCR rate was met, showing significant improvement with neoadjuvant toripalimab with or without celecoxib compared to
