# Supplementary material for: Dual-immunotherapy triumphs: redefining deficient mismatch repair or high microsatellite instability metastatic colorectal cancer first-line treatment
Source: Signal Transduct Target Ther. 2025 Jul 15;10:234. doi: 10.1038/s41392-025-02322-8 (PMC12264035; doi:10.1038/s41392-025-02322-8)
Supplement: Supplementary file 5 — REF5 [file 41392_2025_2322_MOESM5_ESM.pdf]

JSMO 2025 KOBE

Nivolumab plus ipilimumab versus chemotherapy in MSI-H/dMMR metastatic colorectal cancer: CheckMate 8HW Asian subgroup analysis

Takayuki Yoshino,[1] Takeshi Kato,[2] Ying Yuan,[3] Taroh Satoh,[4] Jingdong Zhang,[5] Daisuke Kotani,[1] Kensei Yamaguchi,[6] Atsuo Takashima,[7] Hiroya Taniguchi,[8] Kentaro Yamazaki,[9] Hiroki Hara,[10] Koichi Suyama,[11] Yuji Miyamoto,[12] Yasuhiro Sakamoto,[13] Elvis Cela,[14] Tian Chen,[14] Ming Lei,[14] Lixian Jin,[14] Jin Li[15]

[1]National Cancer Center Hospital East, Chiba, Japan; [2]NHO Osaka National Hospital, Osaka, Japan; [3]The Second Affiliated Hospital of Zhejiang University School of Medicine, Hangzhao, China; [4]Osaka University Hospital, Osaka, Japan; [5]Liaoning Cancer Hospital and Institute, Shenyang, China; [6]The Cancer Institute Hospital of JFCR, Tokyo, Japan; [7]National Cancer Center Hospital, Tokyo, Japan; [8]Aichi Cancer Center Hospital, Nagoya, Japan; [9]Shizuoka Cancer Center, Shizuoka, Japan; [10]Saitama Cancer Center, Saitama, Japan; [11]Toranomon Hospital, Tokyo, Japan; [12]Kumamoto University Hospital, Kumamoto, Japan; [13]Osaki Citizen Hospital, Osaki, Japan; [14]Bristol Myers Squibb, Princeton, NJ, USA; [15]Shanghai East Hospital, Shanghai, China

Abstract number O2-1

Conflict of interest disclosures

- This study was supported by Bristol Myers Squibb and Ono Pharmaceutical Co., Ltd.
- Dr Takayuki Yoshino (of National Cancer Center Hospital East, Chiba, Japan) has the following disclosures:
  - Employee or adviser of company and/or profit-making organization: Sumitomo Corporation
  - Profit of stock: None
  - Patent fee: None
  - Lecturer fee: Chugai Pharma, Merck KGaA, Merck, Sharp & Dohme, Takeda
  - Manuscript fee: None
  - Research expenses from company: Amgen, Bristol Myers Squibb Japan, Chugai Pharma, Daiichi Sankyo, Eisai, FALCO Biosystems, Medical & Biological Laboratories Co. Ltd., Merck, Sharp & Dohme, Merus, Molecular Health, Ono Pharmaceutical, Pfizer, Roche, Sanofi, Sysmex, Taiho Pharmaceutical, Takeda
  - Contributions or endowed chair: None
  - Fees of testimony, judgment, comment, etc.: None
  - Presents or other payment: None
  - Representative of organization for clinical study receiving research expenses from company: None

Introduction

- The 2023 Pan-Asian ESMO guidelines for mCRC recommended pembrolizumab monotherapy for patients with MSI-H/dMMR mCRC in the 1L treatment setting[1]
- Despite improved PFS with 1L pembrolizumab vs chemo in MSI-H/dMMR mCRC, only 48% of patients were progression-free and alive at 2 years of follow-up in the KEYNOTE-177 study[2]
- Favorable efficacy outcomes have been observed with NIVO + IPI relative to single-agent immunotherapy in MSI-H/dMMR mCRC[3-5]
- In the phase 3 CheckMate 8HW study, NIVO + IPI demonstrated superior PFS vs chemo with no new safety signals as 1L treatment for MSI-H/dMMR mCRC[6]
- In this analysis, we present Asian subpopulation results of NIVO + IPI vs chemo in the 1L setting

1. Yoshino T, et al. *ESMO Open* 2023;8:101558. 2. Andre T, et al. *N Engl J Med* 2020;383:2207-2218. 3. Overman M, et al. *Lancet Oncol* 2017;18:1182-1191. 4. Overman M, et al. *J Clin Oncol* 2018;36:773-779. 5. Andre T, et al. *Ann Oncol* 2022;33:1052-1060. 6. Andre T, et al. *N Engl J Med* 2024;391:2014-2026.

CheckMate 8HW study design

- CheckMate 8HW is a randomized, multicenter, open-label phase 3 study[a]

Key eligibility criteria:
- Histologically confirmed unresectable or metastatic CRC
- MSI-H/dMMR status by local testing
- ECOG PS 0 or 1

Stratification factors:
- Prior lines of treatment (0 vs 1 vs ≥ 2)
- Primary tumor location (right vs left)

R 2:2:1

- NIVO 240 mg Q2W for 6 doses, followed by NIVO 480 mg Q4W[b]
- NIVO 240 mg + IPI 1 mg/kg Q3W for 4 doses, followed by NIVO 480 mg Q4W[b] — 1L setting: n = 202
- Investigator's choice chemo[c] (mFOLFOX6 or FOLFIRI ± bevacizumab or cetuximab) — 1L setting: n = 101

Treatment until disease progression, unacceptable toxicity, withdrawal of consent (all arms), or a maximum treatment duration of 2 years (NIVO and NIVO + IPI arms only)

Dual primary endpoints in patients with centrally confirmed MSI-H/dMMR status[d]:
- PFS by BICR[e] (NIVO + IPI vs chemo in the 1L setting)
- PFS by BICR[e] (NIVO + IPI vs NIVO across all lines)

Other select endpoints:
- Safety
- OS; PFS2 by investigator[e]; ORR by BICR[e]; PROs

- At data cutoff (October 12, 2023), the median follow-up[f] was 31.5 months (range, 6.1-48.4) in the global population and 20.2 months (range, 9.1-43.1) in the Asian subpopulation

[a]ClinicalTrials.gov. NCT04008030. [b]Patients with ≥ 2 prior lines are randomized only to the NIVO or NIVO + IPI arms. [c]Patients receiving investigator's choice of chemo are eligible to receive NIVO + IPI upon progression (crossover treatment). [d]Confirmed using either IHC and/or polymerase chain reaction-based tests. [e]Evaluated using RECIST v1.1. [f]Time between randomization and data cutoff.

Baseline characteristics

Characteristic (1L all randomized patients)	Category	Global population		Asian subpopulation	
		NIVO + IPI (n = 202)	Chemo (n = 101)	NIVO + IPI (n = 19)	Chemo (n = 11)
**Age**	Median (range), years	62 (21-86)	65 (26-87)	71 (35-82)	60 (41-74)
	< 65 years	117 (58)	46 (46)	9 (47)	6 (55)
**Sex**	Male	95 (47)	45 (45)	10 (53)	5 (45)
**Country**	Japan	13 (6)	7 (7)	13 (68)	7 (64)
	China	6 (3)	4 (4)	6 (32)	4 (36)
**ECOG PS**	0	111 (55)	52 (51)	12 (63)	7 (64)
**Disease stage at initial diagnosis**[a]	Stage IV	85 (42)	49 (49)	7 (37)	8 (73)
**Tumor sidedness**	Right	138 (68)	68 (67)	13 (68)	10 (91)
**Sites of metastases**[b,c,d]	Liver	76 (38)	42 (42)	5 (26)	5 (45)
	Lung	44 (22)	25 (25)	3 (16)	2 (18)
	Peritoneum	84 (42)	43 (43)	7 (37)	0
**Centrally confirmed MSI-H/dMMR status**	Yes	171 (85)	84 (83)	17 (89)	11 (100)
	No	31 (15)	17 (17)	2 (11)	0
**Tumor cell PD-L1 expression**[e,f]	< 1%	145 (72)	80 (79)	12 (63)	9 (82)
	≥ 1%	43 (21)	12 (12)	6 (32)	2 (18)
***BRAF*, *KRAS*, *NRAS* mutation status**[f,g]	*BRAF*/*KRAS*/*NRAS* wild-type	47 (23)	23 (23)	6 (32)	3 (27)
	BRAF mutant	52 (26)	24 (24)	4 (21)	2 (18)
	KRAS or *NRAS* mutant	43 (21)	21 (21)	6 (32)	2 (18)
	Unknown	55 (27)	31 (31)	2 (11)	4 (36)
**Clinical history of Lynch syndrome**[f,h]	Yes	22 (11)	17 (17)	0	1 (9)
	No	135 (67)	49 (49)	12 (63)	6 (55)
	Reported as unknown	44 (22)	30 (30)	7 (37)	4 (36)

Data are shown as n (%) unless otherwise noted. [a]All patients had stage IV disease at study entry. [b]Per BICR. [c]Metastases not reported in 3 patients in the NIVO + IPI arm. [d]Listed categories are not mutually exclusive as patients may have had multiple sites of metastases. [e]Tumor cell PD-L1 expression indeterminate, not evaluable, or not available: NIVO + IPI, n = 14 (n = 1 in Asian subpopulation); chemo, n = 9. [f]Percentages may not add up to 100% due to rounding. [g]*BRAF* and *KRAS*/*NRAS* mutant: NIVO + IPI, n = 5 (n = 1 in Asian subpopulation); chemo, n = 2. [h]Patients with Lynch syndrome not reported: NIVO + IPI, n = 1; chemo, n = 5.

Exposure and disposition

Disposition	Global population NIVO + IPI (n = 202)	Global population Chemo (n = 101)	Asian subpopulation NIVO + IPI (n = 19)	Asian subpopulation Chemo (n = 11)
All treated patients, n	200	88	19	7
Ongoing treatment,[a] n (%)	42 (21)	6 (7)	8 (42)	2 (29)
Completed treatment,[a] n (%)	62 (31)	0	5 (26)	0
Discontinued treatment,[a] n (%)	96 (48)	82 (93)	6 (32)	5 (71)
Disease progression	38 (19)	61 (69)	1 (5)	4 (57)
AE related to treatment	36 (18)	4 (5)	3 (16)	0
AE not related to treatment	12 (6)	5 (6)	1 (5)	0
Other[b]	10 (5)	12 (14)	0	1 (14)
Median duration of treatment (range), mo	13.5 (0–32.3)[c]	4.0 (0.1–27.5)	15.6 (1.3-32.3)[c]	5.4 (1.2-11.6)
Death,[a] n (%)	44 (22)	37 (42)	3 (16)	1 (14)
Disease progression	28 (14)	24 (27)	0	0
Other[d]	16 (8)	12 (15)	3 (16)	1 (14)

- Among patients in the Asian subpopulation treated with NIVO + IPI, 15 patients (88%) received all 4 doses of IPI
- Among patients in the Asian subpopulation treated with chemo, 7 patients (100%) received a biologic agent (bevacizumab, n = 6; cetuximab, n = 1)

In the chemo arm, 45 patients received crossover treatment upon disease progression by BICR. [a]Percentages shown are based on all treated patients. [b]Other reasons for discontinuation included death (n = 2 [n = 1 in Asian subpopulation]), withdrawal of consent (n = 1), pregnancy (n = 1), patient no longer met study criteria (n = 1), maximum clinical benefit (n = 8), and other reasons (n = 9 [n = 1 in Asian subpopulation]). [c]Median duration of treatment was 13.5 months (range, 0–32.3) for NIVO and 2.0 months (range, 0–3.7) for IPI in global population and 15.6 months (range, 1.3-32.3) for NIVO and 2.1 months (range, 0.7-3.0) for IPI in Asian subpopulation. [d]Other reasons for death included study drug-related toxicity (n = 2, both in the NIVO + IPI arm), other reasons (n = 17 [n = 1 in Asian subpopulation]), and unknown (n = 9 [n = 3 in Asian subpopulation]).

Progression-free survival

Global population

1L centrally confirmed MSI-H/dMMR	NIVO + IPI (n = 171)	Chemo (n = 84)
Median PFS,[a] mo	NR	5.9
95% CI	38.4-NE	4.4-7.8
HR (97.91% CI)	0.21 (0.13-0.35)	
P value	< 0.0001	

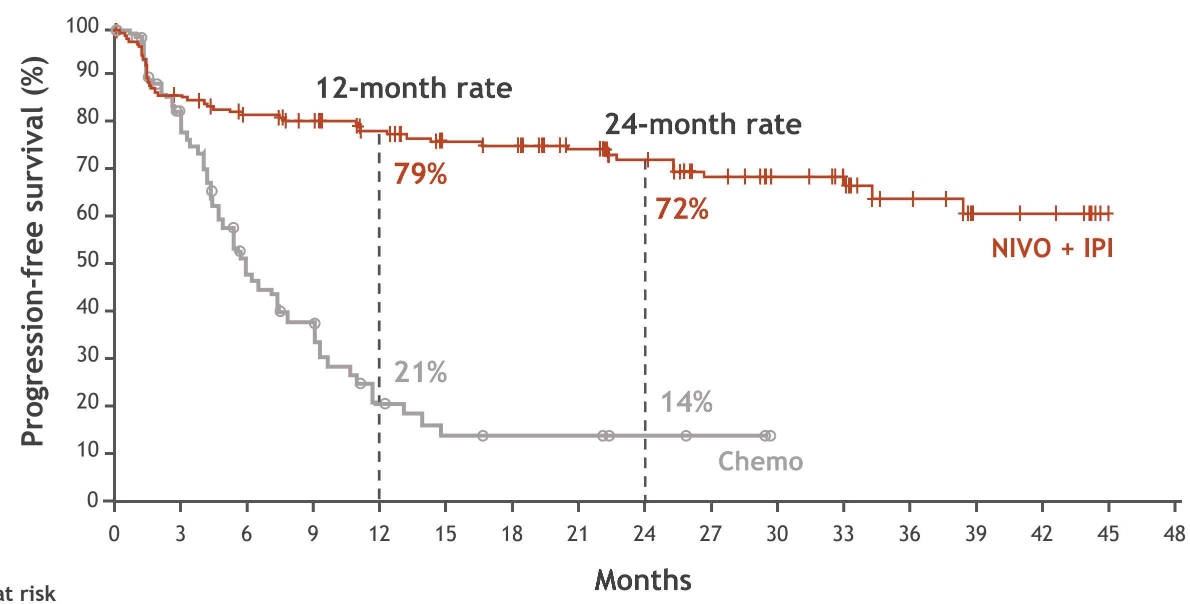

No. at risk																
NIVO + IPI	171	144	132	122	108	95	92	77	64	53	42	37	22	10	9	1	0
Chemo	84	53	29	20	10	6	5	5	3	2	0	0	0	0	0	0	0

Asian subpopulation

1L centrally confirmed MSI-H/dMMR	NIVO + IPI (n = 17)	Chemo (n = 11)
Median PFS,[a] mo	NR	7.4
95% CI	NE-NE	1.5-NE
HR (95% CI)	0.03 (< 0.01-0.28)	

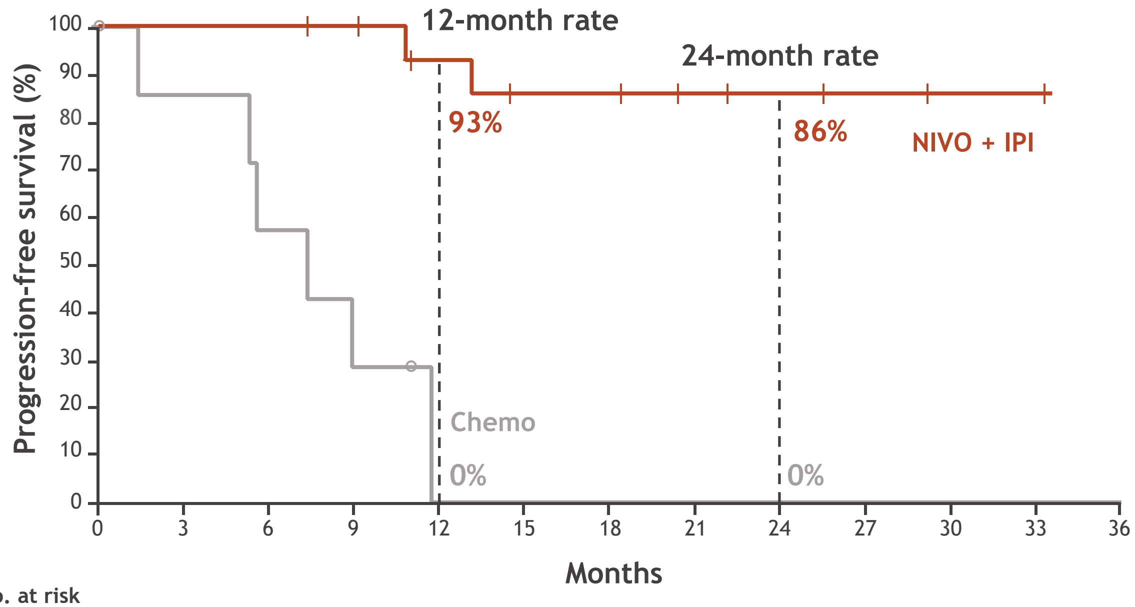

No. at risk													
NIVO + IPI	17	17	17	16	13	10	10	6	5	4	3	3	0
Chemo	11	6	4	3	0	0	0	0	0	0	0	0	0

- PFS benefit with 1L NIVO + IPI vs chemo was observed in the Asian subpopulation (HR, 0.03; 95% CI, <0.01-0.28)

[a]Per BICR.

Subsequent therapy

Subsequent therapy (1L centrally confirmed MSI-H/dMMR),[a-c] n (%)	Global population		Asian subpopulation	
	NIVO + IPI (n = 171)	Chemo (n = 84)	NIVO + IPI (n = 17)	Chemo (n = 11)
**Any subsequent therapy**	26 (15)	58 (69)	3 (18)	8 (73)
**Radiotherapy**	1 (< 1)	1 (1)	0	0
**Surgery**	5 (3)	4 (5)	0	0
**Systemic therapy**	20 (12)	57 (68)	3 (18)	8 (73)
Immunotherapy	7 (4)	56 (67)	3 (18)	7 (64)
On-study crossover to NIVO + IPI	0	39 (46)	0	4 (36)
Non-study immunotherapy	7 (4)	17 (20)	3 (18)	3 (27)
EGFR inhibitors	5 (3)	1 (1)	0	0
Platinum compounds	8 (5)	3 (4)	1 (6)	1 (9)
VEGFR targeted therapy	5 (3)	4 (5)	1 (6)	2 (18)
MEK, *NRAS*, and *BRAF* inhibitors	2 (1)	1 (1)	0	0
Other systemic anticancer therapy	12 (7)	5 (6)	1 (6)	1 (9)

- In the chemo arm of the Asian subpopulation, 7 patients (64%) received subsequent immunotherapy, including:
  - 4 patients (36%) who crossed over to on-study NIVO + IPI and 3 patients (27%) who received subsequent non-study immunotherapy

[a]Excludes surgery, radiotherapy, or non-study systemic therapy data collected on or after first crossover dose date. [b]Patients may have received more than 1 type of subsequent therapy. [c]Patients who received crossover treatment in the chemo arm are counted.

PFS2: progression-free survival after subsequent therapy

Global population

1L centrally confirmed MSI-H/dMMR	NIVO + IPI (n = 171)	Chemo (n = 84)
Median PFS2,[a,b] mo	NR	29.9
95% CI	NE-NE	14.8-NE
HR (95% CI)	0.27 (0.17-0.44)	

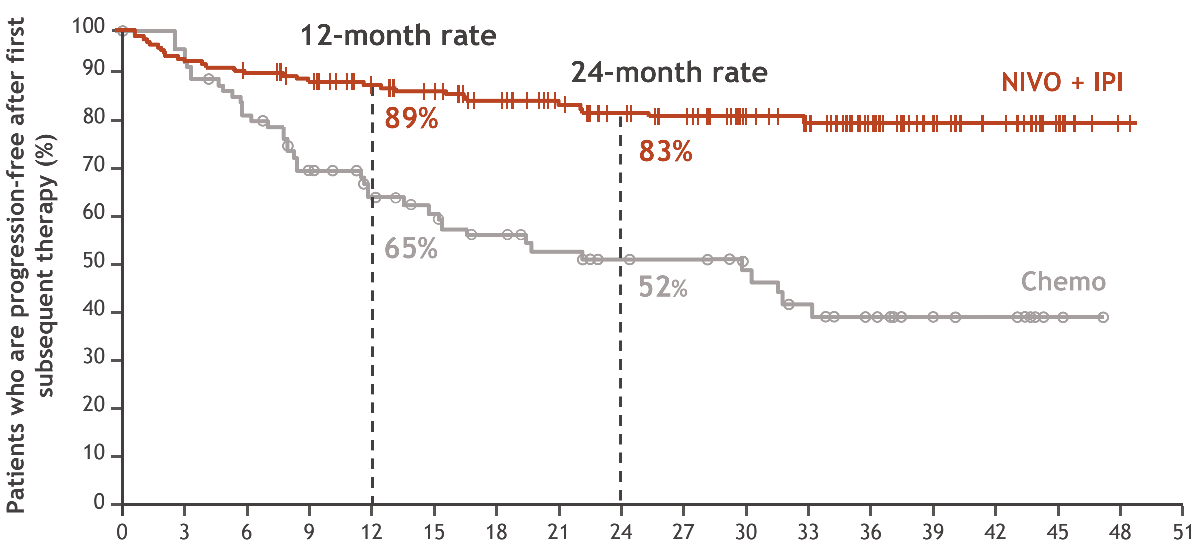

No. at risk																		
NIVO + IPI	171	161	155	147	135	127	117	103	94	85	71	64	45	30	25	10	1	0
Chemo	84	77	65	54	45	40	35	31	27	26	21	17	13	9	7	2	0	0

Asian subpopulation

1L centrally confirmed MSI-H/dMMR	NIVO + IPI (n = 17)	Chemo (n = 11)
Median PFS2,[a,b] mo	NR	NR
95% CI	32.8-NE	11.8-NE
HR (95% CI)	0.63 (0.09-4.52)	

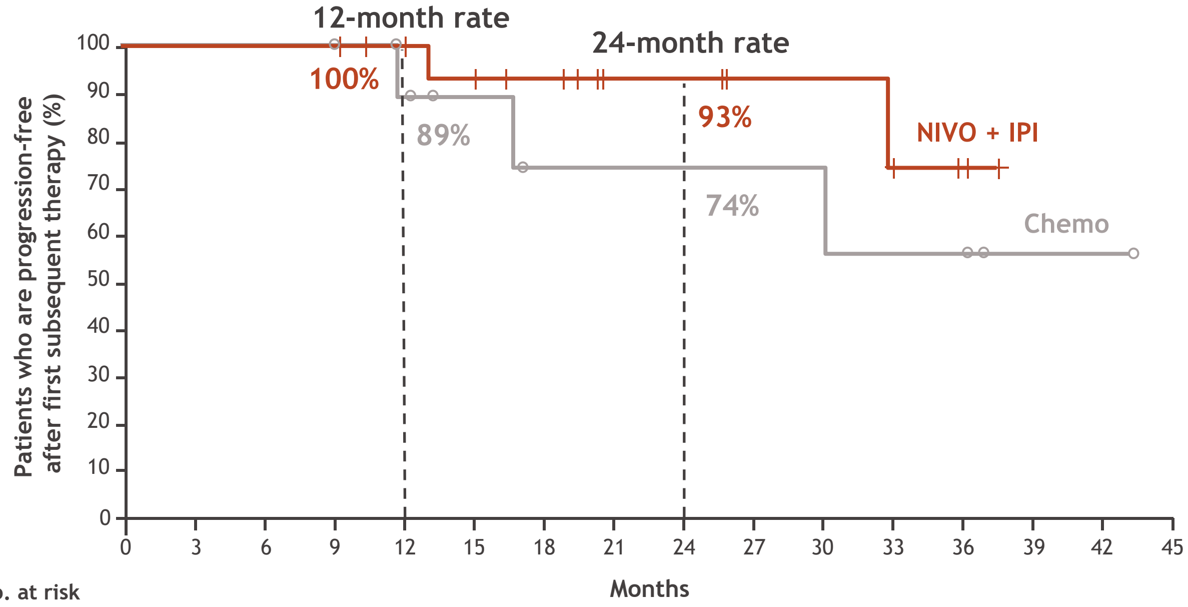

No. at risk																
NIVO + IPI	17	17	17	17	14	13	11	7	7	5	5	4	2	0	0	0
Chemo	11	11	11	11	8	6	4	4	4	4	3	3	3	1	1	0

- PFS2[a] favored NIVO + IPI vs chemo in the Asian subpopulation (HR, 0.63 [95% CI, 0.09–4.52])

[a]Defined as time from randomization to progression after subsequent systemic therapy, initiation of second subsequent systemic therapy, or death. [b]Per investigator.

Treatment-related adverse events

1L all treated patients, n (%)	Global population				Asian subpopulation			
	NIVO + IPI (n = 200)		Chemo[a] (n = 88)		NIVO + IPI (n = 19)		Chemo[a] (n = 7)	
	Any grade	Grade 3/4	Any grade	Grade 3/4	Any grade	Grade 3/4	Any grade	Grade 3/4
**TRAEs[b]**								
Any TRAEs	160 (80)	46 (23)	83 (94)	42 (48)	16 (84)	3 (16)	7 (100)	5 (71)
Serious TRAEs	38 (19)	32 (16)	17 (19)	14 (16)	2 (11)	1 (5)	2 (29)	2 (29)
TRAEs leading to discontinuation	33 (17)	23 (12)	28 (32)	9 (10)	2 (11)	1 (5)	1 (14)	0
**Treatment-related deaths**	2 (1)[c]		0[d]		0		0	
**TRAEs[b] reported in ≥ 15% of Asian patients**								
Adrenal insufficiency	20 (10)	6 (3)	0	0	5 (26)	1 (5)	0	0
Hypothyroidism	32 (16)	2 (1)	0	0	4 (21)	0	0	0
Decreased appetite	10 (5)	1 (< 1)	20 (23)	1 (1)	3 (16)	0	2 (29)	0
Hyperthyroidism	18 (9)	0	0	0	3 (16)	0	0	0
Malaise	2 (1)	0	2 (2)	0	2 (11)	0	2 (29)	0
Neutrophil count decreased	1 (< 1)	1 (< 1)	14 (16)	6 (7)	1 (5)	1 (5)	3 (43)	3 (43)
Diarrhea	42 (21)	2 (1)	45 (51)	4 (5)	0	0	3 (43)	0
Nausea	10 (5)	0	41 (47)	2 (2)	0	0	4 (57)	1 (14)
Peripheral sensory neuropathy	1 (< 1)	0	7 (8)	0	0	0	3 (43)	0
Platelet count decreased	0	0	6 (7)	0	0	0	2 (29)	0
Vomiting	4 (2)	0	18 (20)	1 (1)	0	0	2 (29)	1 (14)
White blood cell count decreased	0	0	4 (5)	1 (1)	0	0	2 (29)	1 (14)

- The most common any-grade TRAEs occurring in ≥ 15% of patients in the Asian subpopulation were
  - NIVO + IPI: adrenal insufficiency (26%), hypothyroidism (21%), and decreased appetite (16%)
  - Chemo: nausea (57%), diarrhea (43%), neutrophil count decreased (43%), and peripheral sensory neuropathy (43%)

[a]Events that occurred during the crossover period were not included in the reported data. [b]Includes events reported between first dose and 30 days after last dose of study therapy. [c]Includes 1 event each of myocarditis and pneumonitis. [d]One death (acute myocarditis) was related to crossover treatment.

Immune-mediated adverse events

IMAEs[b] (1L all treated patients), n (%)	Global population				Asian subpopulation			
	NIVO + IPI (n = 200)		Chemo[a] (n = 88)		NIVO + IPI (n = 19)		Chemo[a] (n = 7)	
	Any grade	Grade 3/4	Any grade	Grade 3/4	Any grade	Grade 3/4	Any grade	Grade 3/4
Adrenal insufficiency	21 (11)	7 (4)	0	0	5 (26)	1 (5)	0	0
Hypothyroidism	33 (17)	2 (1)	1 (1)	0	4 (21)	0	0	0
Hyperthyroidism	18 (9)	0	1 (1)	0	3 (16)	0	0	0
Rash	11 (6)	3 (2)	0	0	2 (11)	0	0	0
Diarrhea/colitis	13 (7)	9 (5)	1 (1)	0	1 (5)	0	0	0
Hepatitis	11 (6)	6 (3)	0	0	0	0	0	0
Hypophysitis	10 (5)	5 (3)	0	0	0	0	0	0
Pneumonitis	4 (2)	3 (2)	0	0	0	0	0	0
Thyroiditis	3 (2)	1 (< 1)	0	0	0	0	0	0
Diabetes mellitus	2 (1)	0	0	0	0	0	0	0
Nephritis and renal dysfunction	2 (1)	1 (< 1)	0	0	0	0	0	0
Hypersensitivity	0	0	1 (1)	1 (1)	0	0	0	0

- In the Asian subpopulation, the majority of IMAEs were grade 1 or 2, with only a single grade 3/4 IMAE reported (adrenal insufficiency)

[a]Events that occurred during the crossover period were not included in the reported data. [b]IMAEs are specific events considered as potential immune-mediated events by investigator, occurring within 100 days after the last dose of study treatment, regardless of causality, and, with the exception of endocrine events, are treated with immune-modulating medication.

Summary

- 1L NIVO + IPI demonstrated clinically meaningful PFS benefit vs chemo in the subpopulation of Asian patients with centrally confirmed MSI-H/dMMR mCRC (HR, 0.03 [95% CI, < 0.01–0.28])
  - 24-month PFS rates for NIVO + IPI vs chemo: 86% vs 0%
- PFS2 favored NIVO + IPI vs chemo in Asian patients (HR, 0.63 [95% CI, 0.09–4.52]), suggesting clinical benefit is maintained after subsequent therapy
  - 24-month PFS2 rates for NIVO + IPI vs chemo: 93% vs 74%
- The safety profile of NIVO + IPI was consistent with the known profiles of each individual component, with no new safety signals in Asian patients
- **Consistent with data from the global patient population, these results provide evidence to support NIVO + IPI as a standard-of-care 1L treatment option for Asian patients with MSI-H/dMMR mCRC**

Investigators

We would like to acknowledge the investigators of the CheckMate 8HW study

Argentina

J Grasselli
R Kowalyszyn
LC Lupinacci
GA Mendez
JM O Connor

Australia

R Joshi
R Ladwa
A Nagrial
A Strickland
NC Tebbutt

Austria

K Aichberger
A Gerger
R Greil
B Gruenberger
G Prager
H Rumpold

Belgium

P-J Cuyle
A Hendlisz
F Sclafani
E Van Cutsem

Chile

O Barajas
S Mondaca
L Villanueva

Brazil

KC Abdalla
AC Bragagnoli
MIFM Braghiroli
CAL de Mello
JCSO de Souza
GC Girotto
J de Janoski Menezes
CMV Moniz
SCS Oliveira
RSP Reichelmann
FA Santos
L de Souza Viana

Canada

F Aubin
R Burkes
H Karachiwala
F Lemay
D Renouf
X Zhu

France

T Andre
P Artru
F Audemar
M Ben Abdelghani
J Bennouna
C Borg
A Carnot
C Coutzac
L Dahan
C de la Fouchardiere
F Ei Hajbi
R Guimbaud
E Samalin-Scalzi
D Smith
F Thuillier
Y Touchefeu
D Tougeron

China

M Cai
C Chen
H Chen
K Chen
Z Chen
W Cui
Y Deng
Y Gao
G Han
W Hu
Q Li
W Li
Y Li
Y Li
J Liu
Z Niu
Q Sun
W Wang
J Xiong
F Ye
X Yin
Y Yuan
J Zhang
L Zhang
X Zhang
Y Zhang
Q Zou

Czech Republic

S John
B Melichar
J Tomasek
D Vrana

Denmark

LH Jensen
SE Nielsen

Germany

D Arnold
G Folprecht
E Goekkurt
V Heinemann
S Kasper-Virchow
G Martin Haag
JU Riera-Knorrenschild
A Vogel

Greece

E Bournakis
M Karamouzis
D Mauri
D Mavroudis
G Pentheroudakis

Italy

R Bordonaro
F Ciardiello
S Lonardi
S Siena
A Sobrero
G Tortora

Ireland

D Gallagher
R McDermott
N Osman

Netherlands

C Grootscholten
J Roodhart
D Sommeijer

Japan

H Baba
H Bando
T Denda
T Esaki
T Hamaguchi
H Hara
T Kato
Y Komatsu
D Kotani
N Machida
T Masuishi
Y Miura
Y Miyamoto
T Nishina
K Ohtsubo
Y Sakamoto
T Satoh
M Shiozawa
Y Sunakawa
K Suyama
A Takashima
H Taniguchi
K Yamaguchi
K Yamazaki
M Yokota
N Yoshida

Norway

MG Guren
H Hamre
H Sorbye

Turkey

T Cil
M Ozguroglu

Romania

E Banu
TE Ciuleanu
AE Croitoru
B Gafton
DE Ganea
M Schenker

Spain

RG Carbonero
EE Fernandez
S Gil Calle
B Grana Suarez
ML Limon Miron
JL Manzano Mozo
MJ Safont Aguilera

United Kingdom

D Church
N Diamantis

United States

M Cho
AL Cohn
MD Kochenderfer
A Krishnamurthy
H-J Lenz
AS Paulson
NH Segal
SH Shao
RS Siegel
KW Tam

Puerto Rico (US)

M Cruz-Correa

Acknowledgments

- Patients, investigators, and research staff who made this study possible
- The clinical study team and the global trial manager, Janice Kaps-Trotter; Carine Cabilla for clinical operations support; Translational Medicine and Precision Medicine teams (Bristol Myers Squibb) for central MMR/MSI testing and diagnostics support
- Agilent Technologies, Inc. for collaborative development of the MMR IHC panel pharmDx (Dako Omnis) assay (Santa Clara, CA) and Biocartis NV for collaborative development of the Idylla™ MSI test (Mechelen, Belgium)
- Supported by Bristol Myers Squibb (Princeton, NJ) and Ono Pharmaceutical Co., Ltd. (Osaka, Japan)
- All authors contributed to and approved the presentation; writing and editorial assistance were provided by Christopher Spencer, PhD, of Parexel, funded by Bristol Myers Squibb

Abbreviations

1L = first line

AE = adverse event

BICR = blinded independent central review

BRAF = V-raf murine sarcoma viral oncogene homolog B

chemo = chemotherapy

CI = confidence interval

CRC = colorectal cancer

dMMR = mismatch repair-deficient

ECOG PS = Eastern Cooperative Oncology Group performance status

EGFR = epidermal growth factor receptor

FOLFIRI = leucovorin (folinic acid)/ fluorouracil/irinotecan

HR = hazard ratio

IHC = immunohistochemistry

IMAE = immune-mediated adverse event

IPI = ipilimumab

KRAS = Kirsten rat sarcoma viral oncogene

mCRC = metastatic colorectal cancer
